# A large carnivorous mammal from the Late Cretaceous and the North American origin of marsupials

**DOI:** 10.1038/ncomms13734

**Published:** 2016-12-08

**Authors:** Gregory P. Wilson, Eric G. Ekdale, John W. Hoganson, Jonathan J. Calede, Abby Vander Linden

**Affiliations:** 1 Department of Biology and Burke Museum, University of Washington, Seattle, Washington 98195, USA; 2 Department of Biology, San Diego State University, San Diego, California 92182, USA; 3 Department of Paleontology, San Diego Natural History Museum, San Diego, California 92101, USA; 4 North Dakota Geological Survey, Bismarck, North Dakota 58505, USA; 5 Graduate Program in Organismic and Evolutionary Biology, University of Massachusetts, Amherst, Massachusetts 01003, USA

## Abstract

Marsupial mammal relatives (stem metatherians) from the Mesozoic Era (252–66 million years ago) are mostly known from isolated teeth and fragmentary jaws. Here we report on the first near-complete skull remains of a North American Late Cretaceous metatherian, the stagodontid *Didelphodon vorax*. Our phylogenetic analysis indicates that marsupials or their closest relatives evolved in North America, as part of a Late Cretaceous diversification of metatherians, and later dispersed to South America. In addition to being the largest known Mesozoic therian mammal (node-based clade of eutherians and metatherians), *Didelphodon vorax* has a high estimated bite force and other craniomandibular and dental features that suggest it is the earliest known therian to invade a durophagous predator–scavenger niche. Our results broaden the scope of the ecomorphological diversification of Mesozoic mammals to include therian lineages that, in this case, were linked to the origin and evolution of marsupials.

## Abstract (web summary)

The early fossil record of metatherian mammals, the group including marsupials, is limited. Here, Wilson and colleagues describe a fossil skull of the Late Cretaceous metatherian *Didelphodon vorax*, providing insight into the ecology of this species as well as the North American origin of marsupials.

Marsupial mammals are today overshadowed by placentals in diversity and geographic extent^1^. However, before the Cretaceous–Palaeogene mass extinction 66 million years ago (Myr ago), metatherians (the stem-based clade of living marsupials and their closest relatives^2^) ranged widely across northern landmasses; they also surpassed eutherians (the stem-based clade of living placentals and their closest relatives^3^) in both taxonomic and morphological richness^4^,^5^,^6^. Although studies of comparative development^7^ and physiology^8^ have shed light on constraints that might have influenced this clade changeover, critical gaps in our understanding of early metatherian evolution can only be addressed with more anatomically complete fossils of these taxa. Unfortunately, skulls of early metatherians are scarce and distributed unevenly across time, space and phylogeny. Of the 69 Cretaceous species, relatively complete skulls have been described for only four Asiatic taxa, all phylogenetically distant from the origin of marsupials^4^,^9^. The 60 species from North America (NA) are known almost exclusively from teeth and fragmentary jaws^4^. Consequently, previous phylogenetic analyses of early metatherians have either relied on mostly dental data^6^,^10^ or excluded most NA Late Cretaceous stem metatherians^11^. Their results have implied that the clade of NA Late Cretaceous stem metatherians diverged early (>100 Myr ago) from the lineage leading to marsupials and the latter predominantly evolved in South America (SA) (but see ref. 12 for an opposing non-cladistic viewpoint). Until now, it has been unclear whether this scenario reflects (i) a meaningful evolutionary pattern, in which NA Late Cretaceous metatherians had only minor influence on the origin of marsupials or (ii) an artefact of incomplete fossil data and/or inadequate analysis.

The four specimens described herein, a near-complete skull (NDGS 431), a snout (UWBM 94084) and two maxillae (UWBM 94500, SCNHM VMMa 20) from the Hell Creek Formation (upper Maastrichtian, 69–66 Myr ago) of Montana and North Dakota, are assigned to the stagodontid metatherian *Didelphodon vorax* on the basis of cheek-tooth features. This taxon has long captivated palaeontologists^13^,^14^,^15^ as the largest Cretaceous metatherian with shearing molars and bulbous premolars, leading to the hypothesis of its specialized role in Late Cretaceous food webs, perhaps as a predator or scavenger capable of crushing hard objects (for example, bone, shell)^5^,^14^,^15^,^16^. Postcranial elements attributed to *D*. *vorax* have spurred further speculation that this taxon was semiaquatic^17^ (but see ref. 14).

We incorporate new anatomical data from these four specimens of *D. vorax* into a phylogenetic analysis that show that marsupials or their closest relatives evolved in NA, as part of a Late Cretaceous diversification of metatherians, and soon thereafter dispersed to SA. We also show that *D*. *vorax* is the largest known Mesozoic therian mammal and has a high estimated bite force and other craniomandibular and dental traits that suggest it is the earliest known therian to evolve durophagous predator–scavenger behaviour. Taken together, these results indicate that the ecomorphological diversification of Mesozoic mammals^18^ extended to therian lineages, including those near the origin of marsupials.

## Results

### Description

**Figure 1:**
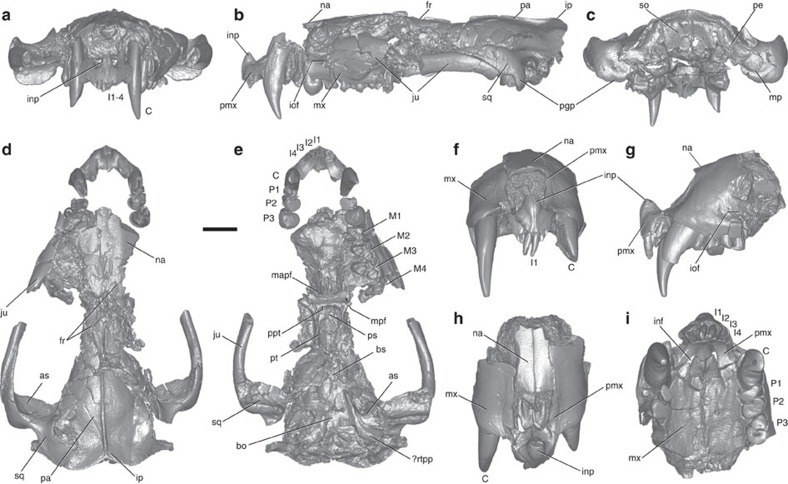
Skull of the Late Cretaceous marsupialiform *D. vorax*. Specimen NDGS 431, in anterior (**a**), left lateral (**b**), posterior (**c**), dorsal (**d**) and ventral (**e**) views, and specimen UWBM 94084, in anterior (**f**), left lateral (**g**), dorsal (**h**) and ventral (**i**) views. All images from digitally rendered, micro-CT scans. as, alisphenoid; bo, basioccipital; bs, basisphenoid; C, upper canine; I1–4, upper incisors 1–4; inp, internarial process; inf, incisive foramen; iof, infraorbital foramen; ip, interparietal; ju, jugal; M1–4, upper molars 1–4; mapf, major palatine fenestra; mp, mastoid process; mpf, minor palatine foramen; mx, maxilla; na, nasal; P1–3, upper premolars 1–3; pa, parietal; pe, petrosal; pgp, postglenoid process; pmx, premaxilla; ppt, postpalatine torus; ps, presphenoid; pt, pterygoid; rtpp, rostral tympanic process of the petrosal; so, supraoccipital; sq, squamosal. Scale bar, 10 mm.

**Figure 2:**
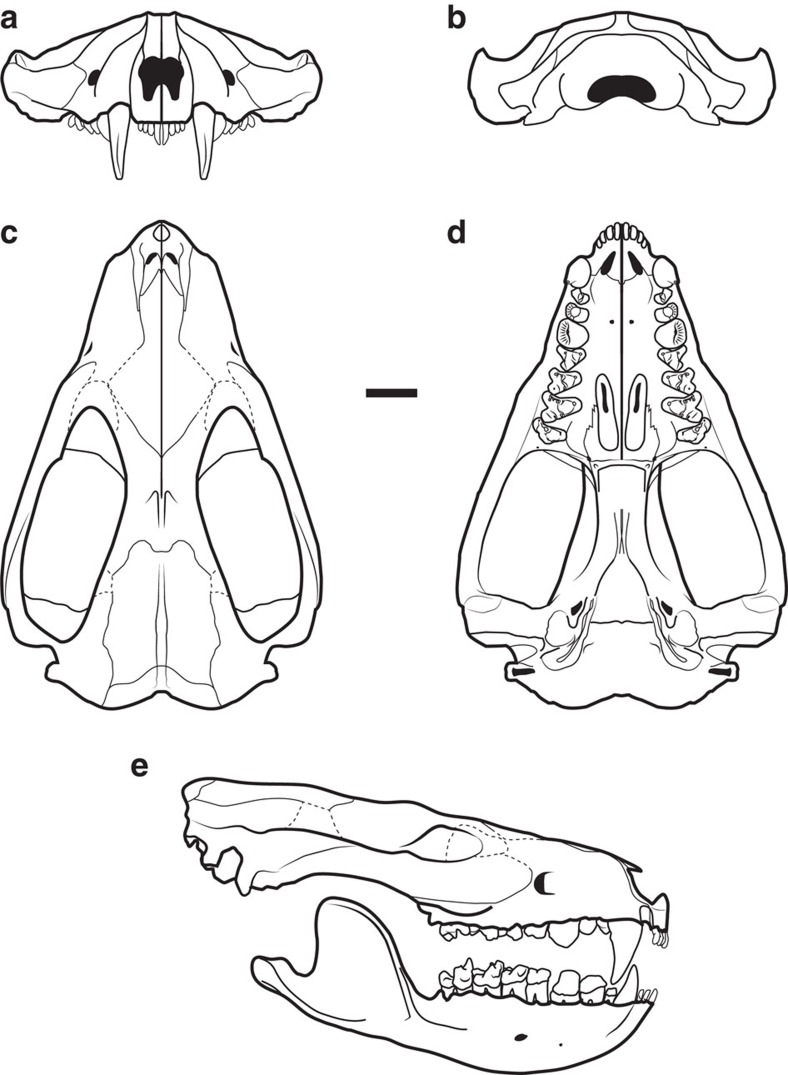
Reconstruction of the skull and jaw of *D. vorax*. The reconstruction is shown in anterior (**a**), posterior (**b**), dorsal (**c**), ventral (**d**), and right lateral (**e**) views. It is based on NDGS 431 (basicranium, palate, skull roof and dentition), UWBM 94084 (rostrum), UWBM 94500 (palate and maxillo-jugal contact), SCNHM VMMa 20 (maxillo-jugal contact) and UWBM 102139 (dentary). The areas not preserved in the actual specimens include the lower incisors, some upper incisors, parts of the orbitotemporal and occiput regions, and some sutures. Uncertainties in sutures are represented by dashed lines. Scale bar, 10 mm.

In dorsal view (Figs 1d,h and 2c), the skull is broad and triangular with a short rostrum (∼1/3 skull length), robust, wide zygomata, narrow postorbital breadth, small braincase, small sagittal crest and well-developed lambdoidal crest. Lacking a prominent postorbital process, the orbital region is confluent with the temporal region. In lateral view (Figs 1b,g and 2e), the skull has a low, flat profile, even after post-mortem deformation is accounted for. The skull length of the younger adult individual (NDGS 431) is 94.7 mm and its maximum width is 59.3 mm; the mature adult (UWBM 94084) scales to 122.1 and 77.4 mm, respectively (see also Supplementary Figs 1–10, Supplementary Tables 1–3 and Supplementary Note 1 for additional figures, measurement data and extended description of the specimens).

### Phylogenetic relationships

**Figure 3:**
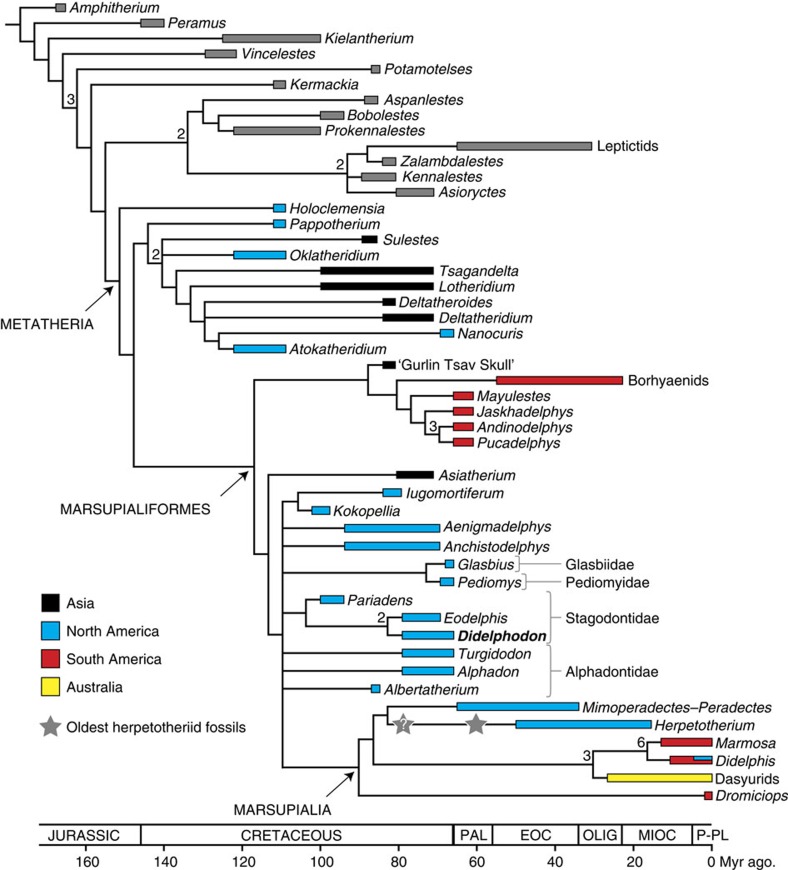
Phylogenetic relationships of *D. vorax*. The strict consensus tree of 18 equally parsimonious trees (tree length 601; consistency index=0.364; retention index=0.667) derived from analysis of 164 characters and 48 taxa, with a focus on early metatherians. *Didelphodon* is in boldface type. Bremer support values greater than one are shown above the node. Thin black lines represent phylogenetic relationships, and coloured bars indicate temporal ranges of taxa, with colours indicating the geographic distribution of metatherian taxa (black for Asia, blue for NA, red for SA and yellow for Australia). Grey stars represent the oldest putative herpetotheriid fossils, with the query indicating taxonomic uncertainty (see Methods). See Supplementary Note 3 and Supplementary Data 1 and 2 for details of taxon and character lists, data matrix, phylogenetic methods and synapomorphy lists. PAL, Paleocene; EOC, Eocene; OLIG, Oligocene; MIOC, Miocene; P-PL, Pliocene-Pleistocene.

As a result of the specimens reported herein, the skull of *D. vorax* is the best-known among all Cretaceous metatherians. To understand the impact of these new data, we performed a parsimony analysis of dental and craniomandibular characters (Supplementary Note 2) scored for 48 metatherians and closely related taxa^9^,^10^. For the first time, all relevant Cretaceous and Palaeogene metatherian taxa known by relatively complete skulls were included in the same data matrix (Supplementary Data 1). We obtained 18 equally parsimonious trees with tree length=601, consistency index=0.364 and retention index=0.667. Our resulting strict consensus tree (Fig. 3 and Supplementary Note 3) agrees in most aspects with previous analyses^10^,^11^, including support for a monophyletic Stagodontidae, in which the early Campanian–early Maastrichtian *Eodelphis* and the Albian–Cenomanian *Pariadens* are successive sister taxa to *Didelphodon*. We fail to recover a monophyletic Australidelphia (dasyurids+*Dromiciops*), in contrast with the weight of previous morphological and molecular phylogenetic studies^19^, but this might be an artefact of sparse sampling of these clades in our analysis. Placement of *Herpetotherium* and *Mimoperadectes*–*Peradectes* in the crown clade is novel but differs only slightly from previous topologies^11^,^20^,^21^. A more striking result is that the main dichotomy within Marsupialiformes places NA Cretaceous stem taxa (including *Didelphodon*) with marsupials to the exclusion of SA Palaeogene stem taxa (for example, borhyaenids, *Mayulestes* and *Pucadelphys*) and the ‘Gurlin Tsav skull'. Bremer support for this topology is low, but alternatives are several steps longer and, in some cases, significantly different (Supplementary Note 4). Synapomorphies for this clade of *Asiatherium*, NA stem marsupialiforms and marsupials are (i) double-rank prevallum/postvallid shearing; a lower molar with (ii) talonid subequal to wider than trigonid and (iii) entoconid subequal to larger than hypoconid and/or hypoconulid; and presence of (iv) a lacrimal tubercle and (v) postpalatine torus (Supplementary Data 2). Homoplasy is prevalent within Marsupialiformes (low consistency index) and dental features in particular seem prone to homoplasy; for example, the ‘Gurlin Tsav skull'+SA stem marsupialiforms and the Stagodontidae convergently evolved features, such as a well-developed upper-molar postmetacrista, presumably as adaptations for carnivory^15^. However, the relatively high retention index implies that homoplasies often serve as synapomorphies in our analysis and contribute to the moderately resolved consensus tree.

### Palaeobiology

Body mass estimates from total-skull length (TSL) and upper-molar-row length^22^ show that *D. vorax* ranged from 2.4 kg (2.1–2.7 kg) for the young adult individual (NDGS 431) to 5.2 kg (4.2–6.2 kg) for the older adult individual (UWBM 94084, Supplementary Table 4). Using the body mass estimate of the older adult individual and a formula derived from modern comparative data^23^, we estimated the maximum prey size of *D*. *vorax* to have been 5.0 kg (95% confidence intervals=2.2–11.4 kg; Supplementary Table 5).

The estimated maximal bite force at the canines (CB_S_), which is based on measurements of the skull model of the younger adult individual (NDGS 431), is 218 N (Supplementary Fig. 11 and Supplementary Tables 6 and 7). To account for the body size scaling relationship of CB_S_ and to allow for comparisons across a broader size range of mammals^24^, we also calculated the canine bite force quotient (BFQ). Whereas the CB_S_ of *D*. *vorax* is lower than that of most larger-bodied extant and fossil taxa, its BFQ of 201 exceeds that of all other measured taxa.

The relative premolar size (RPS) of *D. vorax* ranges between 3.27 and 4.99 (Supplementary Table 8); it overlaps or exceeds the RPS of extant carnivorans and dasyuromorph marsupials with crushing capabilities (RPS=3.00–3.90)^25^,^26^, and the RPS of the early Palaeocene archaic ungulate *Periptychus carinidens* (body mass=10.1 kg; p4 width=9.6 mm; RPS=4.44), which is also a putative hard-object feeder^27^.

The canine bending strength estimates of *D*. *vorax* are proportionally greater than those for extant canids and more similar to those for extant felids and hyaenids (Supplementary Fig. 12 and Supplementary Tables 9 and 10). Bending strength estimates about the mediolateral axis (*Sy*) of the canine suggest that *D*. *vorax* generated proportionally greater bite forces at the canines compared with canids^28^, corroborating the BFQ results. The high estimates about the anteroposterior axis (*Sx*) suggest that the canines of *D*. *vorax* likely could have withstood substantial oblique or mediolateral stresses induced from either contact with bone during a deep bite, struggling prey during a killing bite or bone crushing performed by adjacent teeth^28^.

The mandibular bending strength estimates of *D*. *vorax* are greater than those of the Virginia opossum *Didelphis virginiana* but less than those of the Tasmanian devil *Sarcophilus harrisii* (Supplementary Fig. 13 and Supplementary Table 11). Loads in the dorsoventral plane (*Zx*/*L*, Supplementary Fig. 13a) generally correspond to bite forces, which tend to scale with body size^29^; thus, these results are also consistent with results from our bite force analysis. Dorsoventral buttressing in *D*. *vorax* specifically increases below the ultimate premolar and molars, likely to accommodate larger stresses induced by crushing hard objects with its premolars. The capacity to withstand large labiolingual loads, as seen in the mandible of *D*. *vorax* (*Zy*/*L*, Supplementary Fig. 13b), often reflects the need to resist lateral movements of a prey animal struggling under the bite of a predator or from torsional stresses induced by hard-object feeding (for example, bones and shells)^29^. The relative force profile (Supplementary Fig. 13c) shows that while the mandible of *D. vorax* was adapted to withstand large bite forces posteriorly, it also maintained strong buttressing against labiolingual forces to deal with the torsional stresses noted above (*Zx*/*Zy*<2.00).

The results of the dental microwear analysis (Supplementary Fig. 14 and Supplementary Tables 12–15) show that all specimens of *D. vorax* cluster together and that the range of variation is similar to that observed within many extant taxa (for example, *Bassariscus astutus*). *D. vorax* clusters with extant specimens with high numbers of puncture pits and hypercoarse scratches, low numbers of pits and average numbers of scratches (that is, principal coordinate analysis (PCoA) 1 scores near average and negative PCoA 2 scores). Although the presence of numerous puncture pits on the enamel surface of *D. vorax* is consistent with a hard-food diet, similar numbers of puncture pits are also found in animals that do not have a hard-food diet (for example, *Ondatra zibethicus*) and the number of pits is lower than that expected in a durophagous taxon. The microwear signature of *D. vorax* overlaps those of omnivorous taxa (for example, *Procyon lotor*), as well as carnivores and animal-dominated omnivores (for example, *Genetta tigrina* and *Lontra canadensis*). It also overlaps with the microwear signatures of two plant-dominated omnivores: *Procyon cancrivorus,* which consumes hard-shelled crustaceans and arthropods as well as small vertebrates, and *O. zibethicus*, a semi-aquatic rodent that feeds on aquatic vegetation but also crayfish, molluscs and fish. The microwear signature of *D. vorax* differs from that of taxa that primarily feed on insects, spiders and annelids (*Antechinus stuarti*, *Tupaia glis* and *Elephantulus rufescens*), which are characterized by a higher number of pits. Despite clustering near some of the extant taxa analysed, the diet of *D. vorax* cannot be tightly matched with the diet of any specific extant taxon. The microwear results suggest that *D. vorax* was an omnivore that likely consumed a range of vertebrate, plant and hard-shelled invertebrate resources but few insects, spiders and annelids.

## Discussion

Our results show that five major marsupialiform lineages, the Glasbiidae, Pediomyidae, Stagodontidae, Alphadontidae and marsupials (including *Mimoperadectes–Peradectes*+*Herpetotherium*), diverged in NA by the Albian–Cenomanian (100 Myr ago) to late Santonian (85 Myr ago). Accompanying this taxonomic radiation, and consistent with other recent findings^30^, were increases in marsupialiform body size (up to 5.2 kg) and dietary diversity, from insectivory to omnivory, frugivory and carnivory, consuming a range of soft to hard food items^5^. This ecomorphological expansion was broadly coincident with and perhaps related to the onset of adaptive radiations in multituberculates and flowering plants^31^. Most of this NA marsupialiform diversity, however, was lost gradually from the late Campanian to Maastrichtian (79–66 Myr ago)^6^ and then abruptly during the Cretaceous–Palaeogene mass extinction (66 Myr ago)^5^,^6^. Meanwhile, the focus of marsupialiform evolution shifted to SA. Palaeocene SA stem marsupialiforms represent their own monophyletic radiation, rather than successive outgroups to crown marsupials, with implied ghost lineages that stretch back to at least the early Campanian (84–81 Myr ago) in Asia and presumably NA, considering its intermediate position between Asia and SA. Likewise, our results push the origin of crown marsupials back to the early Palaeocene (65 Myr ago), based on the age of the oldest known fossils of *Peradectes*^6^,^32^, and possibly to the late Campanian (79–74 Myr ago), depending on the herpetotheriid affinities of certain fossils^6^. The latter age would be at least 20 Myr ago older than the earliest definitive marsupial fossils not in the Peradectidae or Herpetotheriidae (Itaboraian of Brazil, ca. 54–52 Myr ago)^33^, but would be congruent with recent molecular estimates^34^. The NA roots for multiple, successive outgroups also constrain marsupials or a recent ancestor to a NA origin and dispersal to SA during the Late Cretaceous or earliest Palaeocene—an inference corroborated by other biotic connections (multituberculates and hadrosaurines) and geological evidence of a land connection between NA and SA^33^.

Novel morphology from the specimens reported herein broadens the ecological diversity of NA Cretaceous metatherians. The new specimens support the hypothesis that *D. vorax* was a powerful predator–scavenger^5^,^14^,^15^. New body mass estimates show that, among Mesozoic mammals, *D*. *vorax* was larger than all other therians and rivaled by only a few non-therians^35^. The body mass estimate of 5.2 kg for UWBM 94084 (Supplementary Table 4) is comparable in size to the Virginia opossum *D. virginiana* (1.9–6.0 kg) and American badger *Taxidea taxus* (4.0–12.0 kg). Owing in part to energetic constraints, small- to medium-sized extant predators (<15 kg) mostly feed on small vertebrates and invertebrates that are a small fraction of their own body mass^36^. Accordingly, an adult *D*. *vorax* would have preyed on mainly small Late Cretaceous molluscs, fish, amphibians, lizards, turtles and mammals; however, its maximum prey size is estimated to have been 5.0 kg (95% confidence intervals of 2.2–11.4 kg; Supplementary Table 5), which would have included some small and juvenile dinosaurs.

Analysis of skull biomechanics points to an even wider predatory range for *D. vorax* than would be expected from its body size alone. Its relatively short, broad and robust skull conferred mechanical advantages via shorter jaw out levers, more space in the temporal region for jaw adductor musculature and increased resistance to torsional loadings^8^. Its estimated maximal bite force at the canines (CB_S_=218 N) is greater than that of most small- to medium-sized extant mammals (<15 kg) and only slightly less than that of the river otter *L. canadensis* (233 N) and European badger *Meles meles* (244 N; Supplementary Tables 6 and 7). The canine BFQ of *D*. *vorax* (BFQ=201) indicates that its ‘pound-for-pound' relative bite force exceeded that of all other measured extant and fossil mammals, including the marsupial lion *Thylacoleo carnifex*† (193), Tasmanian devil *S. harrisii* (166), dire wolf *Canis dirus*† (157), African lion *Panthera leo* (116) and spotted hyaena *Crocuta crocuta* (114; Supplementary Table 7). BFQs greater than 100 typically only occur in bone-cracking specialists and taxa that occasionally hunt or scavenge on prey larger than themselves, such as the honey badger *Mellivora capensis*, striped hyaena *Hyaena hyaena* and spotted hyaena *C. crocuta*^23^,^24^.

Other craniodental features strengthen our inference that *D. vorax* was durophagous and possibly osteophagous. Its broad, convex premolars with the protocone as a stress concentrator closely approximate an ‘ideal' crushing form^37^. They require less force than concave shapes to induce crack formation in prey items and are more resistant to tooth fracture than tall- or narrow-cusped shapes^37^. Even the flat premolars of later wear stages, while not as effective at crushing, incur lower strain values^37^. Moreover, the RPS^25^ of *D*. *vorax* (RPS of P3=3.27–4.99; Supplementary Table 8) matches or exceeds those of extant carnivorans and dasyuromorph marsupials with crushing capabilities (RPS=3.00–3.90)^25^. The rounded cross-sectional shape of the canines of *D*. *vorax* resembles those of extant hyaenids and extinct bone-crushing dogs (borophagines) and dire wolves^28^ (Supplementary Fig. 12 and Supplementary Tables 9 and 10). This canine shape resists bending in all directions, for example, from deep bites contacting bone or from stresses incurred from adjacent premolars crushing hard objects^28^. *D*. *vorax* also has a robust lower jaw that exhibits strong dorsoventral buttressing below the ultimate premolar and molars, likely to accommodate large stresses induced by crushing hard objects with the premolars^29^. Anteriorly, the cross-sectional shape of the lower jaw is more rounded and likely reflects strengthening of the symphysis for crushing with the anterior teeth and/or for handling torsional stresses of struggling prey^29^ (Supplementary Fig. 13 and Supplementary Table 11). Antemortem canine tooth breakage, such as in UWBM 94084 and NDGS 431, is also significantly more common in durophagous mammals than in carnivores, insectivores or omnivores^38^.

However, the skull of *D. vorax* differs from those of highly specialized bone crackers. While its reconstructed cranial profile is relatively tall at the snout, as in the molluscivorous sea otter *Enhydra lutris*, it lacks the characteristic vaulted forehead of hyaenids, percrocutids and borophagines that dissipates the large forces generated from cracking bones^39^,^40^,^41^. It is possible that (1) *D*. *vorax* was an early-stage bone-cracking specialist^39^ that had relatively little time (10 Myr)^15^ to evolutionarily diverge from its late Campanian sister genus (*Eodelphis*) with smaller, trenchant premolars and a gracile lower jaw^15^; (2) the evolutionary constraints that shaped the cranial morphology of the metatherian *D*. *vorax* differed from those of eutherian bone-cracking specialists^8^; or (3) akin to non-bone-cracking canids (for example, wolves)^24^,^40^, *D. vorax* did not regularly deploy its high bite force because its skull was not well adapted for very high stresses; instead, *D*. *vorax* had a broad dietary range that included hard-object food items as well as other vertebrate and invertebrate material and vegetation. Dental enamel microwear of *D*. *vorax* favours this last explanation: analysed specimens show lower frequencies of pits than in other osteophages^42^ and are spread across the microwear space defined by extant mammals, from durophagous carnivores and omnivores, like the river otter *L. canadensis* and crab-eating raccoon *P. cancrivorus*, to small vertebrate and insect predators, like the ring-tailed cat *B. astutus*, and taxa that mainly feed on plants, like the kinkajou *Potos flavus* and muskrat *O. zibethicus* (Supplementary Fig. 14 and Supplementary Tables 12–15). These results perhaps also reflect that as the skull shape of *D. vorax* varied with ontogeny, as in extant marsupials^43^, so too did skull function and diet. Together, our data indicate that *D. vorax* was a powerful predator–scavenger in Late Cretaceous ecosystems with considerable faculties for cracking bone, invertebrate shell and/or turtle shell, but lacked some modifications of larger, more advanced osteophages, and *D. vorax* likely supplemented its diet of small vertebrates, carrion and/or molluscs with insects and plant matter.

Our study adds to a growing body of exceptional fossil discoveries^18^ and innovative quantitative analyses^31^,^44^ that has revealed an unexpectedly rich ecomorphological diversity of Mesozoic mammals. This recasting of mammals from the Age of Dinosaurs, however, has previously focused on ‘dead-end' non-therian lineages^18^. Until recently^30^, there has been little evidence that therians, which comprise 99.9% of all living mammals, underwent significant ecomorphological radiation during their more than 100 Myr of Mesozoic evolution. *D. vorax* highlights a previously under-appreciated radiation of Late Cretaceous NA stem marsupialiforms and its direct link to the origin and evolution of living marsupials.

## Methods

### Fossil specimens and their geologic age and context

The four specimens of *D. vorax* in this study are curated at three repositories: the North Dakota Geological Survey State Fossil Collection at the North Dakota Heritage Center State Museum in Bismarck, North Dakota, USA (NDGS); Sierra College Natural History Museum, Rocklin, California, USA (SCNHM); and University of Washington Burke Museum of Natural History and Culture, Seattle, Washington, USA (UWBM). The most complete specimens, NDGS 431 and UWBM 94084, form the primary basis for description of the skull morphology (Supplementary Note 1) and reconstruction (Fig. 2) of *D. vorax* with supplemental anatomical details incorporated from UWBM 94500 and SCNHM VMMa 20.

NDGS 431 (Supplementary Figs 1 and 2) is a partial cranium with parts of the zygomata, maxillae, palate and left P3–M4. The braincase, basicranium and petrosals are also preserved, although some aspects are crushed (Supplementary Figs 7–9). The semicircular canals of the inner ear are complete on the right side only, as revealed by computed tomography (CT) imaging (Supplementary Fig. 10). Found in close association with the partial cranium, and here considered part of the same individual, are fragmentary right and left premaxillae (lacking incisors), both canines, right P1–P3, left P1 and P2, and the right tympanic bulla and mastoid process. The P3 and M4 are fully erupted but have minimal dental wear, leading to the interpretation that NDGS 431 represents a young adult individual (Supplementary Note 1). A NDGS crew excavated NDGS 431 in 2007 in the upper third of the Hell Creek Formation at NDGS locality 27, 3.54 km northeast of Marmarth in Slope County, southwestern North Dakota. The specimen was found *in situ* within a yellow, cross-stratified silty, fine-grained sandstone unit, about 75 cm thick and discontinuous, pinching out laterally into a mudstone unit. This channel-lag deposit is 2 m above the base of a north-facing exposure of a small hill in the middle of a pasture, and is overlain and underlain by grey, popcorn-weathered mudstone. A typical latest Cretaceous (Lancian NA Land Mammal Age) vertebrate microfossil assemblage, including dinosaur teeth, was found as surface float and leaf fossils were found *in situ*.

UWBM 94084 (Supplementary Figs 3 and 4) is an incomplete rostrum with paired premaxillae and anterior parts of the maxillae and nasals. The left and right I1 and canines, the left P1–P3 and the right P1–2 are preserved. The relatively large size of the specimen and flat wear on the premolars suggest that the individual was a fully grown adult. A Rocky Mountain Dinosaur Resource Center crew found UWBM 94084 in 2002 during excavation of a hadrosaurid skeleton at Wayne's X Triceratops locality (UWBM locality C1526), 17.24 km southwest of Rhame in Bowman County, southwestern North Dakota. It is from the upper part of the Hell Creek Formation in a light grey to tan, fine-grained to medium-grained sandstone unit with haematitic and limonitic ironstone concretions, and represents a channel-lag deposit.

SCNHM VMMa 20 (Supplementary Figs 5a–d and 6a–d) is a fragment of a left anterior jugal and maxilla that includes an associated P1, P2–M2 and alveoli for M3 and M4. The dental wear is minor, suggesting that the specimen represents a young adult individual. A Sierra College Natural History Museum crew recovered SCNHM VMMa 20 in 2002 in the Bug Creek area, McCone County, northeastern Montana. The specimen was found as float but the local stratigraphy suggests that it was from the uppermost Hell Creek Formation.

UWBM 94500 (Supplementary Figs 5e–h and 6e–h) is a fragment of a left anterior jugal and maxilla with part of the palatal process, the P3–M2 and alveoli for M3–M4. The dental wear and size of the specimen indicate that it was from a fully grown adult individual, similar in size to the individual represented by UWBM 94084. A University of Washington crew excavated UWBM 94500 in 2012 in the uppermost Hell Creek Formation at the Second Level locality (UWBM locality C1692=University of California Museum of Paleontology locality V87101) in the McGuire Creek area, McCone County, northeastern Montana. The productive horizon is dark grey to brown, cross-stratified, fine-grained sandstone representing a Palaeocene channel-lag deposit ∼19 m below the MCZ coal with reworked latest Cretaceous fossils, including dinosaur teeth and mostly likely UWBM 94500 (ref. 45).

### Micro-CT scanning and processing

The main part of NDGS 431 was scanned at Pennsylvania State University. Resolution of each CT slice was 1,024 × 1,024 pixels, the interpixel spacing was 0.0672, mm and interslice spacing was 0.0673, mm. The associated parts of NDGS 431 were scanned at the University of Washington (UW) Santana Lab. Resolution of each CT slice was 1,024 × 1,304 pixels, and pixel size was 0.0296, mm. UWBM 94084 was scanned at the SANTA Facility of the UW School of Medicine. Resolution of each CT slice was 668 × 1,968 pixels, and pixel size was 0.0344, mm. UWBM 94500 and SCNHM VMMa 20 were scanned at the Duke University MICRO-CT Facility at 0.0205, mm resolution. CT imagery of *Didelphodon* was compared with that of the extant didelphids *D. virginiana* and *Chironectes minimus* that were scanned at the University of Texas at Austin CT Facility (www.digimorph.org/specimens).

A composite model of the cranium was assembled in Geomagic Studio 2014.1.0 (3D Systems, Rock Hill, South Carolina, USA). Digital isolation of the petrosals, segmentation of the bony labyrinth and measurements of segmented digital endocasts were performed in Avizo 7.0 (VSG Imaging) following previously used methods^46^,^47^,^48^. Orthogonal planes through the cranium were defined following methods modified from Rodgers^49^. Points along the medial sutures in the posterior half of the cranium were used to define the vertical sagittal plane (*XZ*). A modified Ried's line from the centre of the ventral rim of the infraorbital foramen to the centre of the dorsal rim of the external auditory meatus was used to define the horizontal (frontal) plane (*XY*). Normally, the inferior rim of the orbit is used, but neither orbit is preserved on the specimen. The ventral rim of the infraorbital foramen is preserved on the left side of the cranium only, but the dorsal rims of both external auditory meati are present. The axial plane (*YZ*) intersects the centres of the dorsal rims of the external auditory meati and is perpendicular to the sagittal and horizontal planes.

### Phylogenetic analysis

We used a data matrix of 164 characters (67 dental, 86 cranial and 11 mandibular) that were scored for 48 taxa (13 outgroup taxa, 10 deltatheroidans and 25 marsupialiforms). The data matrix was mainly derived from Rougier *et al*.^2^,^10^,^50^ but with modifications and additions from other previous studies^9^,^11^,^51^,^52^ and this study. On the basis of the new craniodental material, we updated the scores of *Didelphodon* for 41 characters in the matrix; 34 were previously unknown (?) and seven were known but revised with new information. *Didelphodon* is now the best-known Cretaceous marsupialiform in the data set (88% of characters scored versus 72% in Bi *et al*.^9^). To this data matrix, we also added two NA Cenozoic marsupialiforms (*Herpetotherium* and *Mimoperadectes* informed by *Peradectes*) that had previously been included in other data matrices^11^,^20^ but not in the matrices of Rougier *et al*.^2^,^10^,^50^ or its derivatives^9^,^52^. Scores for *Herpetotherium* and *Mimoperadectes*–*Peradectes* were largely based on previous studies^11^,^20^ and our own observations. A few scores for *Pucadelphys*, *Mayulestes*, the ‘Gurlin Tsav skull' and *Andinodelphys* were modified from Rougier *et al*.^10^ on the basis of Horovitz *et al*.^11^ and our own observations. We used Appendix B of Horovitz and Sánchez-Villagra^53^ for character correspondence across studies. The character list and scores for *Didelphodon* are in Supplementary Note 2. We submitted our data matrix (Supplementary Data 1) to a new technology search in TNT (version 1.1)^54^, using the sectorial, ratchet, drift and tree-fusing strategies with 500 minimum length trees^55^,^56^ and designating 11 of the multi-state characters as additive.

### Testing alternative tree topologies

To test whether the topology of our strict consensus tree is robust, we compared it with two alternative topologies. Previous analyses have generally placed NA Cretaceous stem marsupialiforms as an early diverging marsupialiform group or as a grade distant from the crown group, and SA stem marsupialiforms have been placed as sister taxon to Marsupialia^2^,^9^,^10^,^50^,^52^ or to Herpetotheriidae+Marsupialia^11^,^20^. To compare these alternatives with our strict consensus tree, we conducted Templeton tests in TNT (Supplementary Note 4).

### Constraining the timing of the origin of Marsupialia

Marsupialia is a crown clade formed by the most recent common ancestor of extant metatherians, plus all of its descendants^2^. According to our phylogenetic analysis, *Mimoperadectes*–*Peradectes* and *Herpetotherium* are included along with extant marsupials (*Dromiciops*, *Didelphis* and *Marmosa*) in Marsupialia. The origin of crown group marsupials is thereby constrained by the age of the oldest fossils attributed to Marsupialia: the oldest known species of *Peradectes* (for example, *P. minor*) are from the early Palaeocene (middle Puercan, ca. 65.1 Myr ago)^32^, whereas the oldest known species of *Herpetotherium* (for example, *H. comstocki*) are from the earliest Eocene (early Wasatchian, ca. 55 Myr ago). Because *Herpetotherium* is a member of the monophyletic Herpetotheriidae^6^,^57^, we can further extend its lineage back to at least the oldest member of this clade. Williamson and Lofgren^58^ placed *Golerdelphys stocki*, which is known by four isolated molars, in the Herpetotheriidae. These specimens are from the middle Tiffanian-age Goler Formation in California (middle Palaeocene, ca. 60 Myr ago). In addition, Case *et al*.^12^ proposed the late Maastrichtian (Lancian) *Nortedelphys* as a herpetotheriid, but phylogenetic analyses have not supported this claim^6^,^57^. The V-shaped upper-molar centrocrista shared by *Nortedelphys* and herpetotheriids appears to have convergently evolved in these taxa^57^. In recent phylogenetic analyses by Williamson *et al*.^6^,^57^, the late Campanian (Judithian, ca. 79–74 Myr ago) taxon *Ectocentrocristus foxi* falls among the herpetotheriids. However, because this taxon is based on a single tooth that some have argued is not a molar but a deciduous fourth premolar of *Turgidodon*^6^, we cautiously interpret these data: the oldest herpetotheriids went back to at least the middle Palaeocene (Fig. 3, grey star), as represented by *Golerdelphys*, and possibly the late Campanian (Fig. 3, grey star with query), as represented by *Ectocentrocristus*. Thus, according to our results, the origin of crown marsupials extends back to at least the early Palaeocene (age of *Peradectes minor*) and possibly the late Campanian (age of *E. foxi*).

If additional sampling of the Australidelphia leads to a revised phylogenetic placement of *Dromiciops* such that *Mimoperadectes*–*Peradectes* and *Herpetotherium* are sister taxa to rather than members of Marsupialia, then the origin of crown marsupials would be early Eocene (54–52 Myr ago)^33^ based on the oldest undisputed crown marsupial fossils from the Itaborai local fauna of Brazil^59^. In turn, the origin of stem marsupials would be constrained by the age of the ghost lineage implied by the age of the oldest member of the sister taxon, the Herpetotheriidae or Peradectidae (early Palaeocene or possibly late Campanian).

A last alternative to consider is that some have argued, on the basis of dental similarity, that *Glasbius* from the late Maastrichtian (Lancian) of NA is sister taxon to the SA Polydolopimorphia^60^, which in turn has been allied with either the extant Caenolestidae or the extant Microbiotheria^53^,^61^. If correct, this would expand the membership of Marsupialia and push back its origins well into the Cretaceous (100 Myr ago or older). However, we remain unconvinced of this hypothesis because it has not been rigorously tested in a phylogenetic analysis that utilizes a broad sample of taxa and anatomical characters. Instead, we follow Clemens^16^ in arguing that the dental similarities of these taxa are likely the result of convergent evolution toward a frugivorous/granivorous diet, not shared recent ancestry.

### Body mass estimates

Numerous predictive body mass equations have been developed from regression analyses of craniodental measurements of extant mammals^22^,^62^. To estimate body mass in *D. vorax*, we chose to use formulae developed from an all-dasyuromorphian data set^22^. We deemed this data set as most appropriate for the task because dasyuromorphians are a restricted taxonomic group of marsupials and they adequately approximate the craniodental morphology of *Didelphodon*. This equation also provided reasonable body mass estimates for a sample of *D. virginiana* with tag mass data (data not presented here). We estimated body masses of four specimens of *D. vorax*: (1) the relatively complete skull of a young adult (NDGS 431); the snout of a more mature individual (UWBM 94084); the near-complete dentary of young adult (UWBM 102139); and the more fragmentary dentary of a young adult (UCMP 159909). For NDGS 431, we used the formulae that use TSL and upper-molar-occlusal-row length (UMORL). For UWBM 94084, we estimated TSL based on the scaling relationship between length of the snout tip to P3–M1 embrasure and TSL in NDGS 431. For the dentaries, we used the formulae for lower-molar-row length and lower-molar-occlusal-row length. These body mass estimates are shown in Supplementary Table 4. We used the body mass estimate for NDGS 431 from the UMORL because it has a lower prediction error (14%) than that for TSL (19%). UMORL measurements are not available for UWBM 94084, so we used the body mass estimate from the scaled TSL.

### Estimate of maximum prey size of *D. vorax*

Predator body size constrains the maximum body size of prey that it can kill. For energetic and biomechanical reasons, small- to medium-sized predators (<15–20 kg) most commonly feed on small vertebrates and invertebrates that are only a small fraction of their own body mass^36^,^63^,^64^. Meers^23^ developed a regression formula to predict maximum prey size from a predator's body mass, using a large data set of extant predators and their largest known prey items. To correct for logarithmic transformation bias^65^, Meers^23^ calculated a Ratio estimator (RE) correction factor according to the method of Snowdon^66^. Because that RE correction factor was not reported, we reconstructed it from the regression formula and the appendix of comparative data in Meers^23^. We applied Meers' formula and the reconstructed RE correction factor to four different body mass estimates of *D. vorax* (Supplementary Table 4). The estimates and 95% confidence intervals are presented in Supplementary Table 5.

### Bite force analysis

To estimate bite force in *D. vorax* and other taxa, we replicated Thomason's method using two-dimensional images of digital surface models instead of photographs of dry skulls^67^,^68^. In this analysis, we used the composite model of the CT scanned *D. vorax* cranial material. Stereolithography files of skulls of *L. canadensis* (UCLA 15275), *S. harrisii* (USNM 307639), *T. taxus* (LACM 45012) and *D. virginiana* (TMM M-2517) were downloaded from the DigiMorph database. The left side of the composite skull model of *D. vorax* NDGS 431 is the most complete, so for all taxa we took measurements of the left side of the skull and doubled the estimated bite force to account for bilateral biting forces.

The variables required to estimate bite force from linear measurements of the skull include the following: the estimated physiological cross-sectional area (PCSA) of the muscle groups involved in jaw adduction; the length of the lever arms of each muscle force vector about the temporal–mandibular joint (TMJ), and the length of the outlever; or distance from the TMJ axis to the bite point. Our bite force calculations are based on the contributions of the temporalis muscle and the combined masseter and pterygoid muscles. To estimate temporalis PCSA, the skulls were digitally positioned in Geomagic Studio using four points on the skull to define the plane of posterodorsal view: the most posterior points of the left and right zygomatic arches, and the most lateral points of the left and right postorbital processes^68^. The combined masseter and pterygoid area was estimated by positioning the skulls in ventral view. The temporalis area was outlined as the left infratemporal fossa bounded by the zygomatic arch and brain case in posterodorsal view (Supplementary Fig. 11a), and the masseter and ptergyoid area was outlined as the infratemporal fossa bounded by the zygomatic arch and brain case in ventral view (Supplementary Fig. 11b)^67^. The area of the estimated muscle cross-sections and the area centroids were calculated in ImageJ v1.49 (U.S. National Institutes of Health, Bethesda, Maryland, USA).

Although many values of muscle stress have been used in the literature, we used a constant muscle stress value of 300 kPa (ref. 24) multiplied by the estimated PCSA to calculate the force of the temporalis, *T*, and the masseter and pterygoid, *M*. Muscle force was then applied to the skull as a single force vector acting perpendicular to the plane of the muscle cross-sectional area, through the area centroid (Supplementary Fig. 11c). The lever arm of the masseter, *m*, was measured as the perpendicular distance from the area centroid to the TMJ in ventral view (Supplementary Fig. 11b) in ImageJ. The temporalis centroid and plane of estimated PCSA were transferred to the skull in lateral view, and the temporalis lever arm, *t*, was measured as the perpendicular distance from centroid to the TMJ (Supplementary Fig. 12c). The out-lever length *o* was measured as the lateral distance from the TMJ to two bite points, one at the tip of the canine and one at the centre of the first molar. Bite force was then calculated from the sum of the moments generated by the temporalis and masseter forces about the TMJ divided by the distance to the bite point, which was then doubled to account for bilateral biting^67^:


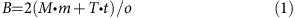


Our measurements and bite force estimates are presented in Supplementary Table 6. We could only estimate bite forces for *D. vorax* using the smaller, more complete skull (NDGS 431); however, we would predict larger bite force estimates for the larger UWBM 94084. In light of the nearly identical canine bite force estimates for *S. harrisii* by us and by Wroe *et al*.^24^, using different specimens, we have confidence that our results for the other taxa are consistent with those presented in Table 1 of Wroe *et al*.^24^ Bite force scales with body mass^23^,^24^, such that comparison of bite force estimates across a wide range of body sizes is problematic. To adjust for this body size allometry and to enable comparisons of relative bite forces, Wroe *et al*.^24^ estimated BFQ, which utilizes the residuals of the regression of log body mass and log estimated bite force. Supplementary Table 7 contains the body mass and canine bite force data from Table 1 of Wroe *et al*.^24^ and this study. From that data set, we performed a regression of log body mass versus log CBs. The regression formula was then used to calculate new BFQ values for all taxa. Some BFQs differ slightly from those in Table 1 of Wroe *et al*.^24^ due to the data set-specific regression formulae. For *D. vorax*, we used the maximum body mass estimate for NDGS 431 (Supplementary Table 4) as a conservative estimate of BFQ (lower body mass estimates produce higher BFQs).

### Relative premolar size

Van Valkenburgh^25^ and Hartstone-Rose^26^ discriminated among dietary categories in carnivorans and marsupials using a number of cranial and dental indices. In particular, they found that RPS was greatest among durophagous taxa compared with all other dietary categories. RPS was calculated for *D. vorax* as described in Appendix 2 of Van Valkenburgh^25^: the maximum width of the ultimate premolar (in mm) was divided by the cube root of body mass (in kg).

### Canine bending strength analysis

Van Valkenburgh and Ruff^28^ investigated the biomechanical link between canine tooth shape and feeding behaviour in large carnivores by modelling the canine tooth as a solid beam, and, in turn, estimating its resistance to bending forces in the anteroposterior and mediolateral directions. We applied those methods to specimens of *D. vorax*, *D. virginiana*, *S. harrisii* and *T. taxus* (Supplementary Table 9). All measurements were taken with digital calipers on actual specimens as shown in Fig. 1 of Van Valkenburgh and Ruff^28^ and according to the methods in their text. We then added our data to their data set and made bivariate plots to examine the canine shape of *D. vorax* in the broader context of extant mammalian predators (Supplementary Fig. 12 and Supplementary Table 10).

### Mandibular bending strength analysis

Therrien^29^ investigated the biomechanical link between mandibular shape and feeding behaviour in extant carnivorans by modelling the mandibular body as a solid beam, and, in turn, estimating its resistance to bending forces in the mediolateral and the dorsoventral planes. Given the same material properties of the bone, a dorsoventrally deep mandibular body is better able to withstand forces in the dorsoventral plane, whereas a mediolaterally wide mandibular body is better able to withstand forces in the mediolateral plane. We applied these methods to specimens of *D. vorax*, *D. virginiana* and *S. harrisii* (Supplementary Table 11). All measurements were taken with digital calipers on actual specimens as shown in Fig. 2 of Therrien^29^ and according to the methods in that text. Because metatherians differ from extant carnivorans in dental formula, we took measurements at different interdental gaps: canine; p2–p3; p3–m1; m1–m2; m2–3; and post m4. Cross-sectional dimensions and, in turn, bending strengths can vary along the mandibular body in a way that correlates with feeding behaviours in extant carnivorans^29^. For comparison with taxa in Figs 3–7 of Therrien^29^, we plotted our data as mandibular force profiles in Supplementary Fig. 13.

### Low-magnification dental microwear analysis

Dental microwear is used as a proxy to infer feeding behaviour of individual animals. It is quantified by counting pits and scratches on the enamel of the occlusal surface of the teeth and has been widely used to study the diet of a variety of mammals^69^,^70^. These features reflect the material properties of the food and exogenous items eaten^71^. Specifically, a high density of scratches suggests the ingestion of abrasive foods (including plant material and grit), whereas abundant pits indicate a diet composed of hard foods from both plant (for example, seeds and nuts) and animal sources (for example, bones and shells)^72^,^73^. By quantifying the microwear of species with known diets, a comparative data set can be built and then used to estimate the diet of taxa for which food intake is unknown, including extinct species. Although multiple studies have quantified the microwear of numerous extant mammals whose diet is known, analyses of inter-observer error demonstrate that each investigation of the diet of fossil taxa should develop its own comparative data set^74^.

We therefore quantified microwear in 16 modern mammals. We focused our sample on small mammals with body masses comparable to those of Mesozoic taxa in general, and *Didelphodon* in particular, including numerous marsupials. Their diets range from carnivory to herbivory, including omnivores, insectivores and animals capable of durophagy (Supplementary Table 12). Diets of extant mammals were taken from the literature. On the basis of the dominant food items consumed by the animal, we categorized each taxon in one of five dietary categories: carnivory; animal-dominated omnivory; omnivory; plant-dominated omnivory; and herbivory.

All specimens sampled were adult individuals from museum collections collected in the wild (the diet of a captive animal is not representative of the wild behaviour of that animal). Because the microwear features counted by different observers cannot be compared, only one researcher (J.J.C.) counted pits and scratches on the tooth surface of the specimens sampled. We used low-magnification stereomicroscopy to quantify microwear in our sample. Specimens were observed under a Leica MZ 9.5 microscope with a × 2.0 objective. Microwear features were counted using a reticle to count a consistent area of the tooth across specimens (0.3 mm^2^) at × 70 magnification. Because of its enamel properties, tooth enamel cannot be directly observed under the stereomicroscope^69^. Therefore, we moulded specimens and made clear, high-definition epoxy casts of the tooth of interest following the method of Solounias and Semprebon^69^. Following previous studies, we counted microwear on the upper and lower second molars of eutherian mammals^75^, except carnivorans for which we counted the first upper or lower molar^76^. We counted the analogous teeth, the upper and lower third molars, of metatherians. Microwear was counted on the crushing surface of the tooth: the protocone of the upper molars, or the talonid basin of the lower molars. On each tooth, nine different features were quantified (Supplementary Tables 14 and 15): four different types of pits (small pits, large pits, small puncture pits and large puncture pits) and five different types of scratches (cross scratches, fine scratches, coarse scratches, hypercoarse scratches and gouges). We excluded from our data set fossil specimens with taphonomic damage^77^.

We assessed intra-observer error by counting a subset of 20 specimens, including a carnivore (*L. canadensis*), two animal-dominated omnivores (*Nasua narica* and *T. glis*) and a plant-dominated omnivores (*Potorous trydactylus*), twice and comparing the two independent counts (Supplementary Table 13). The intra-observer consistency in microwear counts was calculated using Spearman's rank-order correlation coefficient, which is not as sensitive to outliers and non-normal distributions as other coefficients. Because most of the pits are small pits and most of the scratches are cross scratches, we also ran correlation analyses for these two categories of microwear features. The number of microwear features counted for each specimen was normalized by the total number of microwear features counted for the specimen to account for differences in the number of microwear features across specimens. The percentage of each category of microwear features for each specimen was used in a multivariate analysis of the microwear signature (that is, combined percentages of the different categories of microwear features). Because of the non-normal and nonlinear nature of our data, which include many zeros, we used PCoA to construct a multivariate microwear space to visualize similarities between the microwear signature of *D. vorax* and that of extant mammals (Supplementary Fig. 14). The analysis of microwear features was run in R 3.1.3 (ref. 78) using RStudio 0.98.1103 (ref. 79) and the package vegan 2.3–4 (ref. 80).

### Data availability

The data sets generated or analysed during the current study are available from the corresponding author on reasonable request.

## Additional information

**How to cite this article**: Wilson, G. P. *et al*. A large carnivorous mammal from the Late Cretaceous and the North American origin of marsupials. *Nat. Commun.* **7**, 13734 doi: 10.1038/ncomms13734 (2016).

**Publisher's note:** Springer Nature remains neutral with regard to jurisdictional claims in published maps and institutional affiliations.

## Supplementary Material

Supplementary Information Supplementary Figures, Supplementary Tables, Supplementary Notes and Supplementary References

Supplementary Data 1 Phylogenetic data matrix

Supplementary Data 2 Metatherian synapomorphies
