# Supplementary material for: A large carnivorous mammal from the Late Cretaceous and the North American origin of marsupials
Source: Nat Commun. 2016 Dec 8;7:13734. doi: 10.1038/ncomms13734 (PMC5155139; doi:10.1038/ncomms13734)
Supplement: Supplementary Information — Supplementary Figures, Supplementary Tables, Supplementary Notes and Supplementary References [file ncomms13734-s1.pdf]

Supplementary Figures

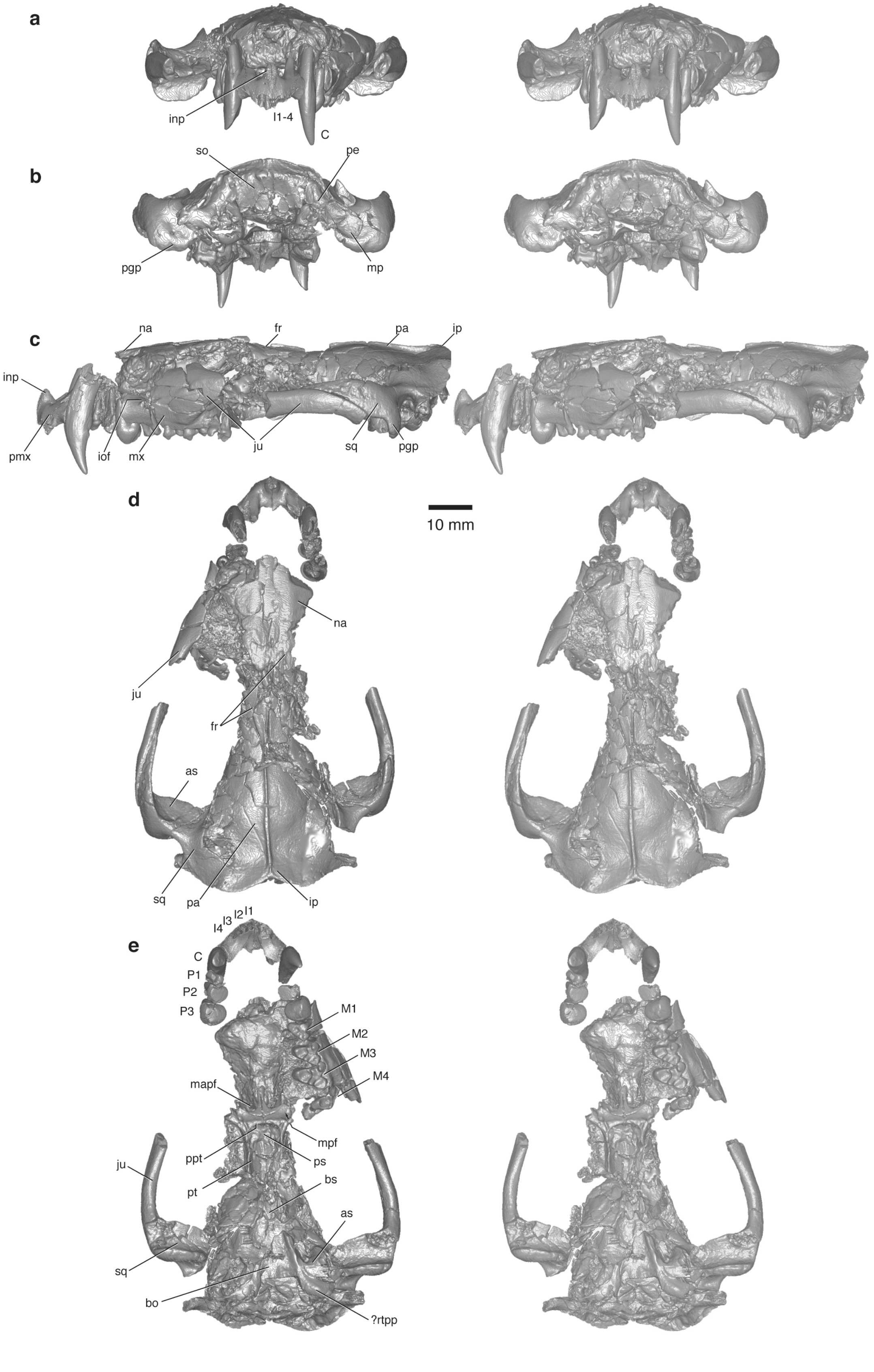

Supplementary Figure 1. Skull of the Late Cretaceous marsupialiform *Didelphodon vorax*. Stereopairs of NDGS 431 in anterior (**a**), posterior (**b**), left lateral (**c**), dorsal (**d**), and ventral (**e**) views. All images from digitally rendered, micro-computed tomography scans. Abbreviations: as, alisphenoid; bo, basioccipital; bs, basisphenoid; C, upper canine; I1–4, upper incisors 1–4; inp, internarial process; inf, incisive foramen; iof, infraorbital foramen; ip, interparietal; ju, jugal; M1–4, upper molars 1–4; mapf, major palatine fenestra; mp, mastoid process; mpf, minor palatine foramen; mx, maxilla; na, nasal; P1–3, upper premolars 1–3; pa, parietal; pe, petrosal; pgp, postglenoid process; pmx, premaxilla; ppt, postpalatine torus; ps, presphenoid; pt, pterygoid; ?rtpp, rostral tympanic process of the petrosal; so, supraoccipital; sq, squamosal. Scale bar equals 10 mm.

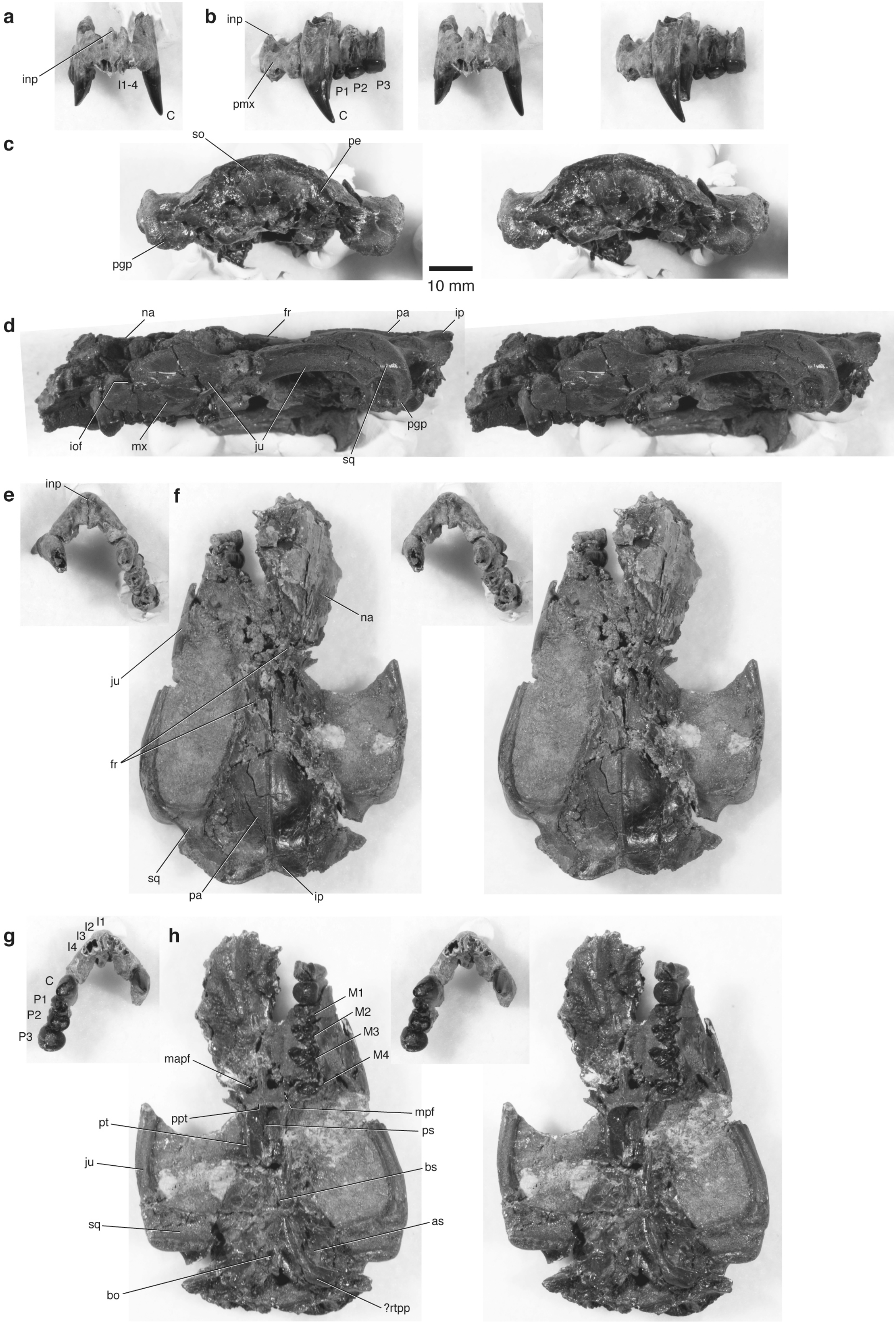
a
inp
I1-4
C
b
inp
pmx
P1
P2
P3
C
c
so
pe
pgp
10 mm
d
na
fr
pa
ip
iof
mx
ju
sq
pgp
e
inp
f
na
ju
fr
sq
pa
ip
g
I1
I2
I3
I4
C
P1
P2
P3
h
M1
M2
M3
M4
mapf
mpf
ppt
pt
ps
ju
bs
sq
as
bo
?rtpp

Supplementary Figure 2. Skull of the Late Cretaceous marsupialiform *Didelphodon vorax*. Stereophotographs of NDGS 431 premaxillae in anterior (**a**), left lateral (**b**), ventral (**e**), and dorsal (**g**) views, and main skull in posterior (**c**), left lateral (**d**), dorsal (**f**), and ventral (**h**) views. Abbreviations as in Supplementary Figure 1. Scale bar equals 10 mm.

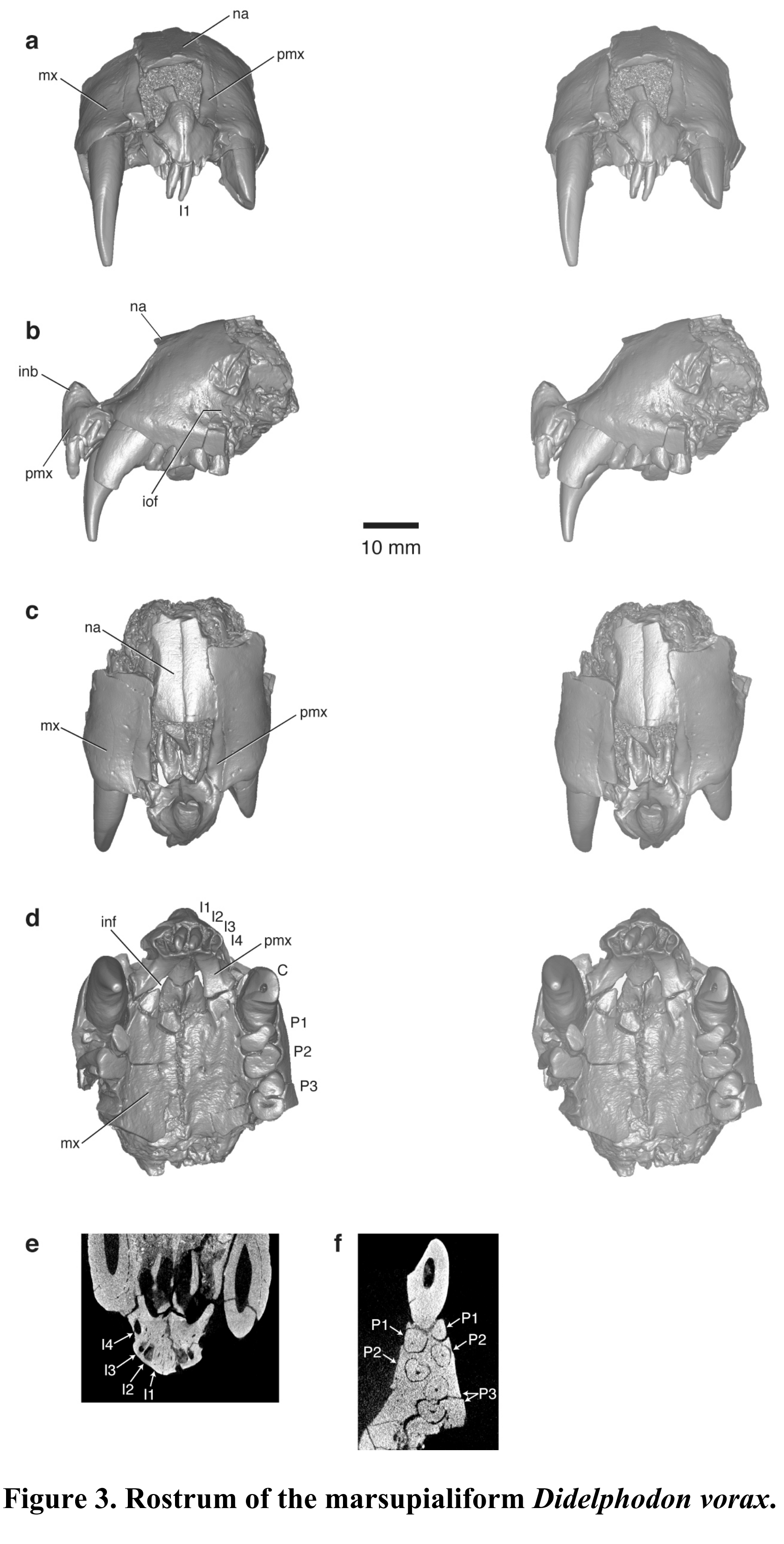

Supplementary Figure 3. Rostrum of the marsupialiform *Didelphodon vorax*. Stereopairs of UWBM 94084 in anterior (**a**), left lateral (**b**), dorsal (**c**), and ventral (**d**) views; and transverse x-

ray sections of the anterior rostrum with canine and incisor alveoli (**e**) and the premolar row alveoli (**f**). All images from digitally rendered, micro-computed tomography scans. Abbreviations as in Supplementary Figure 1. Scale bar equals 10 mm.

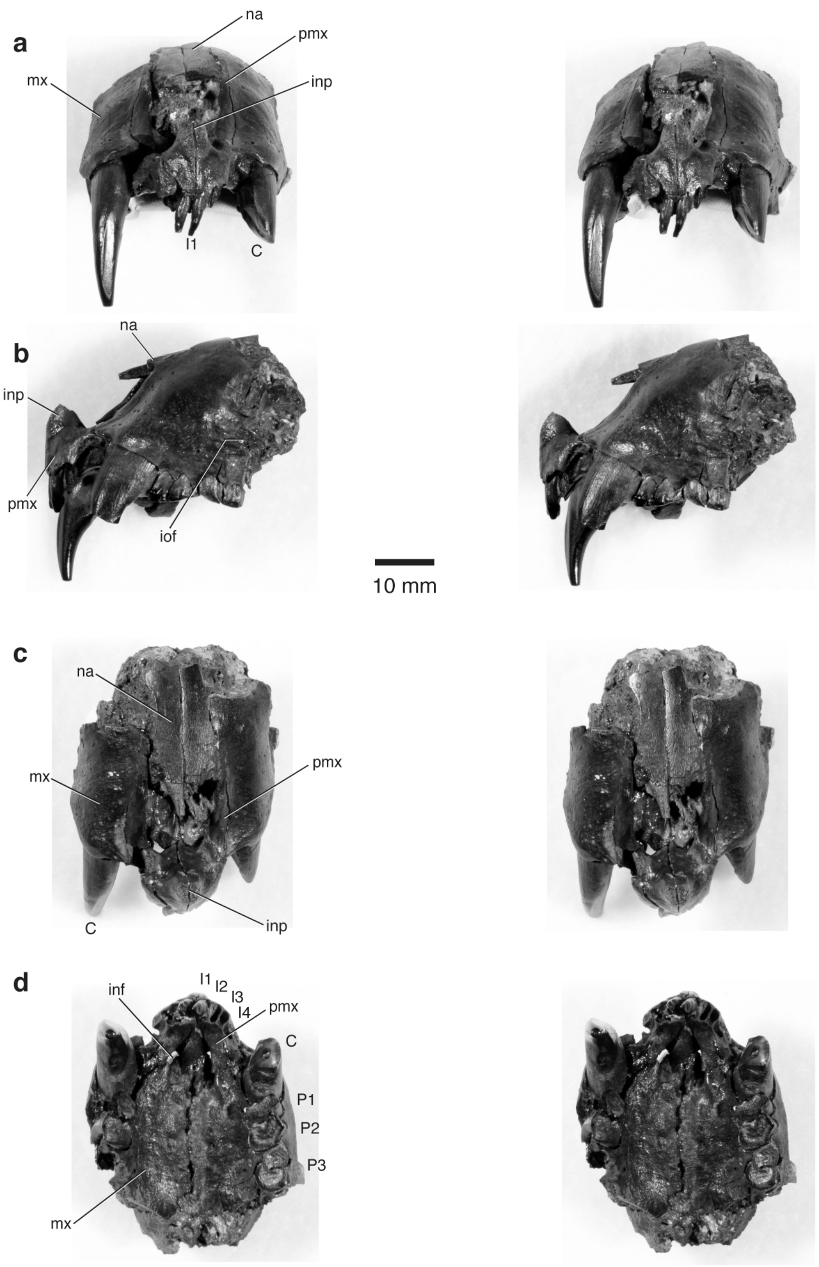

Supplementary Figure 4. Rostrum of the marsupialiform *Didelphodon vorax*. Stereophotographs of UWBM 94084 in anterior (**a**), left lateral (**b**), dorsal (**c**), and ventral (**d**) views. Abbreviations as in Supplementary Figure 1. Scale bar equals 10 mm.

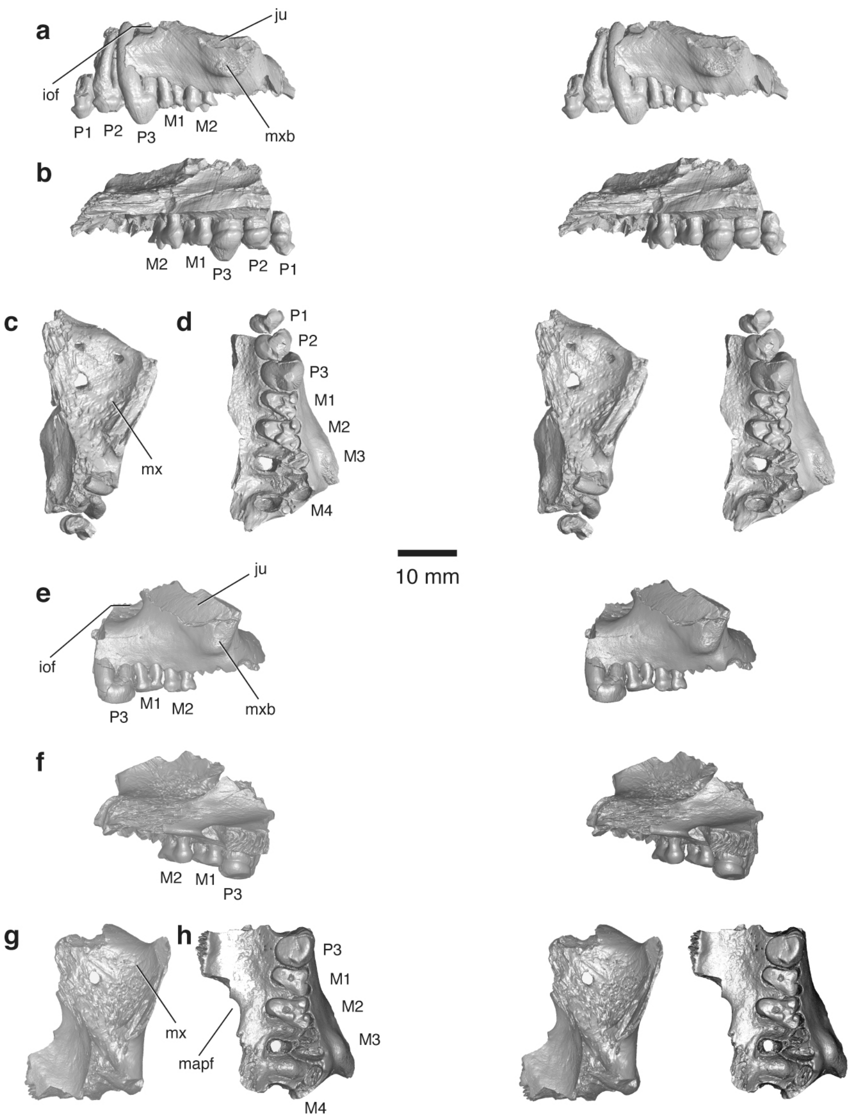

Supplementary Figure 5. Maxillae and jugals of the marsupialiform *Didelphodon vorax*. Stereopairs of SCNHM VMMa 20, partial left maxillae and jugal, in lateral (**a**), medial (**b**), dorsal (**c**), and ventral (**d**) views; and stereopairs of UWBM 94500, partial left maxilla and jugal, in lateral (**e**), medial (**f**), dorsal (**g**), and ventral (**h**) views. All images from digitally rendered,

micro-computed tomography scans. Abbreviations as in Supplementary Figure 1. Scale bar equals 10 mm.

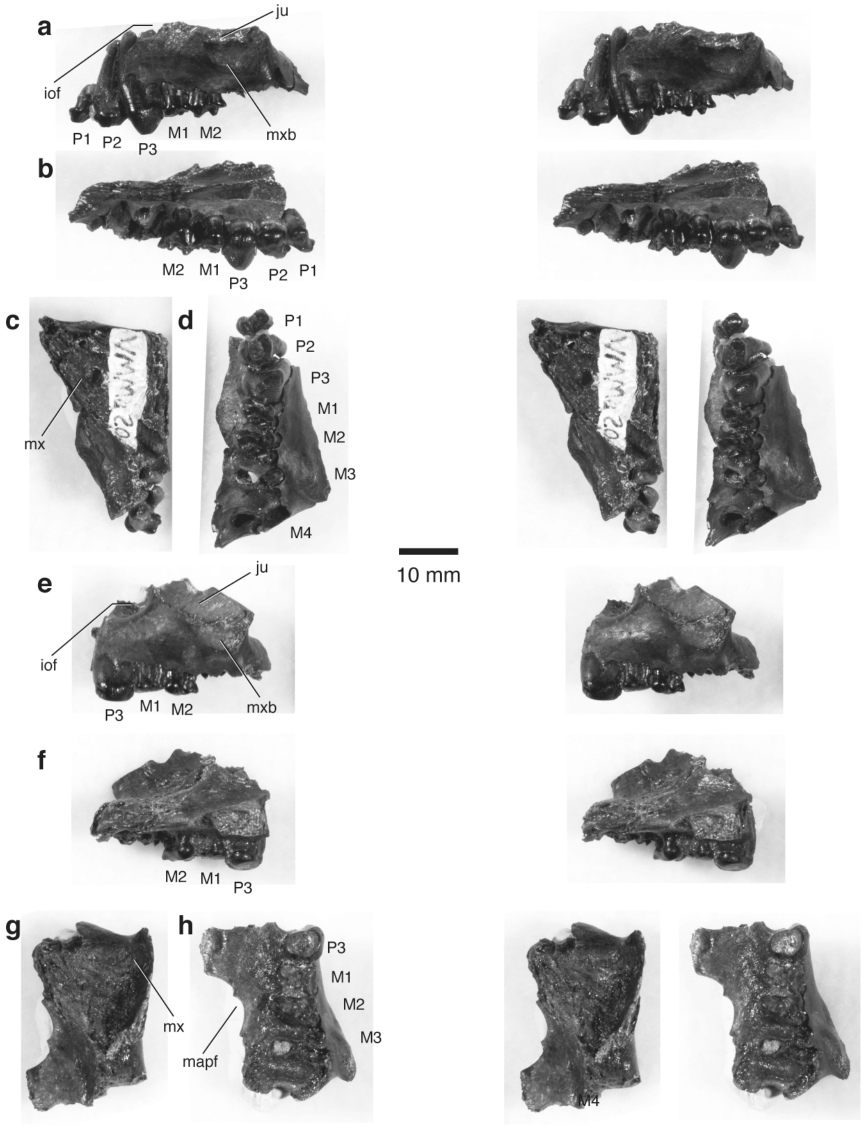

Supplementary Figure 6. Maxillae and jugals of the marsupialiform *Didelphodon vorax*. Stereophotographs of SCNHM VMMa 20, partial left maxilla and jugal, in lateral (**a**), medial (**b**), dorsal (**c**), and ventral (**d**) views; and stereophotographs of UWBM 94500, partial left maxilla and jugal, in lateral (**e**), medial (**f**), dorsal (**g**), and ventral (**h**) views. Abbreviations as in Supplementary Figure 1. Scale bar equals 10 mm.

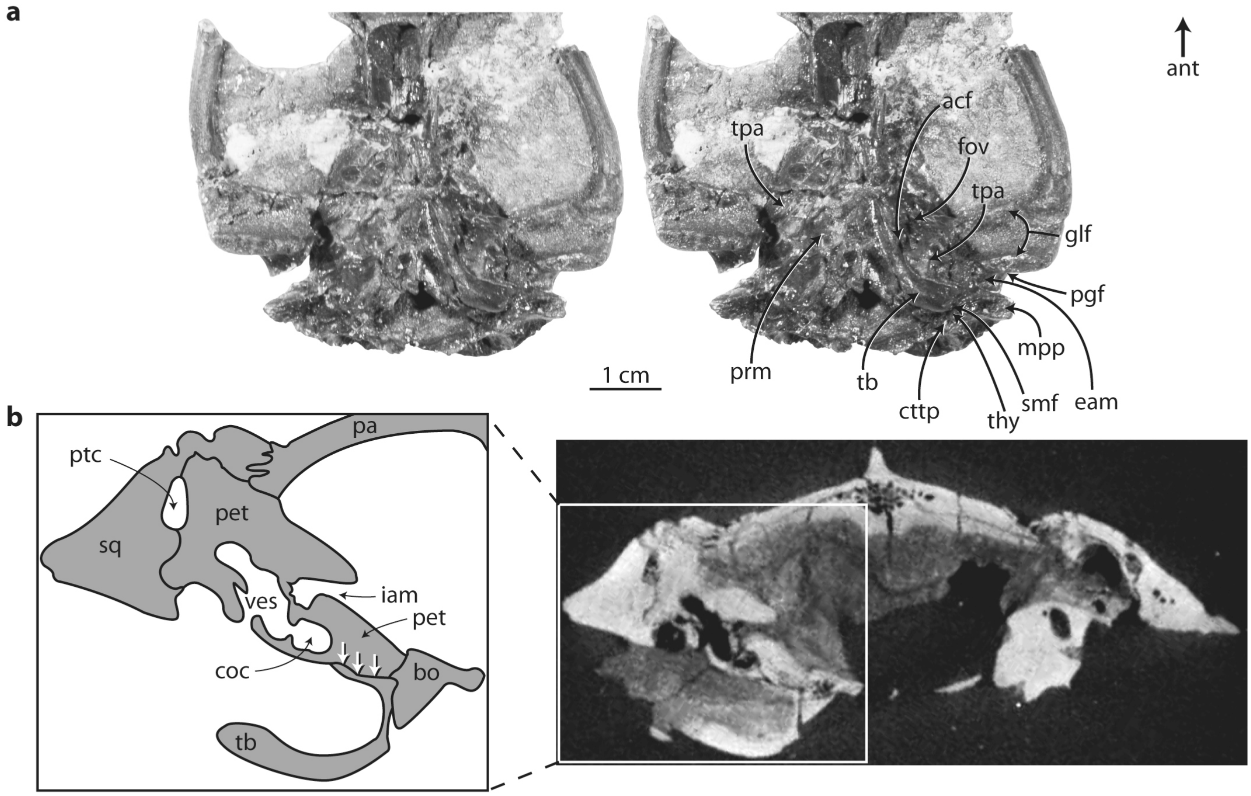

Supplementary Figure 7. Left tympanic bulla of the marsupialiform *Didelphodon vorax*. Stereophotographs (**a**) and labeled line drawing (**b**) of left ear region of NDGS 431 in ventral view. Gray areas in line drawing indicate damaged regions. Original CT slice through skull and line drawing of left ear region. White arrows indicate potential fracture through the ear region. Abbreviations: acf, anterior carotid foramen; ant, anterior direction; bo, basioccipital; bs, basisphenoid; coc, cochlea; cttp, caudal tympanic process of petrosal; eam, external acoustic meatus completed by petrosal and squamosal; fov, foramen ovale; glf, glenoid fossa of squamosal; iam, internal acoustic meatus; lat, lateral direction; mpp, mastoid process of petrosal; pa, parietal; pet, petrosal; pgf, postglenod foramen; ptc, posttemporal canal; smf, stylomastoid foramen; sq, squamosal; tb, tympanic bulla (of petrosal?); thy, tympanohyal; tpa, tympanic process of alisphenoid; ves, vestibule. Scale bar equals 1 cm.

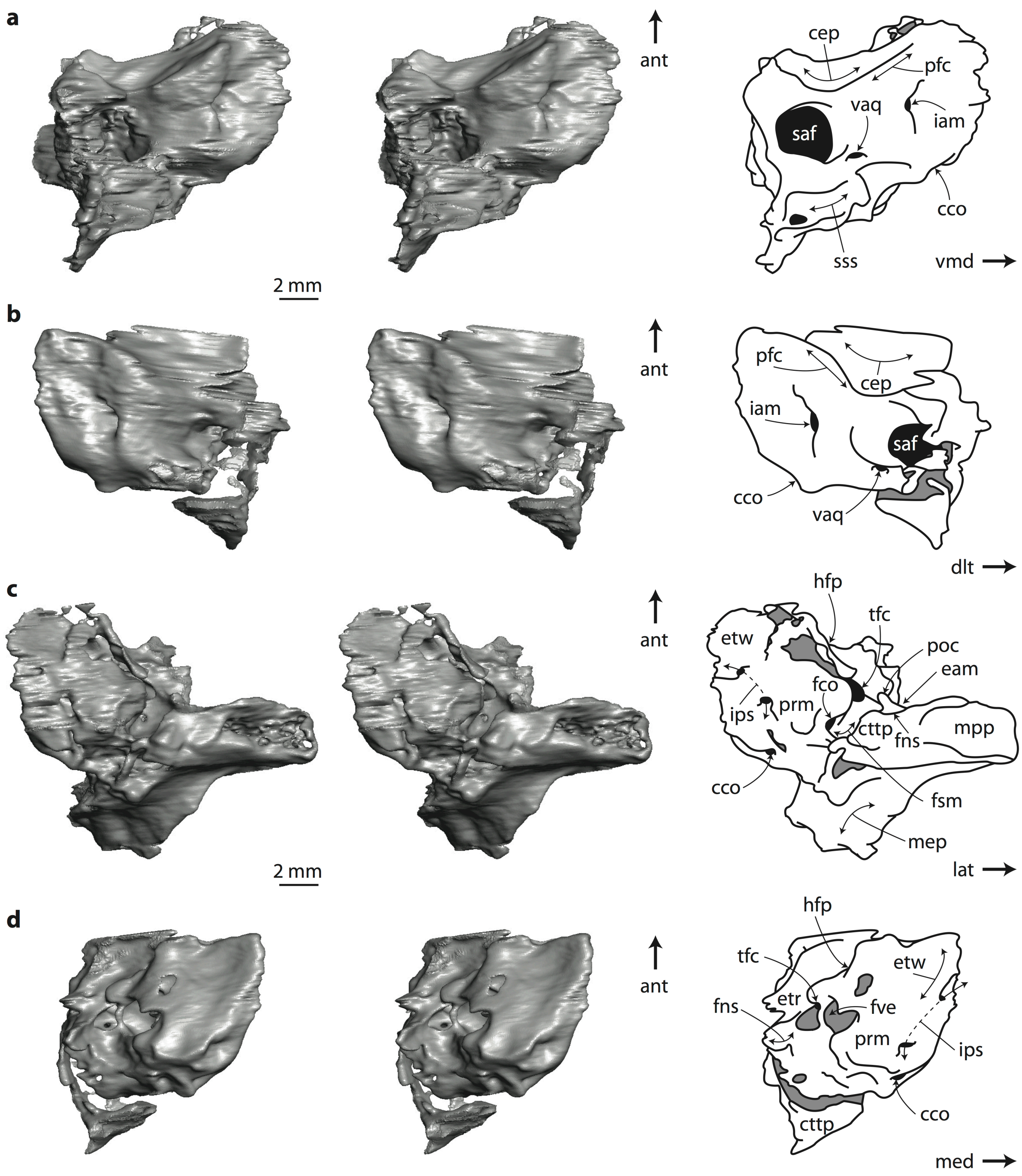

Supplementary Figure 8. Petrosal bones of the marsupialiform *Didelphodon vorax*.

Stereopairs rendered from micro-computed tomography scans and labeled line drawings of left (**a**, **c**) and right (**b**, **d**) petrosal bones of NDGS 431, in ventral (**a**, **b**) and dorsomedial views (**c**, **d**). Gray areas in line drawings indicate damaged regions. Abbreviations: ant, anterior direction; cco,

opening for bony canaliculus cochleae; cep, petrosal contribution to cavum epiptericum; cttp, caudal tympanic process of petrosal; dlt, dorsolateral direction; eam, petrosal contribution to external acoustic meatus; etr, epitympanic recess; etw, epitympanic wing of petrosal; fco, fenestra cochleae; fns, facial nerve sulcus; fsm, fossa for stapedius muscle; fve, fenestra vestibuli; hfp, hiatus Fallopii for greater petrosal nerve; iam, internal acoustic meatus; ips, sulcus and canal for inferior petrosal sinus; lat, lateral direction; med, medial direction; mep, mastoid exposure of petrosal; mpp, mastoid process of petrosal; pfc, prefacial commissure; poc, foramen for prootic canal; prm, promontorium; saf, subarcuate fossa; sss, sulcus for sigmoid sinus; tfc, tympanic aperture of facial canal; vaq, opening for bony channel of vestibular aqueduct; vmd, ventromedial direction. Scale bar equals 2 mm.

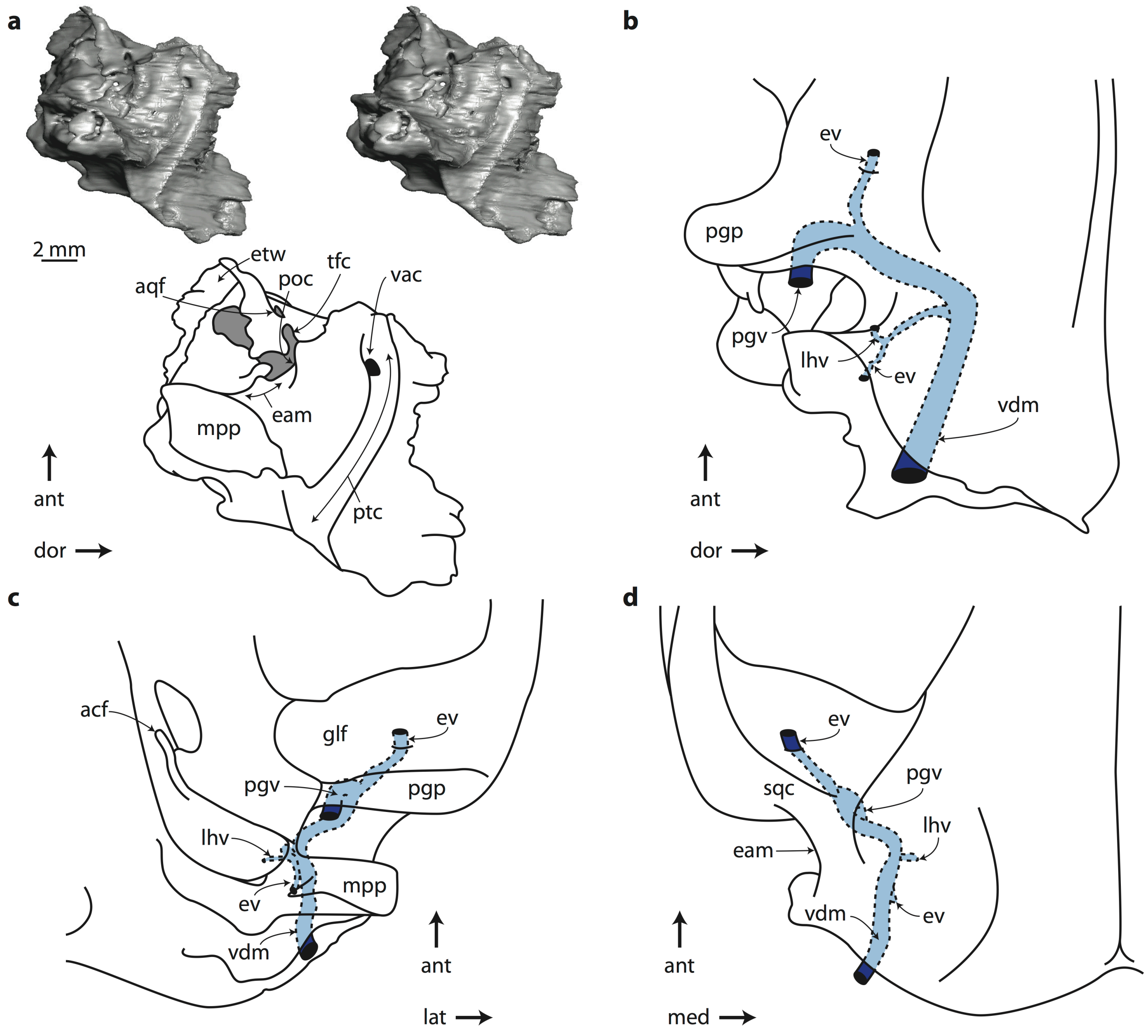

Supplementary Figure 9. Posttemporal vasculature of *Didelphodon vorax*. Stereopairs of the left petrosal bone rendered from micro-computed tomography scans and labeled line drawing of NDGS 431, in lateral view (**a**). Gray areas in line drawings indicate damaged regions. Venous reconstructions of posttemporal venous system in lateral (**b**), ventral (**c**), and dorsal (**d**) views. Abbreviations: acf, anterior carotid foramen; ant, anterior direction; aqf, aqueductus Fallopii; dor, dorsal direction; eam, petrosal contribution to external acoustic meatus; etw, epitympanic wing of petrosal; ev, emissary vein; glf, glenoid fossa; lat, laterl direction; lhv, lateral head vein; med, medial direction; mpp, mastoid process of petrosal; pgp, postglenoid process; pgv, postglenoid

vein; poc, foramen for prootic canal; ptc, petrosal contribution of posttemporal canal; sqc, squamosal crest; tfc, tympanic opening of facial canal; vac, vascular canal; vdm, vena diploetica magna. Scale bar equals 2 mm.

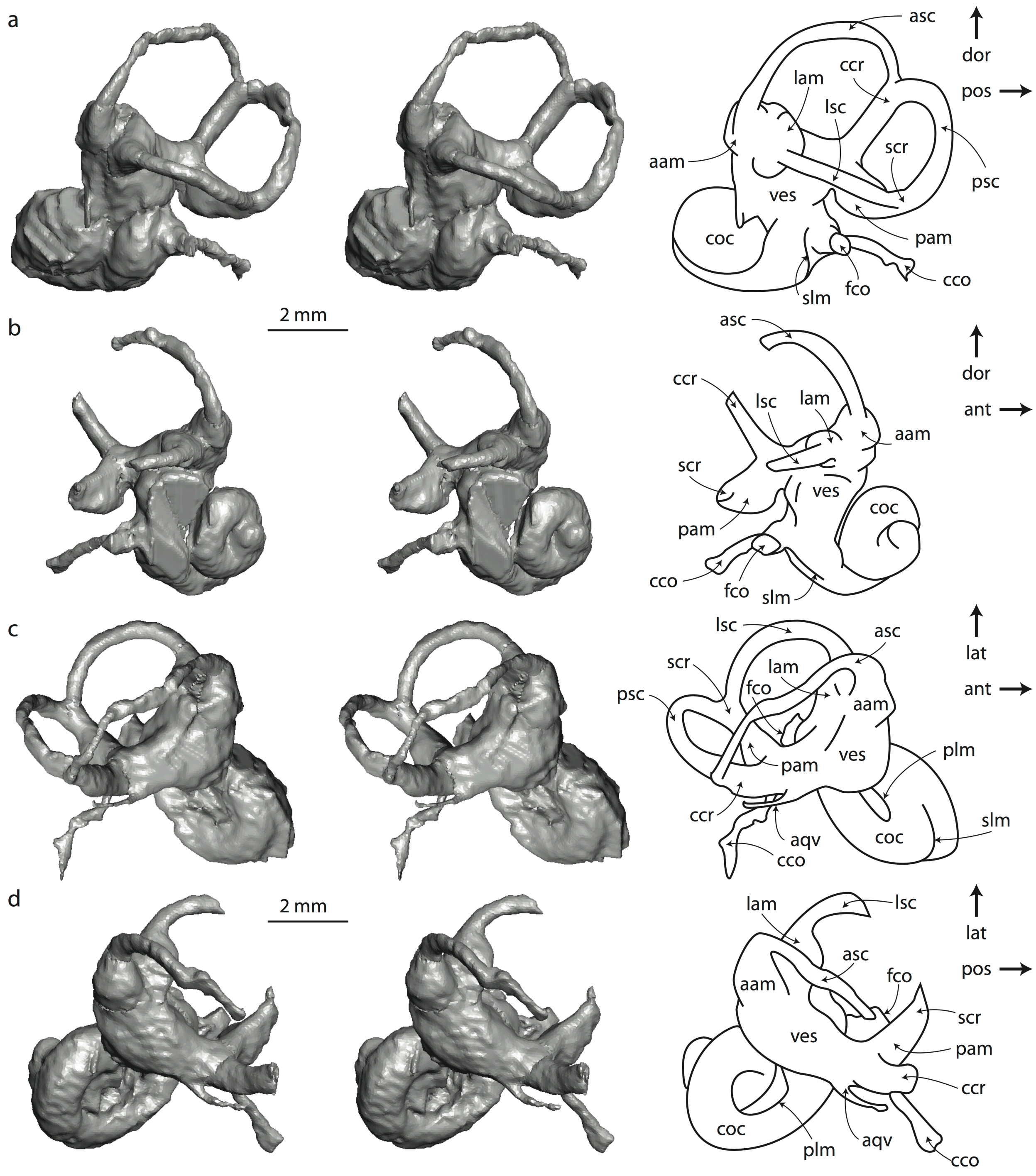

Supplementary Figure 10. Bony labyrinth endocasts of the inner ear of *Didelphodon vorax*. Stereopairs rendered from micro-computed tomography scans and labeled line drawings of NDGS 431, left (**a**, **c**) and right (**b**, **d**), in lateral (**a**, **b**) and dorsal views (**c**, **d**). Abbreviations: aam, anterior ampulla; ant, anterior direction; asc, anterior semicircular canal; aqv, bony channel of vestibular aqueduct; cco, bony canaliculus cochleae; ccr, common crus; coc, cochlea; dor, dorsal; fco, fenestra cochleae; lam, lateral ampulla; lat, lateral direction; lsc, lateral semicircular canal; pam, posterior ampulla; plm, bony primary lamina; pos, posterior direction; psc, posterior semicircular canal; scr, secondary common crus; slm, bony secondary lamina; ves, vestibule. Scale bar equals 2 mm.

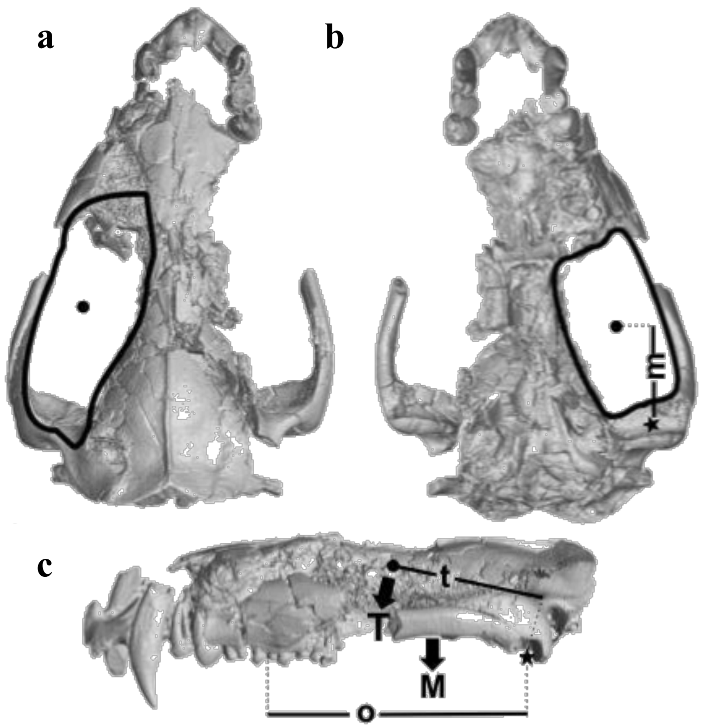

Supplementary Figure 11. **Schematic of method of bite-force calculations.** Bite force was calculated from 2D images of skull surface models as shown here in *Didelphodon vorax*. Outline and centroid of temporalis muscle area estimated in posteriodorsal view (**a**). The muscle force

vector **T** acts perpendicular to the plane of the cross-sectional area through the centroid. Outline of combined masseter and pterygoid muscles in ventral view (**b**). The muscle force vector **M** acts through the centroid (dot) and has a lever arm **m** that acts about the TMJ axis (star). The TMJ and the area centroid of the estimated temporalis area are transferred to the lateral view (**c**) in order to calculate the temporalis lever arm, **t**. The out-lever length, **o**, is measured as the distance from the TMJ to the tip of the canine and the center of the first molar in lateral view.

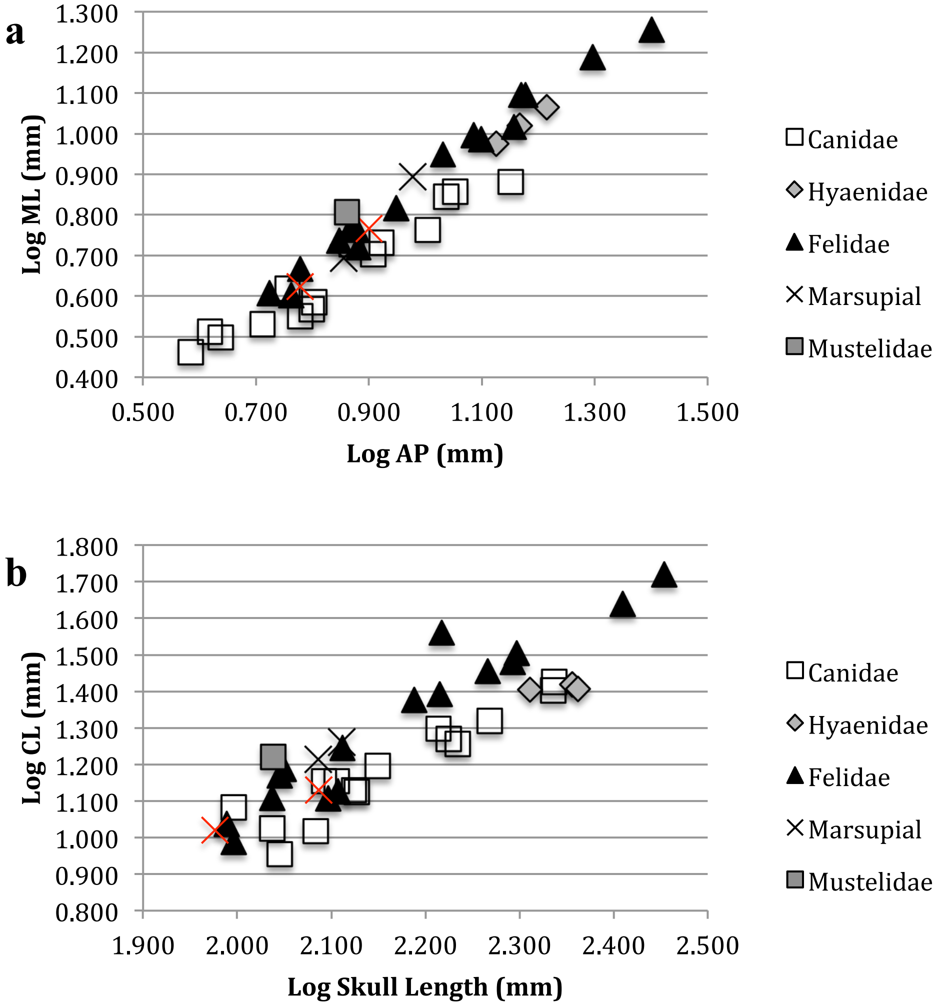

c

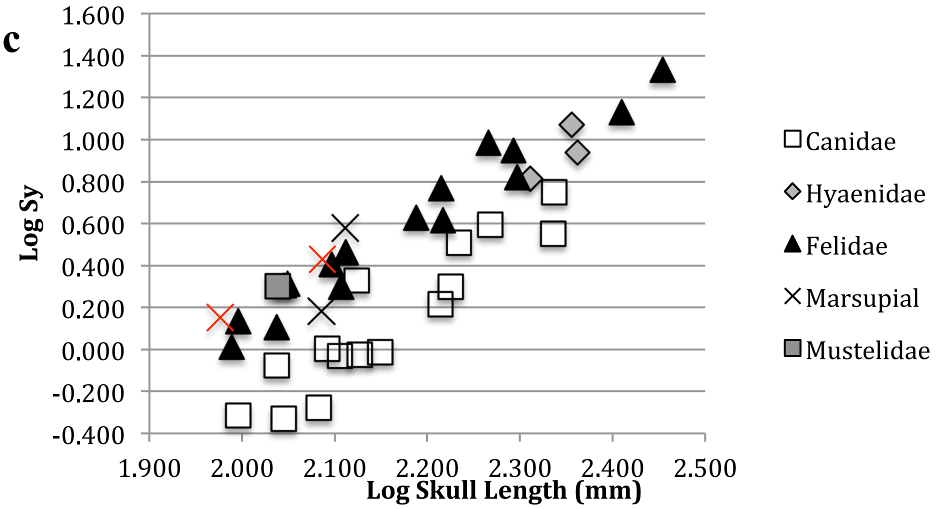

d

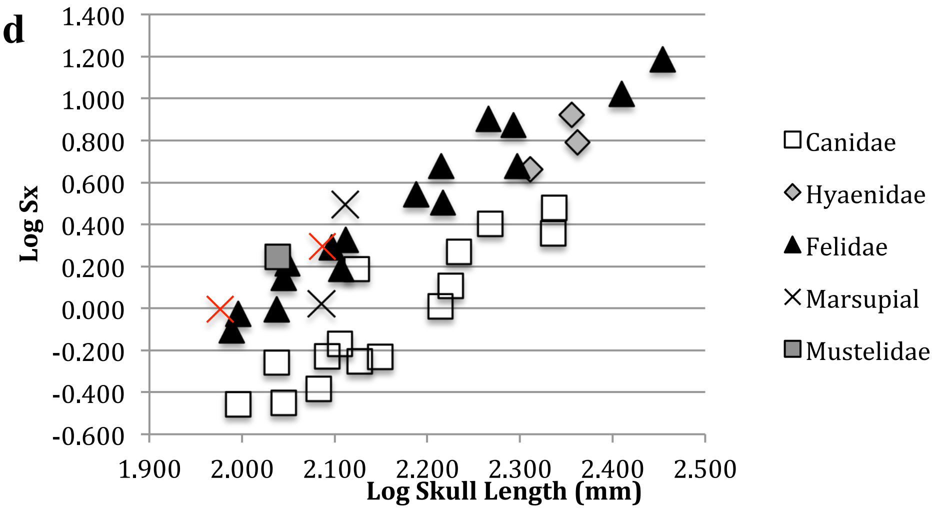

Supplementary Figure 12. Plots of canine bending strength. Bivariate log-log plots of anteroposterior radius vs. mediolateral radius of canine (**a**); total skull length vs. canine length (**b**); total skull length vs. bending strength (**c**) about the mediolateral axis, Sy; and total skull length vs. bending strength (**d**) about the anteroposterior axis, Sx. Data are from Supplementary Table 10. N = 39 specimens. Red markers indicate specimens of *Didelphodon vorax*.

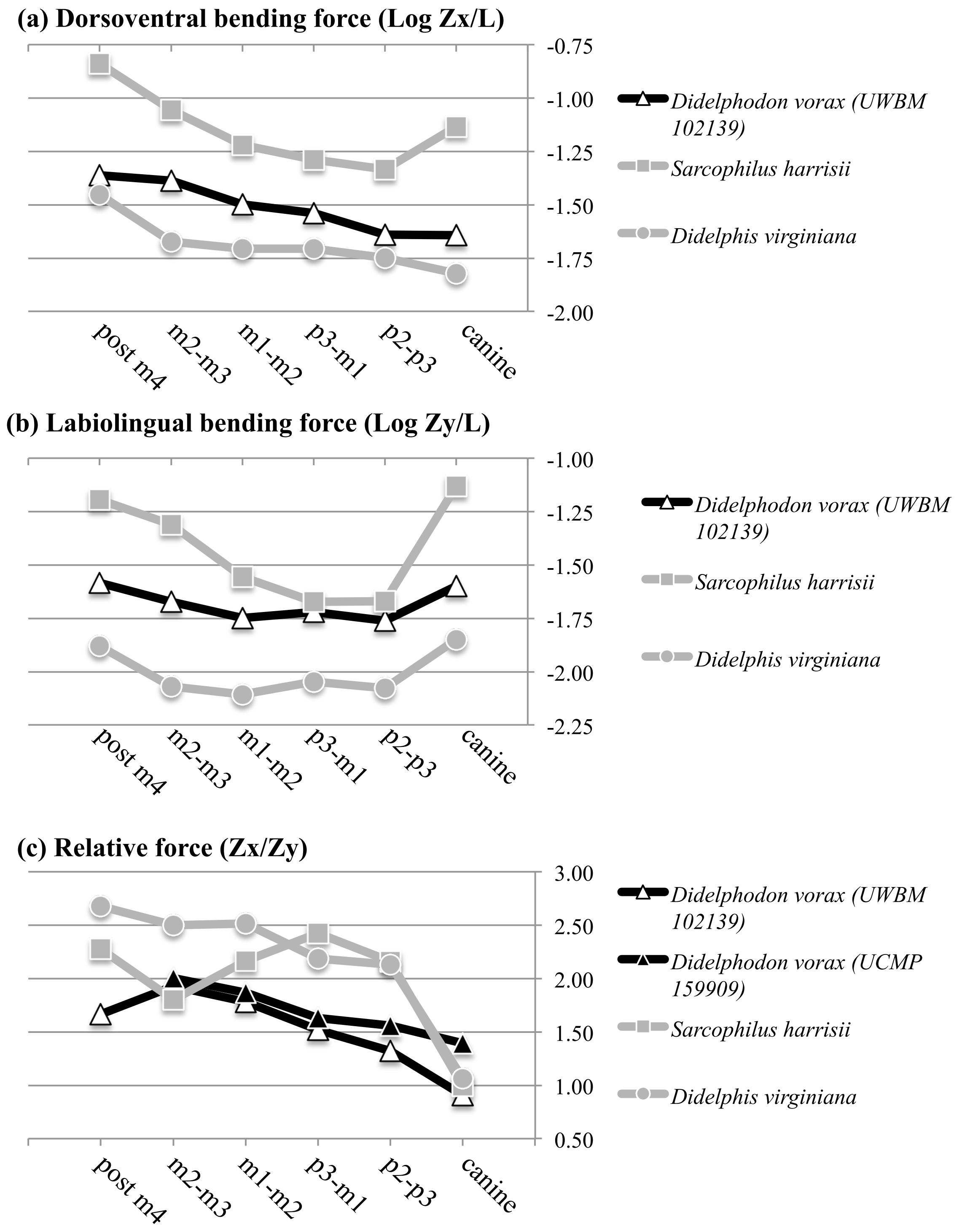

Supplementary Figure 13. Mandibular bending-strength profiles of *Didelphodon vorax.*

Profiles of dorsoventral (**a**), labiolingual (**b**), and relative force (**c**) bending strength of

Didelphodon vorax compared to those of *Sarcophilus harrisii* and *Didelphis virginiana*. N = 3–4 specimens. Data are from Supplementary Table 11.

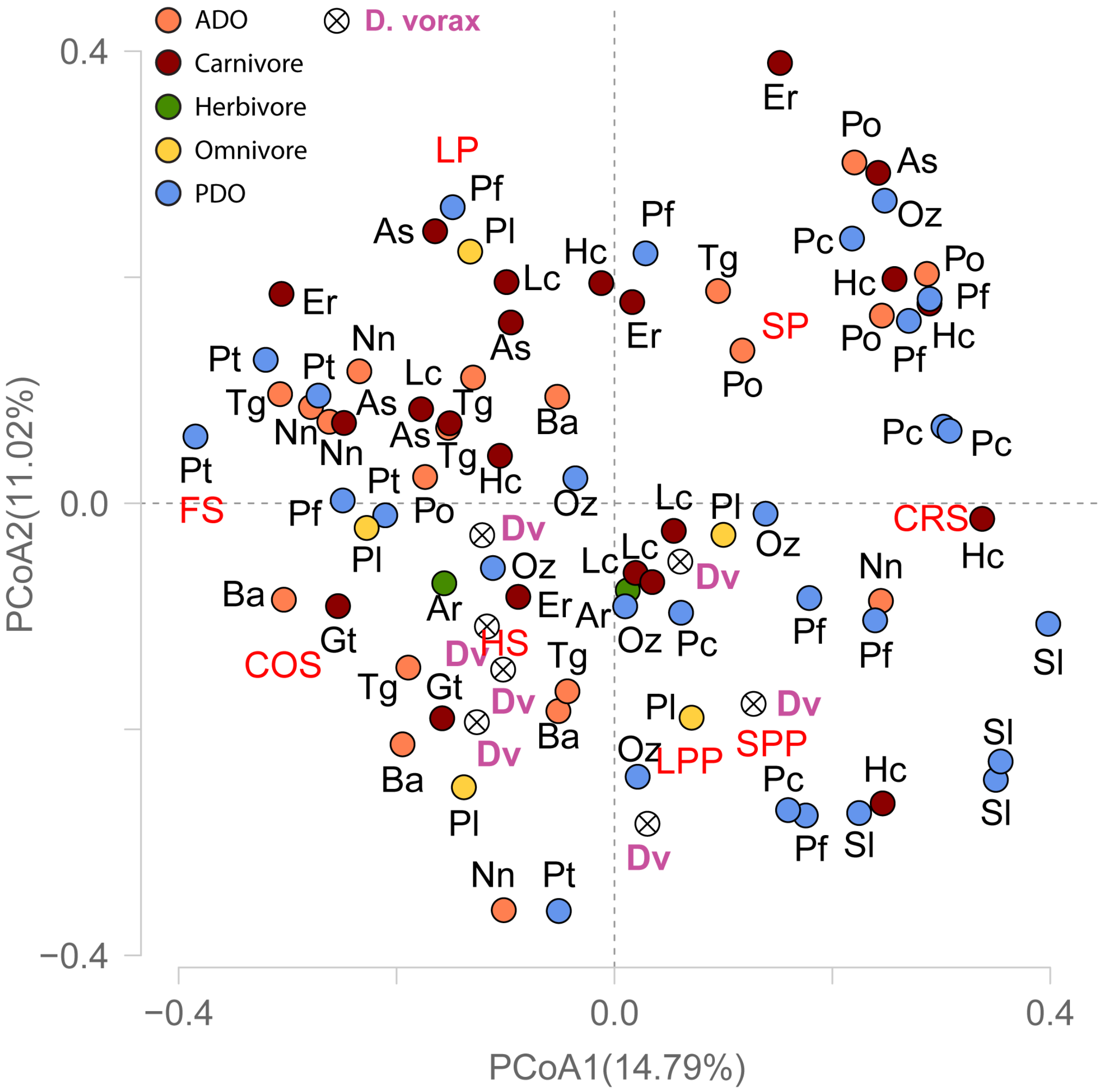

Supplementary Figure 14. Low-magnification dental microwear of *Didelphodon vorax*. Plot of the Principal Coordinate Analysis (PCoA) of microwear data of extant mammals and *Didelphodon vorax*. N = 7 specimens of *Didelphodon vorax*, N = 77 specimens of 16 extant species. Abbreviations for taxon names (in black and purple) and features (in red) are provided in Supplementary Table 14.

Supplementary Tables

Supplementary Table 1. Dimensions of the bony labyrinth of *Didelphodon vorax* (NDGS 431).

	Left	Right*
Volume measurements (in mm^3)		
Bony labyrinth	25.96	21.21
Cochlea	11.96	11.25
Vestibule	8.33	-
Common crus	0.8	-
Anterior ampulla	1.01	0.81
Lateral ampulla	0.88	0.83
Posterior ampulla†	1.22	-
Anterior canal	0.49	-
Lateral canal	0.55	-
Posterior canal	0.71	-
Coiling of cochlea (in °)	646	-
Linear measurements (in mm)		
Bony labyrinth length	7.1	-
Cochlea height	2.1	2
Cochlea width	4.96	3.54
Cochlea length	-	7.56
Anterior canal length	8.35	-
Anterior canal arc height	2.88	3.12
Anterior canal arc width	4.05	4.44
Anterior canal arc radius	1.89	2.07
Anterior canal lumen diameter	0.39	-
Lateral canal length	6.29	-
Lateral canal arc height	2.53	-
Lateral canal arc width	3.03	-
Lateral canal arc radius	1.39	-
Lateral canal lumen diameter	0.42	-
Posterior canal length	6.69	-
Posterior canal arc height	2.9	-
Posterior canal arc width	2.94	-
Posterior canal arc radius	1.45	-
Posterior canal lumen diameter	0.32	-

*Not every measurement could be taken for the right bony labyrinth on account of poor preservation. The apex of the left cochlea could not be determined in order to measure canal length and degree of coiling.
†Volume for posterior ampulla includes secondary common crus.

Supplementary Table 2. Angular orientations of semicircular canal planes of *Didelphodon vorax* (NDGS 431). (Most angles could not be measured for the right side on account of incomplete preservation.)

Ipsilateral canals (same side)	
left anterior vs. left lateral	80.5°
left anterior vs. left posterior	90.6°
left lateral vs. left posterior	90.4°
Contralateral canals (opposite sides)	
right anterior vs. left anterior	82.3°
Synergistic canals	
left posterior vs. right anterior	6.4°
Deviation from orthogonal planes	
right anterior vs. sagittal	43.4°
left anterior vs. sagittal	40.7°
left lateral vs. sagittal	92.2°
left posterior vs. sagittal	47.9°
right anterior vs. horizontal	76.9°
left anterior vs. horizontal	75.4°
left lateral vs. horizontal	20.3°
left posterior vs. horizontal	70.1°
right anterior vs. axial	49.1°
left anterior vs. axial	53.9°
left lateral vs. axial	69.2°
left posterior vs. axial	48.7°

Supplementary Table 3. Dental measurements for specimens of *Didelphodon vorax*. L, length; W, width; MW, mesial width; DW, distal width; OR, observed range; AVG, average; SD, standard deviation; CV, coefficient of variation. All measurements in mm.

		NDGS 431°		UWBM 94084°		SCNHM VMMa 20	UWBM 94500	OR	AVG	SD	CV
		left	right	left	right	left	right				
P1	L	–	3.82	4.63	–	4.61	–	3.82–4.63	4.35	0.46	10.6
	W	–	3.94	5.41	–	5.71	–	3.94–5.71	5.02	0.95	18.9
P2	L	5.02	5.04	5.50	5.33	5.90	–	5.02–5.90	5.22	0.43	8.3
	W	3.94*	5.10	5.97	5.85	6.17	–	5.10–6.17	5.73	0.56	9.7
P3	L	5.78	5.93	6.98**	–	6.39	6.59	5.78–6.59	6.28	0.38	6.1
	W	6.39	6.30	6.01	–	7.32	6.51	6.01–7.32	6.55	0.56	8.5
M1	L	4.32	–	–	–	4.86	4.70	4.32–4.86	4.63	0.28	6.0
	MW	4.79	–	–	–	5.90	5.92	4.79–5.92	5.54	0.65	11.8
	DW	5.64	–	–	–	6.65	6.68	5.64–6.68	6.32	0.59	9.4
M2	L	5.31	–	–	–	6.07	5.43	5.31–6.07	5.60	0.41	7.4
	MW	6.05	–	–	–	7.21	7.09	6.05–7.21	6.78	0.64	9.4
	DW	6.83	–	–	–	8.05	8.03	6.83–8.05	7.64	0.70	9.1
M3	L	6.33	–	–	–	–	–	–	–	–	–
	MW	7.35	–	–	–	–	–	–	–	–	–
	DW	8.38	–	–	–	–	–	–	–	–	–
M4	L	5.72	–	–	–	–	–	–	–	–	–
	MW	6.99	–	–	–	–	–	–	–	–	–
	DW	5.64	–	–	–	–	–	–	–	–	–

*minimum estimate, due to breakage; not included in summary statistics

**maximum estimate, due to separation from fracture; not included in summary statistics

°measurements from left and right sides were averaged for summary statistics

Supplementary Table 4. Body-mass estimates for *Didelphodon vorax* (in kg) based on craniodental measurements (in mm). Equations are from Myers[1] for dasyuromorphians. LMORL, lower molar occlusal row length; LMRL, lower molar row length; PE, prediction error; TSL, total skull length; UMORL, upper molar occlusal row length. Estimates in boldface are used in the main text. UWBM 94084 TSL scaled based on comparisons with NDGS 431.

Measurements (mm)	NDGS 431	UWBM 94084	UCMP 159909	UWBM 102139
TSL	94.7	122.1	–	–
UMORL	21.7	–	–	–
LMORL	–	–	23.3	23.7
LMRL	–	–	23.3	23.7
Body mass estimates (kg)				
TSL mass	2.2	**5.2**	–	–
TSL mass - PE	1.8	4.2	–	–
TSL mass + PE	2.6	6.2	–	–
UMORL mass	**2.4**	–	–	–
UMORL mass - PE	2.1	–	–	–
UMORL mass + PE	2.7	–	–	–
LMRL mass	–	–	2.1	2.2
LMRL mass - PE	–	–	1.8	2.0
LMRL mass + PE	–	–	2.4	2.5
LMORL mass	–	–	2.2	2.4
LMORL mass - PE	–	–	1.9	2.0
LMORL mass + PE	–	–	2.6	2.8

Supplementary Table 5. Maximum prey size estimates for specimens of *Didelphodon vorax*, using the regression formula and 95% confidence intervals of Meers[2] for "All predators": ln max prey size = 1.1523 * ln body mass – 0.8263. Ratio Estimator correction factor = 1.701.

Specimen	body mass (kg)	ln body mass	estimated prey mass (kg)	corrected prey mass (kg)	lower 95% CI	upper 95% CI
NDGS 431	2.4	0.88	1.2	2.0	1.0	4.1
NDGS 431-min. est.	2.1	0.74	1.0	1.8	0.9	3.4
UWBM 94084	5.2	1.65	2.9	5.0	2.2	11.4
UWBM 94084-max. est.	6.2	1.82	3.6	6.1	2.6	14.5

Supplementary Table 6. Measurements and bite force estimates from the dry-skull method of Thomason[3].

Species	Temporalis area (cm^2)	Masseter area (cm^2)	Temporalis moment (cm)	Masseter moment (cm)	Muscle stress (N/cm^2)	Temporalis Force (N)	Masseter Force (N)	M1 outlever (cm)	P3 outlever (cm)	Canine outlever (cm)	M1 bite force bilateral (N)	P3 bite force bilateral (N)	Canine bite force bilateral (N)
Didelphodon vorax† (NDGS 431)	6.54	5.29	2.15	1.76	30.00	196.29	158.70	4.96	5.46	6.45	283.03	257.12	217.65
Taxidea taxus	5.51	7.32	2.23	1.72	30.00	165.33	219.69	5.18	–	7.87	288.43	–	189.81
Lontra canadensis	6.26	7.05	1.94	1.45	30.00	187.80	211.41	3.62	–	5.77	370.03	–	232.55
Sarcophilus harrisii	13.20	14.99	2.19	2.18	30.00	395.85	449.76	7.07	–	9.25	523.35	–	400.24
Didelphis virginiana	5.12	5.90	1.85	1.84	30.00	153.48	177.09	4.30	–	6.60	283.54	–	184.69

Supplementary Table 7. Canine bite-force estimates adjusted for body-size allometery (canine bite force quotient, BFQ) for a sample of extant and extinct mammals. Body mass and canine bite-force estimates were taken from Wroe et al.[4] (table 1) except for *Lontra canadensis*, *Taxidea taxus*, one *Sarcophilus harrisii* specimen, *Didelphis virginiana*, and *Didelphodon vorax* (this study). BFQ was then calculated from a dataset-specific regression formula: log CBs = 1.79188 + 0.56414 * log body mass.

Species	Family	body mass (kg)	CBs (N)	BFQ
Alopex lagopus	Canidae	8.2	178	88
Canis alpinus	Canidae	16.5	314	104
Canis aureus	Canidae	7.7	165	84
Canis lupus dingo	Canidae	17.5	313	101
Canis lupus hallstromi	Canidae	12.3	235	92
Lycaon pictus	Canidae	18.9	428	132
Vulpes vulpes	Canidae	8.1	164	81
Urocyon cineroargentus	Canidae	5.3	114	72
Canis latrans	Canidae	19.8	275	82
Canis lupus lupus	Canidae	34.7	593	129
Canis dirus †	Canidae	50.8	893	157
Ursus americanus	Ursidae	105.2	541	63
Ursus arctos	Ursidae	128.8	751	78
Ursus thibetanus	Ursidae	77.2	312	43
Meles meles	Mustelidae	11.4	244	100
Taxidea taxus (this study)	Mustelidae	12.0	190	75
Lontra canadensis (this study)	Mustelidae	14.0	233	85
Gennetta tigrinus	Viverridae	6.2	73	42
Crocuta crocuta	Hyaenidae	69.1	773	114
Hyaena hyaena	Hyaenidae	40.8	545	109
Proteles cristatus	Hyaenidae	9.3	151	69
Panthera onca	Felidae	83.2	1014	135
Panthera tigris	Felidae	186.9	1525	129
Acinonyx jubatus	Felidae	29.5	472	113
Felis yagouaroundi	Felidae	7.1	127	68
Lynx rufus	Felidae	2.9	98	87
Felis concolor	Felidae	34.5	472	103
Felis sylvestris	Felidae	2.8	56	51

Neofelis nebulosa	Felidae	34.4	595	131
Panthera leo	Felidae	294.6	1768	116
Panthera pardus	Felidae	43.1	467	90
Smilodon fatalis †	Felidae	199.6	976	79
Nimbacinus dicksoni †	Thylacinidae	5.3	267	168
Thylacinus cynocephalus	Thylacinidae	41.7	808	159
Priscileo roskellyae †	Thylacoleonidae	2.7	184	170
Wakaleo vanderleurei †	Thylacoleonidae	41.4	673	133
Thylacoleo carnifex †	Thylacoleonidae	109.4	1692	193
Thylacosmilus atrox †	Thylacosmilidae	106.0	353	41
Dasyurus maculatus	Dasyuridae	3.0	153	133
Dasyurus viverrinus	Dasyuridae	0.9	65	114
Sarcophilus harrisii (Wroe et al. 2004)	Dasyuridae	12.0	418	166
Sarcophilus harrisii (this study)	Dasyuridae	12.0	400	159
Didelphis virginia (this study)	Didelphidae	6.0	185	109
Didelphodon vorax † (NDGS 431)	Stagodontidae	2.7	218	201

Supplementary Table 8. Relative premolar size (RPS) calculations for *Didelphodon vorax* using P3 measurements (Supplementary Table 3) and minimum and maximum body-mass estimates (Supplementary Table 4) for NDGS 431 and UWBM 94084.

Specimen	Body mass (kg)	Body mass cube root	Maximum P3 width (mm)	RPS
NDGS 431	Min = 2.1	1.28	6.39	4.99
NDGS 431	Max = 2.7	1.39	6.39	4.59
UWBM 94084	Min = 4.2	1.61	6.01	3.72
UWBM 94084	Max = 6.2	1.84	6.01	3.27

Supplementary Table 9. Measurements and calculations for canine bending strength of *Didelphodon vorax* and similar-sized extant mammals. TSL, total skull length; A-P radius, anteroposterior radius of the canine base; M-L radius, mediolateral radius of the canine base; height, height of the canine crown; Ix, second moment of area about the anteroposterior axis; Sx, bending strength about the anteroposterior axis; Iy, second moment of area about the mediolateral axis; Sy, bending strength about the mediolateral axis.

	log TSL	A-P radius	M-L radius	height	log height	Ix	Sx	log Sx	Iy	Sy	log Sy
Taxidea taxus	2.038	3.63	3.21	16.63	1.221	93.99	1.76	0.246	120.59	2.00	0.300
Didelphis virginiana (UWBM 12555)	2.086	3.59	2.47	16.34	1.213	42.43	1.05	0.022	89.38	1.53	0.184
Sarcophilus harrisii (UWBM 20671)	2.111	4.75	3.91	18.21	1.260	223.00	3.13	0.496	329.11	3.80	0.580
Didelphodon vorax† (NDGS 431)	1.976	3.00	2.10	10.48	1.020	21.82	0.99	-0.004	44.53	1.42	0.151
Didelphodon vorax† (UWBM 94084)	2.087	3.98	2.92	13.49	1.130	77.73	1.97	0.295	144.04	2.69	0.429

Supplementary Table 10. Canine bending strength of extant and extinct mammals. All data are from Van Valkenburgh and Ruff [5] except for *Taxidea taxus*, *Didelphis virginiana*, *Sarcophilus harrisii*, and *Didelphodon vorax* (this study). CL, canine length, and abbreviations from Supplementary Table 9.

Species	Family	log TSL	log CL	log AP	log ML	log Sx	log Sy
Vulpes velox	Canidae	1.996	1.083	0.638	0.498	-0.456	-0.315
Lycaon pictus	Canidae	2.268	1.318	1.037	0.845	0.402	0.594
Canis lupus	Canidae	2.337	1.426	1.151	0.881	0.479	0.749
Urocyon cinereoargenteus	Canidae	2.045	0.954	0.585	0.462	-0.452	-0.329
Vulpes vulpes	Canidae	2.106	1.154	0.756	0.618	-0.170	-0.032
Canis mesomelas	Canidae	2.149	1.197	0.803	0.585	-0.232	-0.014
Canis latrans	Canidae	2.214	1.297	0.908	0.703	0.010	0.216
Dusicyon culpaeus	Canidae	2.225	1.270	0.922	0.732	0.109	0.298
Alopex lagopus	Canidae	2.092	1.155	0.799	0.568	-0.228	0.004
Cerdocyon thous	Canidae	2.127	1.124	0.778	0.550	-0.253	-0.025
Nyctereutes procyonoides	Canidae	2.037	1.025	0.712	0.531	-0.259	-0.078
Otocyon megalotis	Canidae	2.083	1.017	0.618	0.512	-0.383	-0.277
Speothos venaticus	Canidae	2.124	1.130	0.869	0.728	0.188	0.328
Chrysocyon brachyurus	Canidae	2.336	1.403	1.053	0.857	0.357	0.552
Cuon alpinus	Canidae	2.234	1.255	1.004	0.763	0.268	0.509
Taxidea taxus	Mustelidae	2.038	1.221	0.861	0.807	0.246	0.300
Hyaena hyaena	Hyaenidae	2.311	1.404	1.125	0.975	0.664	0.814
Hyaena brunnea	Hyaenidae	2.356	1.418	1.215	1.066	0.921	1.070
Crocuta crocuta	Hyaenidae	2.362	1.407	1.167	1.019	0.791	0.939
Felis weidii	Felidae	1.989	1.037	0.724	0.607	-0.106	0.011
Felis yagourundi	Felidae	1.996	0.987	0.763	0.602	-0.027	0.134
Lynx rufus	Felidae	2.037	1.109	0.778	0.667	-0.004	0.107
Felis caracal	Felidae	2.045	1.170	0.881	0.720	0.143	0.304
Fleis aurata	Felidae	2.049	1.188	0.869	0.771	0.215	0.314
Felis temmicnckì	Felidae	2.097	1.107	0.878	0.763	0.290	0.404
Felis serval	Felidae	2.107	1.127	0.848	0.736	0.186	0.298
Velis viverrina	Felidae	2.112	1.246	0.949	0.816	0.328	0.462
Uncia uncia	Felidae	2.188	1.377	1.031	0.949	0.545	0.627
Puma concolor	Felidae	2.215	1.392	1.086	0.996	0.678	0.769
Neofelis nebulosa	Felidae	2.217	1.560	1.099	0.987	0.504	0.616
Panthera onca	Felidae	2.266	1.456	1.178	1.095	0.904	0.986

Acinonyx jubatus	Felidae	2.293	1.479	1.170	1.095	0.873	0.948
Panthera pardus	Felidae	2.297	1.504	1.157	1.017	0.679	0.819
Panthera leo	Felidae	2.410	1.640	1.296	1.188	1.023	1.131
Panthera tigris	Felidae	2.454	1.719	1.401	1.256	1.188	1.333
Didelphis virginiana (UWBM 12555)	Didelphidae	2.086	1.213	0.856	0.694	0.022	0.184
Sarcophilus harrisii (UWBM 20671)	Dasyuridae	2.111	1.260	0.978	0.893	0.496	0.580
Didelphodon vorax † (NDGS 431)	Stagodontidae	1.976	1.020	0.778	0.623	-0.004	0.151
Didelphodon vorax † (UWBM 94084)	Stagodontidae	2.087	1.130	0.900	0.766	0.295	0.429

Supplementary Table 11. Measurements and calculations of mandibular bending strength of *Didelphodon vorax*, *Sarcophilus harrisii*, and *Didelphis virginiana*, based on methods of Therrien[6]. Ix, second moment of area about the labiolingual axis; Zx, bending strength in the dorsoventral plane; Iy, second moment of area about the dorsoventral axis; Zy, bending strength in the labiolingual plane.

Species	Tooth position	Distance to condyle (L)	Dorsoventral radius (a)	Labiolingual radius (b)	Ix	Zx	Dorsoventral bending force (Zx/L)	Iy	Zy	Labiolingual bending force (Zy/L)	Relative force (Zx/Zy)
Didelphodon vorax †	canine	8.08	0.60	0.66	0.11	0.18	-1.64	0.13	0.20	-1.60	0.91
UWBM 102139	p2-p3	7.58	0.66	0.50	0.12	0.17	-1.64	0.07	0.13	-1.76	1.32
	p3-m1	7.00	0.73	0.48	0.15	0.20	-1.54	0.06	0.13	-1.72	1.52
	m1-m2	6.51	0.78	0.44	0.16	0.21	-1.50	0.05	0.12	-1.75	1.78
	m2-m3	6.00	0.84	0.44	0.21	0.25	-1.39	0.06	0.13	-1.67	1.93
	post m4	4.68	0.76	0.45	0.15	0.20	-1.36	0.06	0.12	-1.58	1.67
Didelphodon vorax †	canine	–	0.82	0.59	0.26	0.31	–	0.13	0.22	–	1.39
UCMP 159909	p2-p3	–	0.87	0.56	0.29	0.33	–	0.12	0.21	–	1.56
(m4 not preserved)	p3-m1	–	0.89	0.55	0.30	0.34	–	0.11	0.21	–	1.63
	m1-m2	–	0.88	0.47	0.25	0.28	–	0.07	0.15	–	1.87
	m2-m3	–	0.91	0.45	0.27	0.29	–	0.07	0.15	–	2.00
Sarcophilus harrisii	canine	9.51	0.96	0.97	0.67	0.70	-1.13	0.68	0.70	-1.13	0.99
UWBM 20671	p2-p3	8.42	1.02	0.47	0.40	0.39	-1.33	0.09	0.18	-1.67	2.16
	p3-m1	7.57	1.06	0.44	0.41	0.39	-1.29	0.07	0.16	-1.67	2.42
	m1-m2	6.57	1.03	0.48	0.41	0.40	-1.22	0.09	0.18	-1.56	2.17
	m2-m3	5.48	1.03	0.57	0.50	0.48	-1.06	0.15	0.27	-1.31	1.80
	post m4	4.13	1.20	0.53	0.72	0.60	-0.84	0.14	0.26	-1.19	2.28
Didelphis virginiana	canine	9.27	0.57	0.54	0.08	0.14	-1.82	0.07	0.13	-1.85	1.06
UWBM 12555	p2-p3	7.83	0.72	0.34	0.10	0.14	-1.75	0.02	0.07	-2.08	2.13
	p3-m1	7.04	0.73	0.33	0.10	0.14	-1.71	0.02	0.06	-2.05	2.19
	m1-m2	6.55	0.75	0.30	0.10	0.13	-1.71	0.02	0.05	-2.11	2.52
	m2-m3	5.95	0.74	0.30	0.09	0.13	-1.67	0.01	0.05	-2.07	2.50
	post m4	4.60	0.82	0.31	0.13	0.16	-1.45	0.02	0.06	-1.88	2.68

Supplementary Table 12. Microwear sampling of extant mammals. Abbreviations: N, number of specimens; ADO, animal-dominated omnivory; PDO, plant-dominated omnivory; Refs, references.

Family	Species	N	Dietary Category	Foods	Refs.
Didelphidae	*Philander opossum*	5	ADO	fruits, nectar, nuts, small mammals, birds, eggs, reptiles, amphibians, insects, crustaceans, snails, earthworms, and carrion	7,8
Procyonidae	*Bassariscus astutus*	4	ADO	Arthropods, mammals, birds, and fruits	7,9
Procyonidae	*Nasua narica*	5	ADO	Invertebrates, fruits, and vertebrates	10
Tupaiidae	*Tupaia glis*	6	ADO	Mainly insects, other animal foods, fruits, seeds, and leaves	7
Dasyuridae	*Antechinus stuartii*	5	Carnivore	Mainly invertebrates, especially arthropods, and small vertebrates	7
Macroscelididae	*Elephantulus rufescens*	4	Carnivore	Almost entirely insects and spiders	7,11
Muridae	*Hydromys chrysogaster*	6	Carnivore	Fish, insects, spiders, birds, crustaceans, mussels, frogs, turtles, and small mammals	12
Mustelidae	*Lontra candensis*	5	Carnivore	mostly fish, amphibians, and crustaceans	13
Viverridae	*Genetta tigrina*	2	Carnivore	Rodents, birds, reptiles, and insects	7
Potoroidae	*Aepyprymnus rufescens*	2	Herbivore	Herbs and grasses, roots and tubers, fungi, invertebrates, gum, flower, fruit, seed, leaf, and stem	7,14
Procyonidae	*Procyon lotor*	5	Omnivore	Crayfish, crabs, other arthropods, frogs, fish, nuts, seeds, acorns, and berries.	7,15
Cricetidae	*Ondatra zibethicus*	6	PDO	Mostly hydrophytes; crayfish, fish, and mollusc if abundant or during food shortages	16
Potoroidae	*Potorous trydactylus*	5	PDO	Roots, tubers, fungi, arthropods, insects, fruits, seeds, grass, and other plant parts	7,14,17,18
Procyonidae	*Potos flavus*	8	PDO	Fruits, seeds, flowers, honey, insects, and small mammals	7,19
Procyonidae	*Procyon cancrivorus*	5	PDO	Fruit, pulp, crustaceans, other arthropods, and small vertebrates	20,21
Sciuridae	*Spermophilus lateralis*	4	PDO	Fungi, seeds, acorn, nuts, shrubs, herbs, insects, eggs, birds, small mammals, lizards, and carrion	22

Supplementary Table 13. Results of intra-observer microwear error test. All analyses: 18 degrees of freedom.

Feature	Spearman's rho	p
All pits	0.958	$3.59.10^{-11}$
All scratches	0.936	$1.27.10^{-9}$
Small pits	0.923	$6.85.10^{-9}$
Cross scratches	0.839	$3.73.10^{-6}$

Supplementary Table 14. Mean microwear signatures of extant taxa sampled. Abbreviations: Abb, abbreviation; N, sample size; SP, small pits; LP, large pits, SPP, small puncture pits; LPP, large puncture pits; CRS, cross scratches; G, gouges; FS, fine scratches; COS, coarse scratches; HS, hypercoarse scratches; StdDev, standard deviation.

Taxon	Abb	Statistic	N	SP	LP	LPP	SPP	CRS	G	FS	COS	HS
Bassariscus astutus	Ba	Mean	4	37	1	1	2	42	0	9	6	2
		StdDev		5	1	2	4	13	0	6	7	1
Nasua narica	Nn	Mean	5	33	3	2	2	47	0	7	4	2
		StdDev		10	3	2	3	11	1	4	3	1
Philander opossum	Po	Mean	5	43	4	2	1	46	0	4	0	0
		StdDev		3	1	2	1	9	0	8	1	0
Tupaia glis	Tg	Mean	6	45	4	2	1	38	0	7	3	1
		StdDev		5	4	2	1	7	0	5	1	2
Antechinus stuartii	As	Mean	5	50	4	1	0	33	0	10	2	0
		StdDev		3	3	1	0	10	0	11	1	0
Elephantulus rufescens	Er	Mean	4	47	7	1	1	38	0	5	2	0
		StdDev		11	5	2	2	14	0	5	3	1
Genetta tigrina	Gt	Mean	2	38	2	1	5	36	2	11	5	2
		StdDev		5	2	1	7	7	2	0	2	1
Hydromys chrysogaster	Hc	Mean	6	44	3	2	2	46	0	1	1	1
		StdDev		4	2	2	3	2	0	3	1	1
Aepyprymnus rufescens	Ar	Mean	2	25	2	0	2	55	0	3	6	7
		StdDev		8	3	0	3	4	0	4	1	6
Lontra canadensis	Lc	Mean	5	21	3	2	1	52	0	4	4	2

		StdDev		3	1	2	1	4	0	7	3	1
Ondatra zibethicus	Oz	Mean	6	24	1	3	1	65	0	2	2	1
		StdDev		3	1	4	2	9	0	2	1	1
Potorous trydactylus	Pt	Mean	5	25	7	2	1	42	0	16	5	2
		StdDev		4	5	3	3	11	0	10	3	1
Potos flavus	Pf	Mean	8	25	3	1	2	62	0	5	2	1
		StdDev		4	3	1	3	8	0	9	2	1
Procyon cancrivorus	Pc	Mean	5	41	2	4	1	50	0	0	1	0
		StdDev		5	2	1	1	3	0	1	2	1
Procyon lotor	Pl	Mean	5	40	3	3	1	42	0	5	3	2
		StdDev		5	4	3	2	7	0	4	2	2
Spermophilus lateralis	Sl	Mean	4	39	0	4	8	47	0	0	1	1
		StdDev		3	0	1	4	4	0	0	1	1

Supplementary Table 15. Microwear of *Didelphodon vorax* specimens included in our analysis. Values reported are percentages of microwear features. Abbreviations: SP, small pits; LP, large pits, SPP, small puncture pits; LPP, large puncture pits; CRS, cross scratches; G, gouges; FS, fine scratches; COS, coarse scratches; HS, hypercoarse scratches; StdDev, standard deviation. Museum abbreviations: NDGS, North Dakota Geological Survey; UCMP, University of California Museum of Paleontology; UWBM, University of Washington Burke Museum.

Specimen	SP	LP	LPP	SPP	CRS	G	FS	COS	HS
UWBM 92635	26	4	1	3	53	0	9	4	0
UCMP 187702	16	3	7	3	57	0	9	5	1
UCMP 187863	25	1	1	4	52	0	13	2	1
UCMP 187883	24	3	3	3	50	0	14	5	0
UCMP 187607	29	4	2	7	55	0	2	2	0
UCMP 174298	32	0	3	3	57	0	3	1	0
NDGS 431	28	0	3	4	58	0	3	3	1
Mean	26	2	3	4	55	0	8	3	0
StdDev	5	2	2	2	3	0	5	2	1

Supplementary Notes

Supplementary Note 1: Description and comparison of new specimens of *Didelphodon vorax*

Terminology and measurements—Terminology for cranial osteology follows Wible[23]. Dental terminology follows Kielan-Jaworowska et al.[24]. The postcanine dental formula for Metatheria was recently re-interpreted as P1/p1, P2/p2, P4/p4, DP5/dp5, M1/m1, M2/m2, and M3/m3[25,26], but we use the traditional terminology of three premolars and four molars for practical purposes. Dental measurements (Supplementary Table 3) follow Lillegraven and Bieber[27] for molars. Dental measurements were taken using a Leica MZ9.5 binocular dissecting microscope and custom measuring stage capable of reading to 0.001 mm.

Nasal—In UWBM 94084, the nasals are broad medial to the premaxillae, and then narrow posteriorly before expanding again (Supplementary Figs. 3a,c and 4a,c). They are broken anteriorly. After the CT scanning had been completed, a bone fragment that was encased in sediment in the nares was mechanically prepared out and was affixed to the anterior end of the right nasal. It shows that the nasals extended anteriorly along the midline at least another 4 mm (Supplementary Fig. 4c); whereas the exposed medial edges of the posterodorsal projections of the premaxillae show no indications that the nasals extended along the premaxillo-nasal suture beyond what is preserved. That is, the nasals are retracted relative to most other metatherians examined except the undescribed marsupialiform 'Gurlin Tsav skull,' in which this is also the condition (Szalay and Trofimov[28]:fig. 22). Posterior to the infraorbital foramen, the rostrum is not preserved in UWBM 94084. In NDGS 431, the posterior rostrum is crushed, dislodged, and rotated from its natural position, but the margins of the nasals and their sutures with adjacent

bones are approximated by differences in bone texture (Supplementary Figs. 1d and 2f). The lateral expansion of the nasals begins dorsal to the P3–M1 embrasure, and then posteriorly (dorsal to M3) the nasals taper toward the midline, terminating dorsal to M4. The degree of expansion in *Didelphodon vorax* is similar to that in *Didelphis virginiana*, *Monodelphis brevicaudata*, *Pucadelphys andinus*, *Andinodelphys cochabambensis*, *Mayulestes ferox*, *Herpetotherium fugax*, and the 'Gurlin Tsav skull,' although the point of greatest expansion occurs more posteriorly in these other taxa (dorsal to M1, M2, or M3). The naso-frontal suture appears to have the broad V-shape form of most living didelphids[29] and fossil metatherians[28,30-33].

Premaxilla—The isolated right and left premaxillae of NDGS 431 are fragmentary (Supplementary Figs. 1 and 2), whereas in UWBM 94084 (Supplementary Fig. 3 and 4), the premaxillae are more completely preserved. The overlapping elements in NDGS 431 and UWBM 94084 are morphologically consistent, so we base our description of the premaxilla and the rest of the anterior rostrum on a composite of the two specimens, noting differences between the specimens.

The paired premaxillae and maxillae form a broad, blunt snout. Each premaxilla consists of an alveolar process, a facial process, an internarial process, and a palatal process. The alveolar process contains four alveoli for single-rooted incisors (Supplementary Figs. 1e, 2g, 3d–e, and 4d); I1 is preserved in UWBM 94084. The alveolar row is relatively transverse as in *Sarcophilus harrisii* and in contrast to the arched alveolar row of *Didelphis virginiana*, *Monodelphis brevicaudata*, *Pucadelphys andinus*, *Andinodelphys cochabambensis*, *Mayulestes ferox*, *Herpetotherium fugax*, and the 'Gurlin Tsav skull.' Above the alveoli, the narial platform of the

premaxilla is dorsoventrally tall. At the midline, the paired premaxillae meet to form a robust dorsally projecting internarial process that partially bisects the external nasal aperture (Supplementary Figs. 1a,c,d, 2a,b,e, 3a–c, and 4a–c). The posterodorsal tip of this process is slightly broken in UWBM 94084, but in NDGS 431, where this process is complete, it does not extend much beyond what is preserved in UWBM 94084. Among fossil therians, this internarial process is otherwise only known in the 'Gurlin Tsav skull' (Szalay and Trofimov[28]:fig. 22), although few early metatherians are known by skulls that preserve the anteriormost snout; a smaller and more pointed process occurs in the water opossum *Chironectes minimus* (http://www.digimorph.org/specimens/Chironectes_minimus/) and the Late Cretaceous gondwanatherian *Vintana sertichi*[34]. An internarial process that fully bisects the external nasal aperture is plesiomorphic among basal mammaliaforms (e.g., *Sinoconodon*, *Haldanodon*)[24] and therapsids (e.g., *Procynosuchus*)[35]. Extrinsic snout musculature (e.g., *M. maxillonasolabialis*) likely attached to the roughened anterior surface of this process to form a highly mobile snout in *D. vorax*[36].

In UWBM 94084, the facial process of the premaxilla ascends between the maxilla and nasal as a thin posterodorsal projection, tapering gradually to its termination dorsal to the P2–P3 embrasure (Supplementary Figs. 3a–c and 4a–c), as in the 'Gurlin Tsav skull' (Szalay and Trofimov[28]:fig. 22). In *Didelphis virginiana*, *Monodelphis brevicaudata*, *Andinodelphys cochabambensis*, *Pucadelphys andinus*, and *Mayulestes ferox*, the posterodorsal projection of the facial process is much broader, more laterally exposed, and terminates more abruptly, dorsal to the canine or P1. The lateral walls and the ventral floor of the nasal orifice are visible, in ventral view.

In UWBM 94084, the palatal processes of the premaxillae bound the anterior, medial, and lateral margins of the large, paired incisive foramina and posteriorly contact the maxillae (Supplementary Figs. 3d and 4d). On each side, the medial palatal process of the premaxilla, which forms the medial border of the incisive foramen, broadens posteriorly to more than three times its anterior width; this broadening is more pronounced than in *Didelphis virginiana, Monodelphis domestica*[23], and the 'Gurlin Tsav skull' (Szalay and Trofimov[28]:fig. 22). The posterior margin of the medial palatal process of the premaxilla is inclined dorsally relative to the anterior margin of the palatal process of the maxilla; this appears to be the natural position but we cannot rule out whether the premaxillae have been dislodged dorsally. The shape of each incisive foramen is broader and more posterolaterally directed than in *Didelphis virginiana* and the 'Gurlin Tsav skull' and more similar to that in *Monodelphis domestica*, wherein the posterior margin of the foramen has a distinctive undulation lateral to the midline. The incisive foramen does not extend posteriorly beyond the midpoint of the upper canine, which is similar to the condition in *Didelphis virginiana*, *Monodelphis domestica*, and the 'Gurlin Tsav skull,' but because of the blunter snout in *Didelphodon vorax*, the length of the foramen is shorter relative to the length in those taxa. The incisive foramina are also visible in dorsal view through the nasal orifice, which is the case for the 'Gurlin Tsav skull' as well.

In UWBM 94084, the premaxillo-maxillary suture extends from the midline of the paracanine fossa, which receives the lower canine, posterodorsally to the termination of the facial process of the premaxilla, whereupon the maxilla contacts the nasal (Supplementary Figs. 3a–c and 4a–c). The anterior surface of the maxilla and premaxilla is slightly concave medial to the upper canine

root. This is commonly found in taxa with well-developed canines, but because of the bluntness of the snout in *Didelphodon vorax*, the concavity is on the anterior rostrum, rather than on the lateral rostrum as in *Didelphis virginiana*. Many nutrient foramina on the anterolateral surface of the maxilla suggest extensive mucoperiosteum and enlarged gingivae in this area[37].

Maxilla—The lateral surface of the maxilla is reconstructed from overlapping parts of UWBM 94084, NDGS 431, and UWBM 94500. In UWBM 94084, swelling occurs on the lateral maxilla just above the canine and extends posteriorly at this level to form an overhang dorsal to the tooth row. It is most pronounced dorsal to P3 and ventral to the infraorbital foramen (Supplementary Figs. 3b and 4b). A shallow depression anterior to the infraorbital foramen possibly represents an attachment area for extrinsic snout musculature[36]. The ventral, medial, and dorsal margins of the infraorbital foramen (not the lateral margin) are preserved in UWBM 94084. The main part of NDGS 431 preserves the ventrolateral margin of the infraorbital foramen on the left maxilla, and an isolated fragment of NDGS 431 preserves a part of the right maxilla just anterior to the infraorbital foramen (Supplementary Figs. 1c and 2c). UWBM 94500 preserves the ventral and posterior margins of the infraorbital foramen (Supplementary Figs. 5e and 6e). In these specimens, the infraorbital foramen is dorsal to the P3–M1 embrasure and just anterior to the anterior margin of the jugal. The long axis of the foramen outline is dorsoventral to dorsomedial-ventrolateral. It is reconstructed as larger and more oval-shaped than in *Didelphis virginiana*, *Monodelphis brevicaudata*, and *Sarcophilus harrisii* and similar to the relative size in *Chironectes minimus*. Small extant semiaquatic mammals possess larger infraorbital foramina than their terrestrial relatives[38,39], reflecting passage of a relatively larger infraorbital nerve for

increased sensory adaptation of vibrissae in an aquatic habitat. Posteriorly, the maxilla forms the ventral aspect of the anterior zygomatic root dorsal to M3 and M4.

In UWBM 94500 and SCNHM VMMa 20 (Supplementary Figs. 5c,g and 6c,g), the orbital floor formed by the maxilla is very broad relative to that in similarly sized extant didelphids (e.g., *Didelphis virginiana*). The plate is triangular-shaped with a roughened dorsal surface that extends from the level of M1 to M4. The posterolateral margin of the floor forms a smooth, rounded slope ventral to the alveolar margin.

UWBM 94084 (Supplementary Figs. 3d and 4d) preserves the anterior palate including the premaxillae and maxillae from the incisors to the P3. UWBM 94500 (Supplementary Figs. 5h and 6h) includes parts of the palatal process of the maxilla from the P3 to M4. NDGS 431 (Supplementary Figs. 1e and 2e) preserves the posterior palate including the parts of the maxillae and palatines from the P3 to the M4. However, portions of NDGS 431 have been crushed, dislodged, and rotated, so the natural position and morphology of these bones is cautiously interpreted.

The palatal processes of the paired maxilla form a relatively flat ventral surface with only slight dorsal convexity along the anteroposterior axis. The breadth of the palate is consistent along its preserved length in UWBM 94084, from the canine to P3 (Supplementary Figs. 3d and 4d). From the lateral margin of the incisive foramen, a low, laterally convex ridge extends posteromedially, separating a broad groove laterally from a shallow depression that is immediately posterior to the incisive foramen. The ventral surface of the palatal process of the

maxilla is perforated by numerous small nutrient foramina in addition to a few large foramina along the lateral margin (one near the canine, one near the P1, and two near the P3) and the largest foramen near the midline suture lateral to the P2. The latter foramen is not found in any of the extant specimens examined. The paired maxillae come together at the midline to form a low, roughened suture ridge that increases in width posteriorly and is intermittently perforated, similar to what is observed in the river otter *Lontra canadensis*.

The palatal process of the maxilla of NDGS 431 is damaged from the M1 to M3 (Supplementary Figs. 1e and 2e). Some of this missing morphology is preserved in UWBM 94500 (Supplementary Figs. 5h and 6h). Medial to the M1–M2 embrasure is a gently curved natural edge that is interpreted as the anterior and anterolateral margins of a maxillopalatine fenestra. Palatal fenestration is absent in deltatheroidan metatherians, *Pucadelphys andinus*, *Mayulestes ferox*, and borhyaenoid marsupialiforms[40]. Based on the degree of curvature, the fenestra is broad relative to those in *Didelphis virginiana* and *Monodelphis domestica*. The distance between the lateral margin and the maxillary suture also implies that a broad median septum separated the paired maxillopalatine fenestrae. In medial view, the smooth edge of the fenestra margin gives way to an irregular edge posteriorly. Although this edge may simply represent a broken margin, sutural morphology in *Didelphis virginiana* suggests that it represents the contact with the palatine; ventral view of the specimen corroborates this interpretation.

Palatine—The posterior-most portion of the palate is preserved in NDGS 431 (Supplementary Figs. 1e and 2e). Each palatine preserves the posterior and medial margins of a broad fenestra that extends from the M3 to M4. It is uncertain whether this fenestra represents the posterior end

of the maxillopalatine fenestra that is represented in UWBM 94500 or whether it represents a separate palatine fenestra. The very narrow median septum differs from the inferred breadth of the median septum at the anterior margin of the maxillopalatine fenestra, but the limited space available to accommodate a second fenestra suggests that this posterior margin is likely confluent with the maxillopalatine fenestra. There do not appear to be fenestrae that are restricted to the palatine. Thus, we reconstruct the palatal fenestration to be very similar to the condition in the 'Gurlin Tsav skull' (Szalay and Trofimov[28]:fig. 22). However, palatal fenestration in extant marsupials is highly variable in terms of occurrence, size, and position[29]. For example, *Didelphis virginiana* exhibits maxillopalatine and palatine fenestrae, whereas *Monodelphis domestica* exhibits only maxillopalatine fenestrae.

The posterior border of the palate of NDGS 431 (Supplementary Figs. 1e and 2e) is low and broadly arched and gently inflected ventrally without prominent lateral corners (lateral postpalatine spines of Marshall et al.[31]), similar to the condition in *Caluromys*, *Caluromysiops*, *Glironia*, *Mayulestes ferox*[41], and the 'Gurlin Tsav skull' and in contrast to that in *Marmosa*, *Didelphis*, most other didelphids[29], and *Pucadelphys andinus*[31]. It also shares with the former taxa weakly constricted internal choanae. The minor palatine foramen forms a deep depression and small perforation through the maxillo-palatine suture posterolingual to the M4; this is in contrast to the condition in most didelphids (ref. 29:fig. 15). On the right side, there is a ventrally open channel that traverses the posterior margin of the palate just posterior to the minor palatine foramen. On the left side, this channel is a small incomplete perforation of the posterior margin of the palate, a feature not observed among any of the comparative extant didelphids.

Jugal—The dorsal component of the anterior zygomatic root is formed by the jugal, which extends onto the lateral rostrum dorsal to M1 and just posterior to the infraorbital foramen in UWBM 94500 and SCNHM VMMa 20 to form an anterodorsally oblique suture with the maxilla (Supplementary Figs. 5e and 6e). In *Didelphis virginiana* and *Metachirus nudicaudatus*, the anterior edge of the jugal is also dorsal to M1, but the infraorbital foramen tends to be more anterior. A roughened maxillary boss for the origin of the superficial masseter muscle or extrinsic snout musculature (*M. maxillonasolabialis*) extends across the maxillo-jugal suture dorsal to M3–M4. This boss is more pronounced than it is in *Didelphis virginiana*, particularly in UWBM 94500 and SCNHM VMMa 20, both of which are larger individuals that perhaps represent later ontogenetic stages[42] or sexual dimorphs[43] relative to NDGS 431 (Supplementary Figs. 1d and 2d). In UWBM 94500, the infraorbital canal is very short, perhaps because of the relatively blunt snout of *Didelphodon vorax*. As a result, the anterior margin of the jugal nearly contacts the infraorbital foramen. The dorsal contacts of the anterior jugal with the maxilla and lacrimal cannot be precisely determined. Posteriorly, the zygomatic arch, formed by the jugal and squamosal, is robust, lacks a prominent frontal process, and has a modest suborbital deflection (Supplementary Figs. 1c and 2c). The jugal forms the lateral margin of the orbit dorsal to the M4. Dorsoventral distortion of NDGS 431 probably had little effect on suborbital deflection. By comparison with large didelphids (e.g., *Lutreolina*), this combination of features suggests that *Didelphodon vorax* might have had relatively small eyes and massive masticatory muscles[29].

Frontal—The interorbital region exhibits considerable crushing in NDGS 431 (Supplementary Figs. 1c,d and 2c,d). The interorbital breadth gently narrows posteriorly without an abrupt

constriction. There is no postorbital process or supraorbital margin on the frontals; however, their absence may be due to poor preservation or to the young ontogenetic stage of NDGS 431. For example, frontal outgrowths develop late in the postweaning stage of extant didelphids (e.g., *Didelphis albiventris*)[42]. A low, distinct sagittal crest extends from the frontals posteriorly to the parietals and to a robust lambdoidal crest on the interparietal.

Parietal—In NDGS 431, the cranial roof is low, broadly convex, and twice as wide as the interorbital region (Supplementary Figs. 1d and 2d). Bone texture suggests that the fronto-parietal suture occured at paired breaks on the left and right sides of the cranial roof, posterior to the narrowest interorbital breadth and to the anterior extension of the squamosal on the zygomata. The parietal appears to contact the alisphenoid anterolaterally, as is the case in most didelphids[29], the squamosal laterally, and the interparietal posteriorly.

Interparietal—In dorsal view, the lambdoidal crest is posteriorly convex with an anterior emargination at the midline. Subtle differences in bone texture and a suture with the supraoccipitals ventral to the lambdoidal crest implies the presence of an interparietal bone, as in all didelphids and some non-didelphid marsupials[29]. The interparietal appears to contact the squamosal laterally, as is the case in some didelphids (e.g., *Didelphis*) but not in most non-didelphid marsupials[29].

Pterygoid—In ventral view of NDGS 431 (Supplementary Figs. 1e and 2e), the nasopharygneal region is broad and U-shaped anteriorly, rather than posteriorly tapering as in *Didelphis*

virginiana. The pterygoids appear to be partially preserved, but the contact with the medial surface of the perpendicular process of the palatine is unclear.

Presphenoid—A splint-like pre-sphenoid is preserved.

Basisphenoid—The basisphenoid is fragmentary, particularly the anterior half, but the posterior contact with the basioccipital and the lateral contact with the alisphenoid is preserved on the left side. A distinct midline ridge extends across the basisphenoid-basioccipital suture.

Orbitotemporal Region—In NDGS 431, there is considerable damage to the orbitotemporal region. The anterior and dorsal margins of the orbit that would be formed by the lacrimal and frontal are mostly crushed in NDGS 431; likewise, the identity of the individual bones on the medial wall of the orbit (e.g., alisphenoid, palatine, frontal), the contacts among them, and the relevant foramina cannot be determined among the crushed fragments.

Squamosal—The squamosal overlaps the jugal from about the midpoint of the zygomatic arch to the glenoid fossa. The glenoid fossa is broad and hemi-cylindrical with a well-developed postglenoid process and a lesser-developed preglenoid process. The glenoid fossa is mainly formed by the squamosal. The jugal contributes anterolaterally to the preglenoid process; the alisphenoid contacts the squamosal laterally, but does not contribute to the glenoid fossa (Supplementary Figs. 1e and 2e). The broad, hemi-cylindrical shape of the glenoid fossa and the well-developed postglenoid process and less-developed preglenoid process imply a robust jaw articulation for *D. vorax*.

Tympanic Bulla— A ventrally projecting tympanic process of the alisphenoid is expanded on the left side of NDGS 431 to floor a hypotympanic sinus and contribute the anterolateral half of a tympanic bulla surrounding the middle ear cavity (Supplementary Fig. 7a). The posterior and medial constituent of the bulla contacts the rostral epitympanic wing and medial border of the petrosal (Supplementary Fig. 7b), as well as the caudal tympanic process of the petrosal to enclose the tympanic cavity in bone. Thus, the tympanic cavity is not open ventrally as observed in many extant marsupials[44]. The right tympanic bulla was not preserved *in situ*, but rather as a partial isolated element with a portion of the tympanic process of the alisphenoid attached. The morphology of the right bulla agrees with that described for the left, and it confirms that the tympanic process of the alisphenoid forms a hypotympanic sinus.

The bone contributing to the posterior (non-alisphenoid) portion of the bulla is unclear. A complete tympanic bulla has not been formally identified and described for *Didelphodon vorax*, although previous authors suggested that the bulla was composed of both alisphenoid and petrosal in *Didelphodon* and other stagodontid marsupialiforms[45,46]. In general, Gabbert[47] described three conditions of bulla composition observed in metatherians: a minimal condition in which the bulla is open ventrally and comprises uninflated tympanic process of the alisphenoid and rostral tympanic process of the petrosal (e.g., *Metachirus*), a "tripartite" condition in which the bulla is open ventromedially and comprises uninflated tympanic process of the alisphenoid and rostral tympanic process of the petrosal with an inflated posterior crus of the ectotympanic (e.g., *Monodelphis*[23]), and a maximal condition in which the tympanic process of the alisphenoid contributes over half of the bullar floor and contacts the rostral tympanic process of the petrosal,

thereby closing the bulla ventrally (e.g., *Caluromys*[44]). In addition, the entotympanic contributes a large portion of the bulla in *Dromiciops*[44].

Images produced during CT scanning reveal that the medial edge of the bulla contacts the medial border of the petrosal (Supplementary Fig. 7b), which suggests that the petrosal is the second major contributor to the bulla as it is in *Caluromys*. A true rostral tympanic process of the petrosal (*sensu* Horovitz et al.[30]) is absent in *Didelphodon*, but a low crest is present along the posteromedial edge of the promontorium that contacts the bulla. The posterior portion of the bulla appears to be separated from the rostral tympanic process of the petrosal, although the division likely is a result of damage to the basicranium as numerous cracks occur throughout the petrosal on the left side of the skull (white arrows in Supplementary Fig. 7b), but there are no fractures in the same region on the right side. Thus, we tentatively identify the posterior portion of the bulla as arising from the petrosal, as hypothesized by Clemens[45,46]. The tympanic process of the alisphenoid contributes over half of the bullar floor in the Late Cretaceous *Asiatherium* and the 'Gurlin Tsav skull'[28]. However, the bulla is more extensive in *Asiatherium* than the 'Gurlin Tsav skull' in that a structure identified as the rostral tympanic process of the petrosal contributes to the bulla in *Asiatherium*[28]. In this regard, the tympanic process of the alisphenoid in *Didelphodon* appears more similar to that of *Asiatherium*. Neither the bulla of *Asiatherium* or the 'Gurlin Tsav skull' completely encapsulates the middle ear cavity[28], although the elements may be incompletely preserved in both Asian specimens.

Although it appears that the posterior portion of the bulla of *Didelphodon* is contributed by the petrosal, an anteromedial expansion of the ectotympanic cannot be ruled out as a contributor at

this time, either. The ectotympanic, if present, could not be distinguished from the surrounding bones. It may be that the ecotympanic is intrabullar (“aphaneric”) as is the case in extant *Caluromys*[47], although the bone is not observed in CT images. Alternatively, the ectotympanic could be expanded to contribute to the posterior portion of the bulla as observed in *Monodelphis*[23] but to a greater degree than in the extant marsupial. A final possibility is that the ectotympanic was present in life but not preserved with the rest of the skull.

The anteromedial border of the petrosal contribution to the tympanic bulla is notched by a sulcus that extends along the medial border of the tympanic process of the alisphenoid before opening into the anterior carotid foramen and associated canal posteromedial to the foramen ovale (Supplementary Fig. 7a). Both the foramen ovale and anterior carotid foramen take a posterior position to what is observed in extant marsupials. The transverse suture separating the basisphenoid from the basioccipital medially and petrosal laterally is partially obscured by fractures, but it appears that the petrosal contributes at least the posterior border of the anterior carotid foramen thereby indicating that the foramen is not fully within the basisphenoid as is the case with extant marsupials. The carotid canal extends anterodorsally and ultimately opens into the cranial cavity posterolateral to the sella turcica. In life, the internal carotid artery would take an extrabullary course as it does in extant marsupials[48,49], but in contrast to some Paleocene marsupialiforms from South America[41,50,51].

The tympanic process of the alisphenoid contributes to the bulla in several extinct and extant metatherians[23,44,47,52-54], suggesting that the structure is plesiomorphic for Marsupialia. However, the bulla is absent in some early Paleocene marsupialiforms, including *Pucadelphys* and

Mayulestes[41,50,51], leading to the supposition that the alisphenoid contribution to the bulla evolved more than once within Metatheria. However, that hypothesis was based in part on the hypothesized absence of a bulla in stagodontids such as *Didelphodon*. Now that a complete bulla is known for *Didelphodon*, it is more parsimonious that the alisphenoid bulla is ancestral for metatherians and secondarily lost in *Pucadelphys* and *Mayulestes*, as suggested by Kielan-Jaworowska and Nessov[52].

Petrosal—The generalized petrosal bone of therian mammals consists of the anterior pars cochlearis, which houses the cochlear canal containing the spiral organ of hearing, and the pars canalicularis, which houses the organs of balance and equilibrium within the bony vestibule, semicircular canals, and associated ampullae. Much of the anatomy of the petrosal confirms the morphology described by Clemens[45]. In ventral aspect, the left petrosal is shielded from view by the tympanic bulla (Supplementary Fig. 7). The left bulla was digitally removed from 3D reconstructions skull rendered from CT data to reveal that the ventral surface of the pars cochlearis is damaged, but the pars canalicularis is more-or-less complete. The right petrosal is exposed and easily visible without the use of CT imaging. Although the pars cochlearis is complete on the right side of the skull, the pars canalicularis is damaged and the ventrally projecting mastoid process is missing.

The most distinctive feature of the pars cochlearis in ventral view is the promontorium containing the cochlea itself (Supplementary Fig. 8). The promontorium is rounded posteroventrally, and the bone is fairly thin ventral to the internal cochlear cavity, but thick medial, lateral, anterior, and dorsal to the bony labyrinth, as revealed by the CT data

(Supplementary Fig. 7b). A flattened epitympanic wing is present and extends anteromedial to the portion of the bone containing the cochlear cavity. The surface of the promontorium is undulatory, and there are no transpromontorial sulci for vascular or nervous structures. This contrasts the condition of borhyaenids and allied taxa, in which the anterior border of the petrosal is notched by a sulcus for the inferior carotid artery[40].

The caudal face of the promontorium is partially obstructed from ventral view by a posteriorly projecting shelf of bone. Although the shelf arises from a similar position to the rostral tympanic process of many metatherians (e.g., *Monodelphis*[23]), the structure is not developed as a process, but rather a bony ridge (see discussion of the rostral tympanic process in supporting information of Horovitz et al.[30]). The shelf is extended anteroventrally, and it contacts and may be continuous with the posterior portion of the tympanic bulla (Supplementary Fig. 7b), which ultimately articulates with the tympanic process of the alisphenoid anteriorly. The suture between the tympanic process of the alisphenoid and the tympanic bulla of the petrosal is dorsal to the extrabullar sulcus for the internal carotid artery (see discussion of tympanic bulla above).

The fenestra cochleae along the posteromedial region of the promontorium is small with thick walls compared to other regions of the promontorium, but neither the right or left fenestrae vestibuli are preserved completely, so the shape and size of the fenestra vestibuli could not be evaluated. The crista interfenestralis (bridge of bone separating the fenestrae cochleae and vestibuli) was damaged on the right side during preparation, and the lateral border of the fenestra vestibuli that separates the fenestra from the tympanic aperture of the facial canal (secondary facial foramen) is broken on the left side. The ventral floor of the facial canal is damaged,

exposing the course of the facial nerve on both sides of the skull. The bony channel for the greater petrosal branch of the facial nerve (aqueductus Fallopii) extends anteriorly from the tympanic aperture of the facial canal to the hiatus Fallopii along the anterior edge of the petrosal. The cavum supracochleare is completely enclosed and separated from the cavum epiptericum by a complete wall of bone, and the sulcus for the hyomandibular branch of the facial nerve curves ventrally posterior to the promontorium and across the anteromedial face of the robust mastoid process. A stylomastoid foramen is completed by the tympanohyal posteromedailly, the mastoid process posterolaterally, and the petrosal contribution of the bulla anteriorly.

The caudal tympanic process forms the posterior wall of the fossa for the stapedius muscle immediately posterior to the promontorium, and the process defines the medial edge of the facial nerve sulcus on the proximal end of the large mastoid process. The CT images reveal that a ridge of bone forming the inner curve of the semicircular canal defines the medial border of the stapedius fossa. Most of the anterior face of the mastoid process contributes to the posterodorsal wall of the external acoustic meatus, which is completed by the squamosal. Anteromedial to the external acoustic meatus is the epitympanic recess that is not uniformly concave, although much detailed morphology is difficult to determine on account of preservation. The posterior exposure of the mastoid process (pars mastoideus of the petrosal) is flattened, relatively large, and roughly triangular in shape. It contacts the squamosal, interparietal, and supraoccipital, and on the right side, the mastoid exhibits a robust but broken ventral process. This process would have provided a robust attachment point for the digastric muscle and several neck muscles (e.g., *M. sternocleidomastoideus*), which would have acted in jaw mechanics and prey capture, respectively.

The endocranial surface of the petrosal is distinguished by two large openings (Supplementary Fig. 8), the anteromedial of which is the internal acoustic meatus for passage of cranial nerves VII (facial) and VIII (vestibulocochlear). The crista transversa separating the foramina for the facial and vestibulocochlear nerves (*sensu* MacIntyre[55]) is positioned deep within the tubular meatus. A broad fossa contributing the medial wall of the cavum epiptericum for the trigeminal ganglion is developed on the anterolateral surface of the petrosal lateral to the prefacial commissure (Supplementary Fig. 8a–b). The subarcuate fossa (fossa subarcuata) is a deep excavation posterolateral to the internal acoustic meatus although the floors of the left and right fossae are incomplete and a portion the rim containing a segment of the anterior semicircular canal has been crushed on the left side of the skull. The circuit of the anterior semicircular canal bounds the osculum (aperture) of the subarcuate fossa, and the fossa is mushroom-shaped with an expansion beyond the apices of the semicircular canal arcs. Thus, the osculum of the fossa is narrower than the internal cavity.

The small aperture for the bony channel of the vestibular aqueduct (membranous endolymphatic duct) opens along the posteromedial rim of the subarcuate fossa (Supplementary Fig. 8a–b). The sulcus for the sigmoid sinus extends anteriorly from the posteromedial corner of the petrosal to an oval depression posterior to the small aperture for the vestibular aqueduct. Within the oval depression is the opening to a short canal that ultimately opens into the subarcuate fossa, perhaps to transmit a vein from the subdural space around the paraflocculus of the cerebellum to the sigmoid sinus. Anteromedial to the oval depression is the external aperture of the canaliculus cochleae (for the membranous perilymphatic duct) along the dorsal edge of the medial surface of

the petrosal. There is no indication of sulci for the superior petrosal or prootic sinuses on the lateral side of the endocranial surface of either petrosal. The region in question is damaged on both sides of the skull, so the presence or abence of grooves cannot be assessed properly.

The sulcus for the inferior petrosal sinus extends anteriorly from a point near the canaliculus cochleae, and the sulcus leads to a canal that borders the promontorium medially. The canal is not complete on either side of the skull, but it is clear that the inferior petrosal sinus took an intrapetrosal course in life (Supplementary Fig. 8c–d). A similar groove with partial canal was described previously for an isolated petrosal assigned to *Didelphodon vorax* (UCMP 52869), and the occupant of the sulcus was identified as a branch of the internal carotid artery at that time (Clemens[45], following Patterson[56]). However, the sulcus in NDGS 431 is in the same location and of similar morphology as the sulcus that transmits the inferior petrosal sinus in extant marsupials, such as *Didelphis virginiana*[57] (see also Archibald[58]), and a sulcus for the internal carotid artery leading to the anterior carotid foramen is present anterior and external to the tympanic bulla as described above (Supplementary Fig. 7). Thus, we identify the medial groove as the sulcus for the inferior petrosal sinus.

A broad and distinct sulcus that contributes to the posttemporal canal for the diploëtic artery and vein (arteria and vena diploetica magna) is observed lateral to the subarcuate fossa, extending in an anterior-posterior direction along the lateral surface of the petrosal (Supplementary Fig. 9a). A corresponding sulcus on the squamosal completes a canal between the two bones (Supplementary Fig. 7b). Anterior to the petrosal, the canal curves ventrally and splits into an anterior and a posterior branch. The posterior branch opens via the postglenoid foramen on the

posterior surface of the postglenoid process of the squamosal to transmit the postglenoid vein in life (Supplementary Fig. 9b–d). The anterior likely transmitted an emissary vein in life, which ultimately exited the skull through a nutrient foramen on the posterior wall of the temporal fossa.

Near the anterior terminus of the petrosal contribution of the posttemporal canal is a small foramen (Supplementary Fig. 9a) leading to a vascular canal that opens lateral to the tympanic opening of the facial nerve canal and at the posteromedial end of the external acoustic meatus, within the petrosal-squamosal suture. The canal splits internal to the ventral opening, sending one branch posteriorly and another much shorter and horizontal canal that extends medially towards the epitympanic recess. The extent of the posterior channel, which likely would have carried an emissary vein in life, cannot be determined on account of damage to the petrosal and posterior cranium. The horizontal canal opens lateral to the tympanic aperture of the facial canal in the approximate position of the prootic canal for the lateral head vein in other metatherians. As described above, there is no indication of a prootic sinus canal that typically extends in a posterodorsal-anteroventral direction in many therians[30,57,59-63]. The canal may be present but filled with matrix that is indistinguishable from fossil bone in the CT scans.

Bony Labyrinth—As with the petrosal, the chambers of the bony labyrinth of the inner ear are divided into two general regions – the anterior cochlea with the spiral organ of hearing and the posterior vestibular apparatus (including semicircular canals) with the organs of balance. Dimensions of bony labyrinth of NDGS 431 are provided in Supplementary Table 1. The cochlea at the anteroventral end of the bony labyrinth contributes less than half of the total inner ear volume (46.1%). The cochlea is better preserved on the right side of the skull, and the canal

completes just over one and three quarters turns (Supplementary Fig. 10). The degree of coiling measured here for *Didelphodon* (1.8 turns) is greater than that measured for any other Mesozoic therian described to date[64-69]. Although the apical turn of the cochlea is poorly preserved within the left petrosal (on account of damage to the ventral surface of the promontorium), it appears that the left cochlea coils to the same degree as the right. The cochlea has a low profile compared to more recent mammals[70] with an aspect ratio of 0.42 for the left cochlea. Neither the right nor the left cochlea is expanded at its apical end indicating a lagena, and no canals for lagenar nerves were observed.

Internal structures within the cochlea, such as the primary and secondary bony laminae to support the basilar membrane, as well as the Rosenthal's canal for the spiral nerve ganglion, were difficult to resolve in the CT data on account of the delicate nature of the structures. Likewise, presence of a cribriform plate for individual auditory nerve fibers within the internal auditory meatus could not be resolved in CT images, but likely are present given their occurrence in more basally diverging cladotherians[71]. Traces of the secondary bony lamina are visible on the endocasts as a faint groove along the radial (outer) wall of the cochlea, particularly in the left labyrinth (Supplementary Fig. 10a–c), but the robustness and total extent of the structure could not be determined. The bony canaliculus cochleae for the membranous perilymphatic duct is complete and undamaged on the right side of the skull and takes a straight course as it exits the cochlear cavity (Supplementary Fig. 10b, d). The left petrosal is fractured across the course of the canaliculus, giving the endocast of the canaliculus a bent appearance on that side of the skull (Supplementary Fig. 10a, c).

The bony vestibule is anterior-posteriorly elongate with no clear separation between the spherical and elliptical recesses (for the membranous saccule and utricle respectively). The posterior limb of the lateral semicircular canal does not open into the vestibule directly, but rather it joins with the lateral limb of the posterior canal to form a secondary common crus (Supplementary Fig. 10a, c). The posterior semicircular canal is not preserved on the right side of the skull, but a small portion of the right lateral canal and nearly all of the secondary common crus is present. The bony channel for the vestibular aqueduct exits the vestibule medial to the common crus (Supplementary Fig. 10c–d). The aqueduct extends dorsally and posteriorly as a slender canal before opening onto the endocranial surface of the petrosal along the rim of the subarcuate fossa (Supplementary Fig. 8a–b).

The angular relationships between the planes of ipsilateral (same side) semicircular canals could only be determined for the left bony labyrinth, owing to incomplete preservation of the right petrosal. The plane of the posterior semicircular canal forms roughly right angles with the anterior and lateral canals, and the anterior and lateral semicircular canal planes form a more acute angle (Supplementary Table 2). The three semicircular canals are more or less planar, although the central course of the anterior canal is slightly distorted, which gives the anterior canal an appearance of torsion (Supplementary Fig. 10c–d). The distortion is the result of preservation (the rim of the subarcuate fossa has been crushed and pushed in towards the center of the fossa in that region) rather than biological torsion of the canal from its plane.

An acute angle was measured between the right and left anterior semicircular canals, but other contralateral canal pairs (opposite sides) could not be determined. The right and left contralateral

anterior semicircular canals deviated from the three orthogonal planes by roughly the same degree, and an acute angle between the right anterior and left posterior synergistic semicircular canals (those that work against each other in a push-pull fashion; see ref. 72) was measured (see Supplementary Table 2). In an ideal physiological model, synergistic canals are parallel and contralateral canals are perpendicular[72], but the canals rarely are oriented in this fashion in natural biological systems[73]. The other synergistic canals are the left anterior (not preserved) and right posterior, and the right and left lateral semicircular canals (the left lateral is not preserved). The right and left contralateral anterior semicircular canals deviated from the three orthogonal planes by roughly the same degree (Supplementary Table 2).

The anterior semicircular canal is larger than either the lateral or posterior canals in terms of length of the slender (unampullated) portion of the canal and canal arc radius (Supplementary Table 1), but the cross-sectional diameter and volume of the lateral semicircular canal is larger than those measures for either the anterior or posterior canal. However, the difference might be the result of damage and the difficult estimation of the walls of the anterior canal along its midsection with similar matrix and bone contrasts in the CT scan images. The arc of the posterior semicircular canal approaches circularity (aspect ratio of 0.98), and the aspect ratios of the anterior and lateral semicircular canal arcs are lower and more elongate (aspect ratios of 0.71 and 0.83 respectively). Although neither the right nor the left anterior canal is complete or undamaged, the canal arc has reached its apex from the common crus proximal to the damaged regions and would not form a more circular circuit if undamaged.

Occiput—NDGS 431 (Supplementary Figs. 1b and 2b) preserves most of the dorsal half of the occiput, having lost the exoccipitals. In turn, the morphology of the paracondylar processes, occipital condyles, and the dimensions of the foramen magnum are unknown. The well-developed lambdoidal crest dorsally overhangs the occiput. The supraoccipital, which contacts the interparietal just ventral to the lambdoidal overhang, gently slopes posteroventrally toward a broken margin that has no clear sign of an incisura occipitalis or a dorsal margin of the foramen magnum. The posterior part and the right side of the basioccipital are fragmentary.

Dentition—Each premaxilla contains four subequal alveoli for single-rooted incisors (Supplementary Figs. 1e, 2g, 3d,e, and 4d), but only the left and right I1s are preserved in UWBM 94084. The I1s, which possess a main cusp and a posterior cuspule, are more robust and more laterally compressed than those of *Didelphis virginiana* and *Monodelphis brevicaudata*. The left and right I1s in UWBM 94084 extend 6.3 mm out of their alveoli (enamel-covered crown is 3.0 mm tall), and the roots are at least twice the length of the enamel-covered crown. They are comparable in length to those of *Didelphis* and *Monodelphis*. Unlike in *Monodelphis*, they do not clearly angle towards each other or make contact with each other at their crown apices[23]. The left I1 shows some slight, flat apical wear on the main cusp and it preserves a small cuspule posterior and internal to the main cusp. On the right I1, the apex of the main cusp is heavily worn, forming a mesially directed slope and exposing the pulp cavity; the small posterior cuspule is worn flat but is discernible. There are no marked differences in the sizes of incisor alveoli of UWBM 94084, but the incisor pattern in *Didelphodon vorax* may follow that in *Didelphis* and *Monodelphis*, wherein I1 is the tallest and largest (in diameter) and I2–4 are subequal to each other in height and size[23].

The alveolar row slopes dorsally into the premaxillary groove, which received the lower canine. In UWBM 94084 (Supplementary Figs. 3 and 4), right C1 is unworn except for slight apical wear and a narrow facet along the length of the crown on the mesial aspect, which was formed by attritional wear with the opposing lower canine. The crown is tall and robust (see Supplementary Table 9), not as laterally compressed as similarly sized canines in *Didelphis virginiana* and *Monodelphis brevicaudata*, recurved along its length, pointed at the apex, and bears a narrow shelf at the distal base of the crown. The left C1 is heavily worn along the mesial aspect of the crown, exposing the pulp cavity and reducing the height of the crown to less than half that of the right C1. The C1 root is broader than the crown and it extends posterodorsally into the maxilla to the level of the P3. The canines of NDGS 431 (Supplementary Figs. 1 and 2) are smaller but similarly show asymmetrical wear; the right C1 of NDGS 431 is heavily worn, whereas the left C1 of NDGS 431 is completely unworn.

The P1 of SCNHM VMMa 20 (Supplementary Figs. 5a–d and 6a–d) has a robust lingual root and what we interpret as a separate buccal root that has been broken at its base. MicroCT images of UWBM 94084 (Supplementary Fig. 3f) corroborate this P1 root morphology. In contrast, Lofgren[74] interpreted an edentulous maxillary fragment (UCMP 134795) as having a single, un-bifurcated P1 alveolus; thus, *Didelphodon vorax* either shows variability in P1 root morphology or the single alveolus of UCMP 134795 housed a two-rooted P1. The P1 has a bean-shaped crown that has a mesiolingual concavity to receive the distal shelf of C1 (Supplementary Figs. 5d and 6d). The apex of the main buccal cusp in NDGS 431 and SCNHM VMMa 20 is flat from slight wear. Ridges emanate from this cusp mesially and distally. The mesial ridge is shorter; and

in NDGS 431, it terminates mesially in two small cuspules, but those cuspules are not preserved in SCNHM VMMa 20. The longer, more gently sloping distal ridge also terminates distally with a cuspule. From that distal cuspule a ridge extends toward the lingual lobe, forming a shallow basin between the distal ridge and the distolingual ridge. Lingual to this shallow basin, the lingual lobe is gently sloping and has an inflated appearance.

The P2 is bilobate with a large buccal cusp and smaller lingual cusp that merge into flat, circular facets with wear (Supplementary Figs. 5d and 6d). Its mesial concavity receives the slightly convex distal aspect of P1. A short distal ridge does extend from the main buccal cusp to a distobuccal cuspule. A cuspidate distolingual ridge extends from this distobuccal cuspule to the lingual cusp, forming a basin that is shallower than that in P1. The lingual lobe is mesiodistally broad, symmetrical and has an inflated appearance that overhangs the crown cervix.

The P3 is rectangular in occlusal outline. In UWBM 94084 and to a lesser degree in UWBM 94500, the P3 crown is nearly worn flat to the cervix. In SCNHM VMMa 20 and NDGS 431, the P3s are relatively unworn and are taller than all other cheek teeth. The buccal cusp is tall with a prominent distal ridge that terminates in a cuspule; a distolingual ridge extends from that cuspule onto the broad, bulging lingual lobe. There is a less distinct mesial cusp and shorter, subtler mesial and mesiolingual ridges. In size, P3>P2>P1. The enamel on all of the premolars is vertically corrugated, particularly on the buccal and lingual faces of the main cusp. On P2 and P3 below the cervix, the lingual root has a roughened and bulging appearance, presumably from deposition of cement[75,76].

The upper molars match detailed descriptions of *Didelphodon vorax* from Clemens[45]. The revised dental formula for *D. vorax* is I4/i3 C1/c1 P3/p3 M4/m4. Dental measurements are in Supplementary Table 3.

Supplementary Note 2: Phylogenetic character list and scores for *Didelphodon*

Below is the character list and descriptions; characters 1–156 are from Rougier et al.[77] (appendix A), characters 157–162 are from Horovitz et al.[30], and characters 163–164 are new from this study. Scores for *Didelphodon* are listed below each character description with new and revised scores underlined. Changes to scores of other taxa are also noted in bold. We treated eleven characters as ordered (characters 1, 4, 7, 12, 14, 35, 36, 50, 51, 52, and 116); all others were unordered.

General Dentition (7 characters)

1. Number of premolars – five (0), four (1), three (2), or less than three (3). Ordered.

***Didelphodon* = 2**

2. Premolar cusp form – sharp, uninflated (0) or inflated, with apical wear strongly developed (1).

***Didelphodon* = 1**

3. Tall, trenchant premolar – in last premolar position (0), in penultimate premolar position (1), or absent (2). [Upper dentition considered when possible]

***Didelphodon* = 2**

4. Number of molars – more than four (0), four (1), or three (2). Ordered.

***Didelphodon* = 1**

5. Molar cusp form – sharp, gracile (0) or inflated, robust (1).

***Didelphodon* = 0**

6. Size of molars increasing posteriorly – absent (0), moderate posterior increase (1), or marked posterior increase (2). [All molars considered in lower jaw, and all but the last considered in upper jaw]

***Didelphodon* = 2**

7. Number of postcanine tooth families – eight or more (0), seven (1), or less than seven (2). Ordered.

***Didelphodon* = 1**

Upper Dentition (34 characters)

8. Number of upper incisors – five (0) or less than five (1).

***Didelphodon* = 1**

9. First upper incisor – enlarged, anteriorly projecting, separated from I2 by small diastema (0), subequal or smaller than remaining incisors, without diastema (1), or lost (2).

***Didelphodon* = 1**

10. Number of roots on upper canine – two (0) or one (1).

***Didelphodon* = 1**

11. First upper premolar – erect, without diastema (0), erect, with a short diastema (1), or procumbent, separated by diastema (2).

***Didelphodon* = 0**

12. Penultimate upper premolar protocone – absent (0), small lingual bulge (1), or with an enlarged basin (2). Ordered.

***Didelphodon* = 1**

13. Number of roots on penultimate upper premolar – two (0) or three (1).

***Didelphodon* = 0**

14. Last upper premolar – simple (0), complex, with small protocone (1), or molariform (2). Ordered.

***Didelphodon* = 1**

15. Upper molar shape – as long as wide, or longer (0) or wider than long (1).

***Didelphodon* = 1**

16. Upper molar outline in occlusal view – does (0) or does not (1) approach isosceles triangle.

***Didelphodon* = 1**

17. Stylar shelf – uniform in width, 50% or more of total transverse width (0), uniform in width, but less than 50% of total transverse width (1), slightly reduced labial to paracone (2), strongly reduced labial to paracone (3), or strongly reduced or absent (4). [penultimate molar considered when present]

***Didelphodon* = 2**

18. Metastylar area on penultimate upper molar – large (0) or reduced (1).

***Didelphodon* = 0**

19. Deep ectoflexus – present only on penultimate molar (0), on penultimate and preceding molar (1), or strongly reduced or absent (2).

***Didelphodon* = 1**

20. Stylar cusp A – distinct, but smaller than B (0), subequal to larger than B (1), or very small to indistinct (2). [penultimate molar considered when available]

***Didelphodon* = 0&2**

21. Preparastyle – absent (0) or present (1).

***Didelphodon* = 0**

22. Stylar cusp B size relative to paracone – smaller but distinct (0), vestigial to absent (1), or subequal (2).

***Didelphodon* = 2**

23. Stylar cusp C – absent (0) or present (1).

***Didelphodon* = 0**

24. Stylar cusp D – absent (0), smaller or subequal to B (1), or larger than B (2).

***Didelphodon* = 1**

25. Stylar cusp E – directly lingual to D or D position (0), distal to D (1), or small to indistinct (2).

***Didelphodon* = 2**

26. Preparacingulum – absent (0), interrupted between stylar margin and paraconule (1), or continuous (2). [penultimate molar considered when available]

***Didelphodon* = 2**

27. Metacone size relative to paracone – noticeably smaller (0), slightly smaller (1), or subequal to larger (2).

***Didelphodon* = 2**

28. Metacone position relative to paracone – labial (0), approximately at same level (1), or lingual (2).

***Didelphodon* = 2**

29. Metacone and paracone shape – conical (0) or subtriangular, with labial face flat (1).

***Didelphodon* = 0**

30. Metacone and paracone bases – adjoined (0) or separated (1).

***Didelphodon* = 1**

31. Centrocrista – straight (0) or V-shaped (1).

***Didelphodon* = 0**

32. Salient postmetacrista – weakly developed (0) or strongly developed, with paraconid enlarged and metaconid reduced on lower molars (1).

***Didelphodon* = 1**

33. Preprotocrista – does not (0) or does (1) extend labially past base of paracone (double rank prevallum/postvallid shearing).

***Didelphodon* = 1**

34. Postprotocrista – does not (0) or does (1) extend labially past base of metacone (double rank prevallum/postvallid shearing).

***Didelphodon* = 0**

35. Conules – absent (0), small, without cristae (1), or strong, labially placed, with wing-like cristae (2). Ordered.

***Didelphodon* = 2**

36. Protocone on upper molars – lacking (0), small, without trigon basin (1), small, with distinct trigon basin (2), somewhat expanded anteroposteriorly (3), or with posterior portion expanded (4). Ordered.

***Didelphodon* = 4**

37. Procumbent protocone – absent (0) or present (1).

***Didelphodon* = 1**

38. Protocone height – low (0) or tall, approaching para- and/ or metacone height (1).

***Didelphodon* = 1**

39. Protocingula – absent (0) or pre- and/or postcingulum present (1).

***Didelphodon* = 0**

40. Lingual root position – supporting paracone (0) or supporting trigon (1).

***Didelphodon* = 1**

41. Last upper molar width relative to penultimate upper molar – subequal (0) or smaller (1).

***Didelphodon* = 0**

Lower Dentition (22 characters)

42. Number of lower incisors – four (0) or less than four (1).

***Didelphodon* = 1**

43. Staggered lower incisor – absent (0) or present (1).

***Didelphodon* = 1**

44. Roots on lower canine – biradiculated (0) or uniradiculated (1).

***Didelphodon* = 1**

45. First lower premolar – oriented in line with jaw axis (0) or oblique (1).

***Didelphodon* = 1**

46. Second lower premolar – smaller than third premolar (0) or larger (1).

***Didelphodon* = 0**

47. Last lower premolar – simple (0), complex, with a partial trigonid and/or talonid (1), or molariform (2). Ordered.

***Didelphodon* = 0**

48. Trigonid configuration – open, with paraconid anteromedial (0), more acute, with paraconid more posteriorly placed (1), or anteroposteriorly compressed (2).

***Didelphodon* = 2**

49. Lower molar talonid – small heel (0) or multicuspidated basin (1).

***Didelphodon* = 1**

50. Talonid width relative to trigonid – very narrow, subequal to base of metaconid, developed lingually (0), narrower (1), or subequal to wider (2). Ordered.

***Didelphodon* = 2**

51. Lower molar cristid obliqua – incomplete, with distal metacristid present (0), complete, attaching below notch in metacristid (1), or complete, labially placed, at base of protoconid (2). Ordered.

***Didelphodon* = 2**

52. Hypoconulid – absent (0), in posteromedial position (1), or lingually placed and ‘‘twinned’’ with entoconid (2). Ordered.

***Didelphodon* = 2**

53. Hypoconulid of last molar – short and erect (0) or tall and sharply recurved (1).

***Didelphodon* = 0**

54. Entoconid – absent (0), smaller than (1), or subequal to larger than hypoconid and/or hypoconulid (2).

***Didelphodon* = 2**

55. Labial postcingulid – absent (0) or present (1).

***Didelphodon* = 1**

56. Paraconid and metaconid – metaconid at extreme lingual margin (0) or aligned (1).

***Didelphodon* = 1**

57. Metacristid orientation to lower jaw axis – oblique (0) or transverse (1).

***Didelphodon* = 1**

58. First lower molar paraconid, low and confluent with precingulid – absent (0) or present (1).

***Didelphodon* = 0**

59. Protoconid height – tallest cusp on trigonid (0) or subequal to para- and/or metaconid (1).

***Didelphodon* = 1**

60. Paraconid height relative to metaconid – taller (0), subequal (1), or shorter (2). [molars other than the first considered when available]

***Didelphodon* = 0**

61. Last lower molar size relative to penultimate lower molar – subequal (0) or smaller or lost (1).

***Didelphodon* = 0**

62. Rotation of last lower molar during eruption – absent (0) or present (1).

***Didelphodon* = ?**

63. Space between last lower molar and coronoid process – present (0) or absent (1).

***Didelphodon* = 0**

Tooth Replacement (3 characters)

64. Deciduous incisors – present (0) or absent (1).

***Didelphodon* = ?**

65. Deciduous canine – present (0) or absent (1).

***Didelphodon* = ?**

66. Replacement of dP1/dp1 and dP2/dp2 – present (0) or absent (1).

***Didelphodon* = 1**

Mandible (11 characters)

67. Masseteric fossa – restricted dorsally by crest reaching condyle (0) or extended ventrally to lower margin of dentary (1).

***Didelphodon* = 1**

68. Posterior shelf of masseteric fossa – absent (0) or present (1).

***Didelphodon* = 1**

69. Convex ventral margin behind tooth row continuous to condyle – absent (0) or present (1).

***Didelphodon* = 1**

70. Labial mandibular foramen – present (0) or absent (1).

***Didelphodon* = 1**

71. Condyle shape – ovoid (0) or cylindrical (1).

***Didelphodon* = 1**

72. Condyle position relative to tooth row – above (0) or very high (1)

***Didelphodon* = 0**

73. Lower jaw angle – posteriorly directed (0), medially inflected (1), or posteroventrally directed (2).

***Didelphodon* = 1**

74. Mandibular foramen – below (0) or posterior to anterior edge of coronoid process (1).

***Didelphodon* = 1**

75. Meckelian groove – present (0) or absent (1).

***Didelphodon* = 1**

76. ''Coronoid'' facet – present (0) or absent (1).

***Didelphodon* = 1**

77. Two large mental foramen, one under second and third premolar and the other under first and second molar – absent (0) or present (1).

***Didelphodon* = 1**

Cranium (79 characters)

78. Septomaxilla – present (0) or absent (1).

***Didelphodon* = 1**

79. Premaxilla, palatal process – does not (0) or does reach nearly or to canine alveolus (1).

***Didelphodon* = 1; *Pucadelphys* and *Andinodelphys* were scored 0 on the basis of Horovitz et al.[30] and our own observations.**

80. Premaxilla, facial process – does not (0) or does reach the nasal (1).

***Didelphodon* = 1**

81. Lateral margin of paracanine fossa – formed by maxilla (0) or maxilla and premaxilla (1).

***Didelphodon* = 1**

82. Exit(s) of infraorbital canal – multiple (0) or single (1).

***Didelphodon* = 1**

83. Flaring of cheeks behind infraorbital foramen, as seen in ventral view – present (0) or absent (1).

***Didelphodon* = 1**

84. Naso-frontal suture with medial process of frontals wedged between nasals – present (0) or absent (1).

***Didelphodon* = 1**

85. Nasal foramina – present (0) or absent (1).

***Didelphodon* = 1**

86. Frontal-maxillary contact – absent (0) or present (1).

***Didelphodon* = ?**

87. Lacrimal tubercle – present (0) or absent (1).

***Didelphodon* = ?**

88. Lacrimal foramen exposed on face – present (0) or absent (1).

***Didelphodon* = ?**

89. Lacrimal foramen number – double (0) or single (1).

***Didelphodon* = ?**

90. Preorbital length relative to postorbital length – two-thirds or more (0) or less than two-thirds (1).

***Didelphodon* = 1**

91. Maxillary-jugal contact bifurcated – absent (0) or present (1).

***Didelphodon* = 0**

92. Zygomatic arch – stout (0) or delicate (1).

***Didelphodon* = 0**

93. Palatal vacuities – absent (0) or present (1).

***Didelphodon* = 1; *Andinodelphys* was scored 0 on the basis of Horovitz et al.[30] and our own observations.**

94. Palatal expansion behind last molar – absent (0) or present (1).

***Didelphodon* = 1**

95. Postpalatine torus – absent (0) or present (1).

***Didelphodon* = 1**

96. Palate and basicranium at same level, connected by broad choanal ridges – absent (0) or present (1).

***Didelphodon* = 0**

97. Minor palatine (postpalatine) foramen – small (0) or large, with thin, posterior bony bridge (1).

***Didelphodon* = 0**

98. Palatine reaches infraorbital canal – present (0) or absent (1).

***Didelphodon* = ?**

99. Pterygoids contact on midline – present (0) or absent (1).

***Didelphodon* = 1**

100. Pterygopalatine crests – present (0) or absent (1).

***Didelphodon* = 1**

101. Ectopterygoid process of alisphenoid – absent (0) or present (1).

***Didelphodon* = 0**

102. Optic foramen – absent (0) or present (1).

***Didelphodon* = ?; *Mayulestes* was scored ? on the basis of Horovitz et al.[30] indicating that this feature is not observable.**

103. Orbitotemporal canal – present (0) or absent (1).

***Didelphodon* = ?**

104. Transverse canal – absent (0) or present (1).

***Didelphodon* = ?**

105. Carotid foramen – within basisphenoid (0) or between basisphenoid and petrosal (1).

***Didelphodon* = 1**

106. Dorsum sellae – tall (0) or low (1).

***Didelphodon* = ?; The 'Gurlin Tsav skull' was scored ? indicating that this feature is not observable.**

107. Alisphenoid canal – absent (0) or present (1).

***Didelphodon* = ?**

108. Anterior lamina exposure on lateral braincase wall – present (0), rudimentary (1), or absent (2).

***Didelphodon* = 2**

109. Cavum epiptericum – floored by petrosal (0), petrosal and alisphenoid (1), primarily or exclusively by alisphenoid (2), or primarily open as piriform fenestra (3).

***Didelphodon* = 2**

110. Exit for maxillary nerve relative to alisphenoid – behind (0) or within or in front (1).

***Didelphodon* = ?**

111. Foramen ovale composition – in petrosal (anterior lamina) (0), between petrosal and alisphenoid (1), in alisphenoid or between alisphenoid and squamosal (2).

***Didelphodon* = 2**

112. Foramen ovale – on lateral wall of braincase (0) or on ventral surface of skull (1).

***Didelphodon* = 1**

113. Squama of squamosal – absent (0) or present (1).

***Didelphodon* = 1**

114. Position of jaw articulation relative to fenestra vestibuli – at same level (0) or in front (1).

***Didelphodon* = 1**

115. Glenoid fossa shape – concave, open anteriorly (0) or trough-like (1).

***Didelphodon* = 1**

116. Glenoid process of jugal – present, with articular facet (0), present, without facet (1), or absent (2). Ordered.

***Didelphodon* = 0**

117. Glenoid process of alisphenoid – absent (0) or present (1).

***Didelphodon* = 0**

118. Postglenoid process – absent (0) or present (1).

***Didelphodon* = 1**

119. Postglenoid-suprameatal vascular system – absent (0), present, below squamosal crest (1), or present, above squamosal crest (2).

***Didelphodon* = 0**

120. Postglenoid foramen – absent (0), present, behind postglenoid process (1), or present, medial to postglenoid process (2).

***Didelphodon* = 1**

121. Alisphenoid tympanic process – absent (0) or present (1).

***Didelphodon* = 1**

122. Epitympanic wing medial to promontorium – absent (0), flat (1), undulated (2), or confluent with bulla (3).

***Didelphodon* = 2**

123. Tympanic aperture of hiatus Fallopii – in roof through petrosal (0), at anterior edge of petrosal (1), or absent (2).

***Didelphodon* = 1**

124. Prootic canal – long and vertical (0), short and vertical (1), short and horizontal (2), or absent (3).

***Didelphodon* = 2**

125. Position of sulcus for anterior distributary of transverse sinus relative to subarcuate fossa – anterolateral (0) or posterolateral (1).

***Didelphodon* = 1**

126. Lateral flange – parallels length of promontorium (0), restricted to posterolateral corner (1), or greatly reduced or absent (2).

***Didelphodon* = 1**

127. Stapedial ratio – rounded, less than 1.8 (0) or elliptical, more than 1.8 (1).

***Didelphodon* = ?**

128. Complete wall separating cavum supracochleare from cavum epiptericum – absent (0) or present (1).

***Didelphodon* = 1; *Andinodelphys* was scored 1 on the basis of Horovitz et al.[30].**

129. Coiling of cochlea – less than 360° (0) or 360° or greater (1).

***Didelphodon* = 1**

130. Rostral tympanic process of petrosal, on posteromedial aspect of promontorium – absent or low ridge (0), tall ridge, occasionally contacting ectotympanic (1).

***Didelphodon* = 0**

131. Paroccipital process (sensu Wible and Hopson[78]) orientation and shape – vertical (0), slanted, projecting anteroventrally as flange towards back of promontorium (1), or indistinct to absent (2).

***Didelphodon* = 0**

132. Caudal tympanic process of petrosal development – tall wall behind postpromontorial recess (0), tall wall decreasing in height markedly medially (1), or notched between stylomastoid notch and jugular foramen (2).

***Didelphodon* = 1**

133. Crista interfenestralis and caudal tympanic process of the petrosal connected by curved ridge – absent (0) or present (1).

***Didelphodon* = 0**

134. ''Tympanic process'' – absent (0) or present (1).

***Didelphodon* = 0**

135. Tall paracondylar (''paroccipital'') process of exoccipital (sensu Evans and Christensen[79]) – absent (0) or present (1).

***Didelphodon* = ?**

136. Rear margin of auditory region – marked by a steep wall (0) or extended onto a flat surface (1).

***Didelphodon* = 0**

137. Fossa incudis – continuous with (0) or separated from (1) epitympanic recess.

***Didelphodon* = 1**

138. Epitympanic recess – with small contribution to posterolateral wall by squamosal (0) or with extensive contribution to lateral wall by squamosal (1).

***Didelphodon* = 1**

139. Stapedius fossa – twice the size of fenestra vestibuli (0) or small and shallow (1).

***Didelphodon* = 1**

140. Hypotympanic sinus – absent (0), formed by squamosal, petrosal, and alisphenoid (1), or formed by alisphenoid and petrosal (2).

***Didelphodon* = 2**

141. Medial process of squamosal in tympanic cavity – absent (0) or present (1).

***Didelphodon* = 0**

142. Ectotympanic – ring-like (0), fusiform (1), or expanded (2).

***Didelphodon* = ?**

143. Foramina for temporal rami – on petrosal (0), on parietal and/or squama of squamosal (1), or absent (2).

***Didelphodon* = 2**

144. Posttemporal canal – large (0), small (1), or absent (2).

***Didelphodon* = 0; *Pucadelphys* and *Andinodelphys* were scored 2 on the basis of Horovitz et al.[30].**

145. Foramen for ramus superior of stapedial artery – on petrosal (0), on petrosal-squamosal suture (1), or absent (2).

***Didelphodon* = 2**

146. Transpromontorial sulcus – present (0) or absent (1).

***Didelphodon* = 1**

147. Sulcus for stapedial artery – present (0) or absent (1).

***Didelphodon* = 1**

148. Deep groove for internal carotid artery excavated on anterior pole of promontorium – absent (0) or present (1).

***Didelphodon* = 0**

149. Jugular foramen size relative to fenestra cochleae – subequal (0) or larger (1).

***Didelphodon* = ?**

150. Jugular foramen – confluent with (0) or separated from (1) opening for inferior petrosal sinus.

***Didelphodon* = ?**

151. Inferior petrosal sinus – intrapetrosal (0), between petrosal, basisphenoid, and basioccipital (1), or endocranial (2).

***Didelphodon* = 0**

152. Ascending canal – intramural (0), intracranial (1), or absent (2).

***Didelphodon* = 2**

153. Internal acoustic meatus – deep, with thick prefacial commissure (0) or shallow, with thin prefacial commissure (1).

***Didelphodon* = 0; *Andinodelphys* was scored 1 on the basis of Horovitz et al.[30].**

154. Mastoid-squamosal fusion – absent (0) or present (1).

***Didelphodon* = 0**

155. Interparietal – absent (0) or present (1).

***Didelphodon* = 1**

156. Dorsal margin of foramen magnum – formed by exoccipitals (0) or by exoccipitals and supraoccipital (1).

***Didelphodon* = ?**

From Horovitz et al.[30] (6 characters)

157. Upper incisor arcade shape*– U-shape (0), broad V-shape (1), or long, narrow V-shape (2). (Ch. 158 of Horovitz et al.[30] but unordered)

***Didelphodon* = 0**

158. Posterior-most point of premaxillo-nasal contact – anterior or at the canine (0) or posterior to the canine (1). (Ch. 178 of Horovitz et al.[30])

***Didelphodon* = 1**

159. Fossa subarcuata –smaller than its aperture (i.e., conical shape) (0) or larger than its aperture (i.e., spherical shape) (1). (Ch. 221 of Horovitz et al.[30])

***Didelphodon* = 1**

160. Deep and large fossa for the tensor tympani muscle excavated on the anterolateral aspect of promontorium, creating a battered ventral surface of the promontorium – absent (0) or present (1). (Ch. 229 of Horovitz et al.[30])

***Didelphodon* = 0**

161. Broad shelf of bone surrounding fenestra cochleae and making a separation between it and aqueductus cochleae – absent (0) or present (1). (Ch. 233 of Horovitz et al.[30])

***Didelphodon* = 0**

162. Mastoid exposure – large (0), narrow (1), or reduced pars mastoidea, internal to the braincase and wedged between the squamosal and exoccipital (2). (Ch. 239 of Horovitz et al.[30])

***Didelphodon* = 0**

New characters (2 characters)

163. Internarial process of the premaxilla – absent (0) or present (1).

***Didelphodon* = 1; The 'Gurlin Tsav skull' = 1**

164. Anterior-most extent of the nasal – anterior to or at the canine (0) or posterior or to the canine (1).

***Didelphodon* = 1**; **The 'Gurlin Tsav skull' = 1**

Supplementary Note 3: TNT output from phylogenetic analysis

```
Reading from \\VBOXSVR\05-Phylogenetic_analysis\TNT data
matrix\Didelphodon_Wilson_etal_Sept012016.tnt
Matrix (164x48, 16 states). Memory required for data:   0.16 Mbytes
Space for 10000 trees in memoryRandom seed is 1
0 trees in memory
Repl. Algor.     Tree        Score       Best Score   Time       Rearrangs.
499   FUSE       5           ------      601          0:03:46
4,204,530,888
Completed search.
Total rearrangements examined: 4,204,530,888.
No target score defined.  Best score hit 500 times.
Best score: 601.  18 trees retained.
Time 226.24 secs.
```

```
Strict consensus of 18 trees (0 taxa excluded)

   ┌0— 'Amphitherium_Dryolestidae'
48─┤   ┌1— Peramus
   └49─┤   ┌3— Kielantherium
       └51─┤   ┌2— Vincelestes
           └50─┤   ┌4— Potamotelses
               └52─┤   ┌5— Kermackia
                   └53─┤                ┌44- Aspanlestes
                       │            ┌80─┤   ┌40- Bobolestes
                       │            │   └79─┤
                       │            │       └39- Prokennalestes
                       │        ┌81─┤   ┌42- Kennalestes
                       │        │   └82─┼41- Asioryctes
                       │        │       └83─┬45- Leptictids
                       │        │           └43- Zalambdalestes
                       └55─┤    ┌6— Holoclemensia
                           │    │       ┌7— Pappotherium
                           │    │   ┌56─┤   ┌12- Sulestes
                           │    │   │   └61─┼11- Oklatheridium
                           │    │   │       │   ┌10- Tsagandelta
                           │    │   │       └60─┤   ┌15- Lotheridium
                           │    │   │           └59─┤   ┌9— Deltatheroides
                           │    │   │               └58─┼8— Deltatheridium
                           │    │   │                   └62─┬14- Nanocuris
                           └54─┤    │                       └13- Atokatheridium
                                └57─┤       ┌16- Gurlin_Tsav_Skull
                                    │   ┌63─┤   ┌31- Borhyaenids
                                    │   │   └72─┤   ┌30- Mayulestes
                                    │   │       └71─┤   ┌34- Jaskhadelphys
                                    │   │           └74─┤   ┌33- Andinodelphys
                                    │   │               └73─┤
                                    │   │                   └32- Pucadelphys
                                    └64─┤   ┌29- Asiatherium
                                        │   │   ┌27- Turgidodon
                                        │   │   ├26- Alphadon
                                        │   │   ├25- Albertatherium
                                        └67─┤   ├21- Aenigmadelphys
                                            │   ├19- Anchistodelphys
                                            │   ├70─┬28- Glasbius
                                            │   │   └24- Pediomys
                                            └66─┤
                                                ├68─┬20- Iugomortiferum
                                                │   └18- Kokopellia
                                                │   ┌17- Pariadens
                                                ├65─┤   ┌23- Eodelphis
                                                │   └69─┤
                                                │       └22- Didelphodon
                                                │   ┌38- Dromiciops
                                                └78─┤       ┌47- 'Mimo_Peradectes'
                                                    │   ┌84─┤
                                                    │   │   └46- Herpetotherium
                                                    └77─┤   ┌37- Dasyurids
                                                        └76─┤   ┌36- Didelphis
                                                            └75─┤
                                                                └35- Marmosa
```

Supplementary Note 4: Templeton tests of Alternative Topologies #1 and #2

We submitted our data matrix with a single positive force constraint (Alternative Topology #1: 'Gurlin Tsav skull', SA stem marsupialiforms, and Marsupialia) to a traditional search in TNT (version 1.1[80]), using 100 replicates and 10,000 saved trees per replication. We obtained 28 EPTs with tree length = 605. The consensus tree is shown below.

```
Tree 1:

  ┌0— 'Amphitherium_Dryolestidae'
48┤  ┌1— Peramus
  └49┤  ┌3— Kielantherium
     └51┤  ┌2— Vincelestes
        └50┤  ┌4— Potamotelses
           └52┤  ┌5— Kermackia
              └53┤        ┌44- Aspanlestes
                 │     ┌77┤  ┌40- Bobolestes
                 │     │  └76┤
                 │     │     └39- Prokennalestes
                 │  ┌78┤  ┌42- Kennalestes
                 │  │  └79┤41- Asioryctes
                 │  │     │  ┌45- Leptictids
                 │  │     └80┤
                 │  │        └43- Zalambdalestes
                 └55┤  ┌6— Holoclemensia
                    │  │     ┌7— Pappotherium
                    │  │  ┌56┤  ┌12- Sulestes
                    │  │  │  └61┤11- Oklatheridium
                    │  │  │     │  ┌10- Tsagandelta
                    │  │  │     └60┤  ┌15- Lotheridium
                    └54┤  │        └59┤  ┌9— Deltatheroides
                       │  │           └58┤8— Deltatheridium
                       │  │              │  ┌14- Nanocuris
                       │  │              └62┤
                       │  │                 └13- Atokatheridium
                       └57┤  ┌29- Asiatherium
                          │  │  ┌27- Turgidodon
                          │  │  ┤26- Alphadon
                          │  │  ┤25- Albertatherium
                          │  │  ┤21- Aenigmadelphys
                          └68┤  ┤20- Iugomortiferum
                             │  ┤19- Anchistodelphys
                             │  ┤18- Kokopellia
                             │  │  ┌28- Glasbius
                             └67┤71┤
                                │  └24- Pediomys
                                │  ┌17- Pariadens
                                ┤69┤  ┌23- Eodelphis
                                │  └70┤
                                │     └22- Didelphodon
                                │  ┌47- 'Mimo_Peradectes'
                                │  ┤46- Herpetotherium
                                │  ┤38- Dromiciops
                                │  │  ┌37- Dasyurids
                                │  ┤75┤  ┌36- Didelphis
                                │  │  └74┤
                                └66┤     └35- Marmosa
                                   │     ┌34- Jaskhadelphys
                                   │  ┌73┤  ┌33- Andinodelphys
                                   │  │  └72┤
                                   │  │     └32- Pucadelphys
                                   └65┤  ┌30- Mayulestes
                                      └64┤  ┌31- Borhyaenids
                                         └63┤
                                            └16- Gurlin_Tsav_Skull
```

We then ran a Templeton test to compare the consensus tree without constraints (our original tree) versus the above tree with the Alternative Topology #1 ('Gurlin Tsav skull' + SA stem marsupialiforms + Marsupialia). The following is the output.

```
Reading from \\VBOXSVR\05-Phylogenetic_analysis\TNT data
matrix\templeton.run
Running \\VBOXSVR\05-Phylogenetic_analysis\TNT data matrix\templeton.run
(with no arguments)
Macro language is OFF
15 loops and 40000 user variables
Macro language is ON
Macros: 1641.7 Kb in use, 358.2 Kb free
Templeton test of first tree in memory against second tree
Rank Difference Rankscore
```

```
1 -1.000000 -13.500000
2 1 13.500000
3 1 13.500000
4 1 13.500000
5 1 13.500000
6 1 13.500000
7 -1.000000 -13.500000
8 -1.000000 -13.500000
9 1 13.500000
10 1 13.500000
11 1 13.500000
12 1 13.500000
13 1 13.500000
14 1 13.500000
15 1 13.500000
16 1 13.500000
17 1 13.500000
18 1 13.500000
19 1 13.500000
20 1 13.500000
21 -1.000000 -13.500000
22 1 13.500000
23 1 13.500000
24 1 13.500000
25 -1.000000 -13.500000
26 -1.000000 -13.500000
27 2 28.500000
28 2 28.500000
29 2 28.500000
30 2 28.500000
Sum of negative ranks 81
Number of non-zero scores 30
Critical value is 151 for 5 percent, 137 for 2.5 percent, and 120 for 1
percent
Significant at the 1 percent level
```

We submitted our data matrix with a single positive forced constraint (Alternative Topology #2: SA stem marsupialiforms and Marsupialia) to a traditional search in TNT (version 1.1[80]), using 100 replicates and 10,000 saved trees per replication. We obtained 22 EPTs with tree length = 603. The consensus tree is shown below.

```
Tree 0:

  ┌0— 'Amphitherium_Dryolestidae'
48┤ ┌1— Peramus
  └49┤ ┌3— Kielantherium
     └51┤ ┌2— Vincelestes
        └50┤ ┌4— Potamotelses
           └52┤ ┌5— Kermackia
              │ │        ┌44- Aspanlestes
              │ │     ┌80┤ ┌40- Bobolestes
              │ │     │  └79┤
              │ │     │     └39- Prokennalestes
              └53┤  ┌81┤  ┌42- Kennalestes
                 │  │  └82┼41- Asiorcytes
                 │  │     └83┤ ┌45- Leptictids
                 │  │        └43- Zalambdalestes
                 └55┤  ┌6— Holoclemensia
                    │  │     ┌7— Pappotherium
                    │  │  ┌56┤ ┌12- Sulestes
                    │  │  │  └61┼11- Oklatheridium
                    │  │  │     └60┤ ┌10- Tsagandelta
                    │  │  │        └59┤ ┌15- Lotheridium
                    │  │  │           └58┤ ┌9— Deltatheroides
                    │  │  │              └─┼8— Deltatheridium
                    │  │  │                └62┤ ┌14- Nanocuris
                    │  │  │                   └13- Atokatheridium
                    └54┤  └57┤  ┌29- Asiatherium
                       │     │  │ ┌27- Turgidodon
                       │     │  │ ├26- Alphadon
                       │     │  │ ├25- Albertatherium
                       │     │  │ ├21- Aenigmadelphys
                       │     └67┤ ├20- Iugomortiferum
                       │        │ ├19- Anchistodelphys
                       │        │ ├18- Kokopellia
                       │        │ │  ┌69┤ ┌28- Glasbius
                       │        │ │  │  └24- Pediomys
                       │        └66┼65┤  ┌17- Pariadens
                       │          │  └64┤ ┌16- Gurlin_Tsav_Skull
                       │          │     └63┤ ┌23- Eodelphis
                       │          │        └68┤
                       │          │           └22- Didelphodon
                       │          │     ┌70┤ ┌31- Borhyaenids
                       │          │     │  └30- Mayulestes
                       │          │  ┌71┤  ┌34- Jaskhadelphys
                       │          │  │  └74┤ ┌33- Andinodelphys
                       │          │  │     └73┤
                       │          │  │        └32- Pucadelphys
                       │          └72┤  ┌38- Dromiciops
                       │             └78┤  ┌84┤ ┌47- 'Mimo_Peradectes'
                       │                └77┤  └46- Herpetotherium
                       │                   └76┤ ┌37- Dasyurids
                       │                      └75┤ ┌36- Didelphis
                       │                         └35- Marmosa
```

We then ran a Templeton test to compare the consensus tree without constraints (our original tree) versus the above tree with the Alternative Topology #2 (SA stem marsupialiforms + Marsupialia). The following is the output.

```
Reading from \\VBOXSVR\05-Phylogenetic_analysis\TNT data
matrix\templeton.run
```

```
Running \\VBOXSVR\05-Phylogenetic_analysis\TNT data matrix\templeton.run
(with no arguments)
Macro language is OFF
15 loops and 40000 user variables
Macro language is ON
Macros: 1641.7 Kb in use, 358.2 Kb free
Templeton test of first tree in memory against second tree
Rank Difference Rankscore
1 1 17
2 -1.000000 -17.000000
3 -1.000000 -17.000000
4 -1.000000 -17.000000
5 -1.000000 -17.000000
6 -1.000000 -17.000000
7 1 17
8 -1.000000 -17.000000
9 1 17
10 1 17
11 -1.000000 -17.000000
12 -1.000000 -17.000000
13 -1.000000 -17.000000
14 -1.000000 -17.000000
15 1 17
16 -1.000000 -17.000000
17 -1.000000 -17.000000
18 -1.000000 -17.000000
19 1 17
20 -1.000000 -17.000000
21 1 17
22 1 17
23 1 17
24 1 17
25 1 17
26 -1.000000 -17.000000
27 -1.000000 -17.000000
28 1 17
29 -1.000000 -17.000000
30 -1.000000 -17.000000
31 1 17
32 1 17
33 1 17
Sum of negative ranks 306
Number of non-zero scores 33
Critical value is 187 for 5 percent, 170 for 2.5 percent, and 151 for 1
percent
Not significant
```

Results from the Templeton tests indicate that our strict consensus tree is significantly better than Alternative Toplogy #1 (‘Gurlin Tsav skull’ + SA stem marsupialiforms + Marsupialia) but is not significantly better or worse than Alternative Topology #2 (SA stem marsupialiforms +

Marsupialia). That is, topologies in which the SA stem marsupialiforms are sister to Marsupialia are not significantly worse than our strict consensus tree topology in which Late Cretaceous NA stem marsupialiforms are sister to Marsupialia. However, because Alternative Topology #2 is two steps longer than our strict consensus tree topology, our strict consensus serves as the working phylogenetic hypothesis that should be further tested with additional fossils. It is noteworthy that in Alternative Topology #2 the 'Gurlin Tsav skull' is sister taxon to *Eodelphis* + *Didelphodon*, a relationship previously suggested in Rougier et al.[32] and reflective of the morphological similarities with *Didelphodon vorax* noted in our description. Unfortunately, the 'Gurlin Tsav skull' has not yet been formally described or comprehensively illustrated. See Szalay and Trofimov[53] (fig. 22) for the only published photographs. Thus, although a close relationship between these two taxa is plausible, we caution that a robust test of the phylogenetic relationships of the 'Gurlin Tsav skull' to other metatherians must await comprehensive and formal description of that fossil.

Supplementary References

1. Myers, T. J. Prediction of marsupial body mass. *Australian Journal of Zoology* **49**, 99-118 (2001).
2. Meers, M. B. Maximum bite force and prey size of *Tyrannosaurus rex* and their relationships to the inference of feeding behavior. *Historical Biology* **16**, 1-12 (2002).
3. Thomason, J. Cranial strength in relation to estimated biting forces in some mammals. *Canadian Journal of Zoology* **69**, 2326-2333 (1991).

4. Wroe, S., McHenry, C. & Thomason, J. Bite club: comparative bite force in big biting mammals and the prediction of predatory behaviour in fossil taxa. *Proceedings of the Royal Society of London B: Biological Sciences* **272**, 619-625 (2005).

5. Valkenburgh, B. V. & Ruff, C. Canine tooth strength and killing behaviour in large carnivores. *Journal of Zoology* **212**, 379-397 (1987).

6. Therrien, F. Mandibular force profiles of extant carnivorans and implications for the feeding behaviour of extinct predators. *Journal of Zoology* **267**, 249-270 (2005).

7. Nowak, R. M. *Walker's marsupials of the world*. (JHU Press, 2005).

8. Castro-Arellano, I., Zarza, H. & Medellin, R. A. *Philander opossum*. *Mammalian Species* **638**, 1-8 (2000).

9. Poglayen-Neuwall, I. & Toweill, D. E. *Bassariscus astutus*. *Mammalian Species* **327**, 1-8 (1988).

10. Gompper, M. E. *Nasua narica*. *Mammalian Species* **487**, 1-10 (1995).

11. Koontz, F. W. & Roeper, N. J. *Elephantulus rufescens*. *Mammalian Species* **204**, 1-5 (1983).

12. Woollard, P., Vestjens, W. & MacLean, L. The ecology of the eastern water rat *Hydromys chrysogaster* at Griffith, NSW: food and feeding habits. *Wildlife Research* **5**, 59-73 (1978).

13. Larivière, S. & Walton, L. R. *Lontra canadensis*. *Mammalian Species* **587**, 1-8 (1998).

14. Strahan, R. *The Mammals of Australia*. (Reed Books, 1995).

15. Lotze, J.-H. & Anderson, S. *Procyon lotor*. *Mammalian Species* **119**, 1-8 (1979).

16. Willner, G. R., Feldhamer, G. A., Zueker, E. E. & Chapman, J. A. *Ondatra zibethicus*. *Mammalian Species Archive* **141**, 1-8 (1980).

17. Tory, M., May, T., Keane, P. & Bennett, A. Mycophagy in small mammals: A comparison of the occurrence and diversity of hypogeal fungi in the diet of the long‑nosed potoroo *Potorous tridactylus* and the bush rat *Rattus fuscipes* from southwestern Victoria, Australia. *Australian Journal of Ecology* **22**, 460-470 (1997).

18. Bennett, A. & Baxter, B. Diet of the long-nosed potoroo, Potorous-Tridactylus (Marsupialia, Potoroidae), in southwestern Victoria. *Wildlife Research* **16**, 263-271 (1989).

19. Ford, L. S. & Hoffmann, R. S. *Potos flavus*. *Mammalian Species* **321**, 1-9 (1988).

20. Aguiar, L. M. *et al.* Diet of brown-nosed coatis and crab-eating raccoons from a mosaic landscape with exotic plantations in southern Brazil. *Studies on Neotropical Fauna and Environment* **46**, 153-161 (2011).

21. Gatti, A., Bianchi, R., Rosa, C. R. X. & Mendes, S. L. Diet of two sympatric carnivores, *Cerdocyon thous* and *Procyon cancrivorus*, in a restinga area of Espirito Santo State, Brazil. *Journal of Tropical Ecology* **22**, 227-230 (2006).

22. Bartels, M. A. & Thompson, D. P. *Spermophilus lateralis*. *Mammalian Species* **440**, 1-8 (1993).

23. Wible, J. R. On the cranial osteology of the short-tailed opossum *Monodelphis brevicaudata* (Didelphidae, Marsupialia). *Annals of the Carnegie Museum* **72**, 137-202 (2003).

24. Kielan-Jaworowska, Z., Cifelli, R. L. & Luo, Z.-X. *Mammals from the Age of Dinosaurs: Origins, Evolution, and Structure*. (Columbia University Press, 2004).

25. Luckett, W. P. in *Mammal Phylogeny: Mesozoic Differentiation, Multituberculates, Monotremes, Early Therians, and Marsupials* (eds Fredrick S. Szalay, Michael J. Novacek, & Malcolm C. McKenna) 182-204 (Springer-Verlag, 1993).

26. O'Leary, M. A. *et al.* The placental mammal ancestor and the post–K-Pg radiation of placentals. *Science* **339**, 662-667 (2013).

27. Lillegraven, J. A. & Bieber, S. L. Repeatability of measurements of small mammalian fossils with an industrial measuring microscope. *Journal of Vertebrate Paleontology* **6**, 96-100 (1986).

28. Szalay, F. S. & Trofimov, B. A. The Mongolian Late Cretaceous Asiatherium, and the early phylogeny and paleobiogeography of Metatheria. *Journal of Vertebrate Paleontology* **16**, 474-509 (1996).

29. Voss, R. S. & Jansa, S. A. Phylogenetic relationship and classification of didelphid marsupials, an extant radiation of New World metatherian mammals. *Bulletin of the American Museum of Natural History* **322**, 1-177 (2009).

30. Horovitz, I. *et al.* Cranial anatomy of the earliest marsupials and the origin of opossums. *PLoS ONE* **4**, 1-9 (2009).

31. Marshall, L. G., Muizon, C. d. & Sogogneau-Russell, D. Pucadelphys andinus (Marsupialia, Mammalia) from the early Paleocene of Bolivia. *Mem./Museum nat. d'histoire naturelle. Ser. Paleontologie* (1995).

32. Rougier, G. W., Wible, J. R. & Novacek, M. J. Implications of *Deltatheridium* for early marsupial history. *Nature* **396**, 459-463 (1998).

33. Sánchez-Villagra, M. *et al.* Exceptionally preserved North American Paleogene metatherians: adaptations and discovery of a major gap in the opossum fossil record. *Biology Letters* **3**, 318-322 (2007).

34. Krause, D. W. *et al.* First cranial remains of a gondwanatherian mammal reveal remarkable mosaicism. *Nature* **515**, 512-517 (2014).

35. Kemp, T. S. & 1982. *Mammal-like Reptiles and the Origin of Mammals*. (Academic Press, 1982).

36. Whidden, H. P. Extrinsic snout musculature in Afrotheria and Lipotyphla. *Journal of Mammalian Evolution* **9**, 161-184 (2002).

37. Engelmann, R. K. & Croft, D. A. A new species of small-bodied sparassodont (Mammalia, Metatheria) from the middle Miocene locality of Quebrada Honda, Bolivia. *Journal of Vertebrate Paleontology* **34**, 672-688 (2015).

38. Sánchez-Villagra, M. & Asher, R. J. Cranio-sensory adaptations in small faunivorous semiaquatic mammals, with special reference to olfaction and the trigeminal system. *Mammalia* **66**, 93-109 (2002).

39. Crumpton, N. & Thompson, R. S. The holes of the moles: osteological correlates of the trigeminal nerve in Talpidae. *Journal of Mammalian Evolution* **20**, 213-225 (2013).

40. Muizon, C., Cifelli, R. L. & Céspedes, R. The origin of dog-like borhyaenoid marsupials of South America. *Nature* **389**, 486-489 (1997).

41. Muizon, C. *Mayulestes ferox*, a borhyaenoid (Metatheria, Mammalia) from the early Palaeocene of Bolivia. Phylogenetic and palaeobiologic implications. *Geodiversitas* **20**, 19-142 (1998).

42. Abdala, F., Flores, D. A. & Giannini, N. P. Postweaning ontogeny of the skull of *Didelphis albiventris*. *Journal of Mammalogy* **82**, 190-200 (2001).

43. Astúa, D. Cranial sexual dimorphism in New World marsupials and a test of Rensch's rule in Didelphidae. *Journal of Mammalogy* **91**, 1011-1024 (2010).

44. Segall, W. The middel ear of *Dromiciops*. *Acta Anatomica* **72**, 489-501 (1969).

45. Clemens, W. A. Fossil mammals of the type Lance Formation, Wyoming: Part II. Marsupialia. *University of California Publications in Geological Sciences* **62**, 1-122 (1966).

46. Clemens, W. A. in *Mesozoic Mammals: The First Two-Thirds of Mammalian History* (eds Jason A. Lillegraven, Zofia Kielan-Jaworowska, & William A. Clemens) 192-220 (University of California Press, 1979).

47. Gabbert, S. L. Basicranial anatomy of Herpetotherium (Marsupialia: Didelphimorphia) from the Eocene of Wyoming. *Amer. Museum Novitates* **3235**, 1-13 (1998).

48. Wible, J. R. The internal carotid artery in early eutherians. *Acta Palaeontologica Polonica* **28**, 281-293 (1983).

49. Wible, J. R. Transformations in the extracranial course of the internal carotid artery in mammalian phylogeny. *Journal of Vertebrate Paleontology* **6**, 313-325 (1986).

50. Muizon, C. La fauna de mamiferos de Tiupampa (paleoceno inferior, formacion Santa Lucia), Bolivia. *Fósiles y facies de Bolivia. I Vertebrados, Revista Técnica YPFB* **12**, 575-624 (1991).

51. Muizon, C. A new carnivorous marsupial from the Palaeocene of Bolivia and the problem of marsupial monophyly. *Nature* **370**, 208-211 (1994).

52. Kielan-Jaworowska, Z. & Nessov, L. A. On the metatherian nature of the Deltatheroida, a sister group of the Marsupialia. *Lethaia* **23**, 1-10 (1990).

53. Szalay, F. & Trofimov, B. The Mongolian Late Cretaceous Asiatherium, and the early phylogeny and paleobiogeography of Metatheria. *Journal of Vertebrate Paleontology* **16**, 474-509 (1996).

54. Novacek, M. J. in *The skull* Vol. 2 (eds J. Hanken & Brian K. Hall) 438-545 (The University of Chicago Press, 1993).

55. Macintyre, G. T. in *Evolutionary biology* Vol. 6 (eds T Dobzhansky, M Hecht, & W Steere) 275-303 (Springer, 1972).

56. Patterson, B. The auditory region of the borhyaenid marsupial *Cladosictis*. *Breviora* **217**, 1-9 (1965).

57. Wible, J. R. Petrosals of Late Cretaceous marsupials from North America and a cladistic analysis of the petrosal in therian mammals. *Journal of Vertebrate Paleontology* **10**, 183-205 (1990).

58. Archibald, J. D. Oldest known eutherian stapes and a marsupial petrosal bone from the Late Cretaceous of North America. *Nature* **281**, 669-670 (1979).

59. Wible, J. R., Rougier, G. W., Novacek, M. J. & McKenna, M. C. Earliest eutherian ear region: a petrosal referred to *Prokennalestes* from the Early Cretaceous of Mongolia. *American Museum Novitates* **3322**, 1-44 (2001).

60. Ekdale, E. G., Archibald, J. D. & Averianov, A. O. Petrosal bones of placental mammals from the Late Cretaceous of Uzbekistan. *Acta Palaeontologica Polonica* **49**, 161-176 (2004).

61. Ladevèze, S. Metatherian petrosals from the Late Paleocene of Itaboraí, Brazil, and their phylogenetic implications. *Journal of Vertebrate Paleontology* **24**, 202-213 (2004).

62. Ladevèze, S. Petrosal bones of metatherian mammals from the Late Paleocene of Itaboraí (Brazil), and a cladistic analysis of petrosal features in metatherians. *Zoological Journal of the Linnean Society* **150**, 85-115 (2007).

63. Ladevèze, S. & de Muizon, C. The auditory region of early Paleocene Pucadelphydae (Mammalia, Metatheria) from Tiupampa, Boliva, with phylogenetic implications. *Palaeontology* **50**, 1123-1154 (2007).

64. Kielan-Jaworowska, Z. Evolution of the therian mammals in the Late Cretaceous of Asia. Part IV. Endocranial casts of eutherian mammals. *Palaeontologica Polonica* **46**, 157-171 (1984).

65. Meng, J. & Fox, R. C. Therian petrosals from the Oldman and Milk River formations (Late Cretaceous), Alberta, Canada. *Journal of Vertebrate Paleontology* **15**, 122-130 (1995).

66. Meng, J. & Fox, R. C. Osseous inner ear structures and hearing in early marsupials and placentals. *Zoological Journal Of The Linnean Society* **115**, 47-71 (1995).

67. McKenna, M. C., Kielan-Jaworowska, Z. & Meng, J. Earliest eutherian mammal skull, from the Late Cretaceous (Coniacian) of Uzbekistan. *Acta Palaeontologica Polonica* **45**, 1-54 (2000).

68. Wible, J., Rougier, G., Novacek, M. & McKenna, M. Earliest eutherian ear region: a petrosal referred to Prokennalestes from the Early Cretaceous of Mongolia. *American Museum Novitates* **3322**, 1-44 (2001).

69. Ekdale, E. G. & Rowe, T. Morphology and variation within the bony labyrinth of zhelestids (Mammalia, Eutheria) and other therian mammals. *Journal of Vertebrate Paleontology* **31**, 658-675 (2011).

70. Ekdale, E. G. Comparative anatomy of the bony labyrinth (inner ear) of placental mammals. *PLoS ONE* **8**, e66624 (2013).

71. Luo, Z.-X., Ruf, I. & Martin, T. The petrosal and inner ear of the Late Jurassic cladotherian mammal *Dryolestes leiriensis* and implications for ear evolution in therian mammals. *Zoological Journal of the Linnean Society* **166**, 433-463 (2012).

72. David, R. *et al.* Motion from the past. A new method to infer vestibular capacities of extinct species. *Comptes Rendus Palevol* **9**, 397-410 (2010).

73. Rodgers, J. C. *Comparative morphology of the vestibular semicircular canals in therian mammals* Ph.D. thesis, The University of Texas at Austin, (2011).

74. Lofgren, D. L. Upper premolar configuration of *Didelphodon vorax* (Mammalia, Marsupialia, Stagodontidae). *Journal of Paleontology* **66**, 162-164 (1992).

75. Clemens, W. A. A mandible of *Didelphodon vorax* (Marsupialia, Mammalia). *Los Angeles County Museum, Contributions in Science* **133**, 1-11 (1968).

76. Fox, R. C. & Naylor, B. G. A new species of *Didelphodon* Marsh (Marsupialia) from the Upper Cretaceous of Alberta, Canada: Paleobiology and phylogeny. *Neues Jahrbuch für Geologie und Paläontologie, Abhandlungen* **172**, 357-380 (1986).

77. Rougier, G. W., Davis, B. & Novacek, M. J. A deltatheroidan mammal from the Upper Cretaceous Baynshiree Formation, eastern Mongolia. *Cretaceous Research* **52**, 167-177 (2015).

78. Wible, J. R. & Hopson, J. A. in *Mammal Phylogeny* (eds F. S. Szalay, M.J. Novacek, & M. C. McKenna) 45-62 (Springer, 1993).

79. Evans, H. & Christensen, G. Miller's anatomy of the dog. *Philadelphia: WB Saunders* **780**, 836 (1979).

80. Goloboff, P. A., Farris, J. S. & Nixon, K. C. TNT, a free program for phylogenetic analysis. *Cladistics* **24**, 774-786 (2008).
