# Supplementary material for: A large carnivorous mammal from the Late Cretaceous and the North American origin of marsupials
Source: Nat Commun. 2016 Dec 8;7:13734. doi: 10.1038/ncomms13734 (PMC5155139; doi:10.1038/ncomms13734)
Supplement: Supplementary Data 1 — Phylogenetic data matrix [file ncomms13734-s2.docx]

**Supplementary Data 1**

**Phylogenetic data matrix**—Below is the data matrix in TNT format, except that polymorphic entries are here coded as: A = [0&1], B = [1&2], C = [2&3], D = [0&2], E = [0&1&2].

nstates 8 ;

xread 'Data saved from TNT'

164 48

'Amphitherium_Dryolestidae' 0020000110?000??0??0?000??00???0??00???0000000000000?000010200100000000000000000001010010?0?000?0???????????????????????????????????????????????????????????????????

Peramus 0012000??10000000012000000010000??00???0000100100001000000000?0???0000?021001???????????????????????????????????????????????????????????????????????????????????????

Vincelestes 3002102100000000002200010021000000010001110100000001000000011?1??0100000011000000000000101000000000000000000000000000000000000000000000000000?0000000000000000000000

Kielantherium 1??1000???????1000A210002201100110010001????0?A01001000000001?0??????0???1000???????????????????????????????????????????????????????????????????????????????????????

Potamotelses

????00????????00112200002101010000021001???????0110101000?011???????????????????????????????????????????????????????????????????????????????????????????????????????

Kermackia

?0??00????????111?A000002101000000121001??????12110101000?020?1?????????????????????????????????????????????????????????????????????????????????????????????????????

Holoclemensia

?01201??????0?110121001112110100101300010?????121111010010121???????????????????????????????????????????????????????????????????????????????????????????????????????

Pappotherium

???10?????????110010000122011100101200010??????11101?1010?01????????????????????????????????????????????????????????????????????????????????????????????????????????

Deltatheridium 200102111120001101120000221100011012000111110000100101010000111?1110011011111111010010000100000?10?????????21???1?1A0121?1221201101000?00?00??212110011200????000???

Deltatheroides 2001021??1?0001100120000221100011012000111?100001001010100001?1???1??1???1111?1?010???????0?????????????????????????????????????????????????????????????????????????

Tsagandelta 2??10B1????????????????????????1???????????110?11001??010?001?0???10?1?011111???????????????????????????????????????????????????????????????????????????????????????

Oklatheridium

???102????????11011D0000221100011012??011??????11101?1010000????????????????????????????????????????????????????????????????????????????????????????????????????????

Sulestes 2001021??10?0011011200002211000110120001111110011101010100001?0???10?1??11A11?1??11??000????0????0????????????????????????22?20110??00??0?0?????1110?1120???????0???

Atokatheridium

???B02????????11001200002211000110121001???????01001?0010000????????????????????????????????????????????????????????????????????????????????????????????????????????

Nanocuris

???1011???????110??200002211000110A21001???11??01101000100001?1??????1???111????????????????????????????????????????????????????????????????????????????????????????

Lotheridium 200102111120001111120000221100010002000111111000100101010000110?1110011011111?11010010000100000000??????????????1110?1?1??22?????????000?????????????????0010??????0

Gurlin_Tsav_Skull 20010211112000111010020112220101102300010????????????????????????????????????111010010000100110011??00101?0221211110012113???2??110100?0?1?11?21?111????000001???011

Pariadens

???102????????012?1000112221010111130101???????112B2021100100???????????????????????????????????????????????????????????????????????????????????????????????????????

Kokopellia 2001011???????101?A20000221101001123?101??0110011211121000010?0???1??1???111????????????????????????????????????????????????????????????????????????????????????????

Anchistodelphys

???1011???????11101000AA2222010011231101???????11212121001020???????????????????????????????????????????????????????????????????????????????????????????????????????

Iugomortiferum

????11????????001??200002221010011231101???????11212021010020???????????????????????????????????????????????????????????????????????????????????????????????????????

Aenigmadelphys 20?1011???????11202000012211110011231101?????001111212101?000???????????????????????????????????????????????????????????????????????????????????????????????????????

Didelphodon 2121021111010111201D02012222010110241101011110021222021110100?0??11111101111111111111????10011100?110???1??22?2111100101121211?1100100?011120?202110??02011?01000011

Eodelphis 21210211?1?00011201202002222010110231101011110021212021110100?0??111111011111?????????????001???????????????????111001B1??????????????00???B??B1?????????1???????0??

Pediomys 2001011????00011300000A222220100112411010??11001122202111002010???1111?A11111????1?????????0111?1?????1????22??????????B?2121201100100??100B??B12110?11200??????????

Albertatherium

???101????????11101000A122211100112311010??????11212?2111?12????????????????????????????????????????????????????????????????????????????????????????????????????????

Alphadon 2001011??1?00011101000112221110011231101?001000112120211100201011111011011111?????1????????01???????????????????????????????????????????????????????????????????????

Turgidodon 2101111????00011101000112221010011231101???1?0011212?21110020?0???????????????????1????????????????????????22??????????1??121201100?00?0010B??B121100?121???????????

Glasbius 2001101????00011101000A2222B0100112411111??111011222021110121?1???????????111???????????????????????????????????????????????????????????????????????????????????????

Asiatherium 2021011??1100011B001000222110100112300110???00011212021010020?0???10010111111??1?10010100100111010??0??????2E1B?1110?1211?????0?10010000???B?2?0?110011?????????????

Mayulestes 2001011001200011101100012222010110231001001110011122111010010?0?????????????1111110110000100011010010??00?021111111011?10113?20?1010000000111?1??1111112?00101?10100

Borhyaenids 2001021111B00011101000002222010110221001011110011112111110000?0??1100110111111111101100111000001101110100?0221111110012101131201101000100101102B2111111200100?????00

Pucadelphys 2001011001200011100102112122111010241101001110011222121010120?0???11011011111101010011000100011010?100100?02111111101111011212011000000000101?222111111210?100111000

Andinodelphys 20010110012000111000021122221110102311010011?0011222111110120?0???110??01??1?101010111???1000110??1100110??2B111111011B10112?201100A00?001?01??2211111?210??00011000

Jaskhadelphys

????01????????11101002112221111110131101????????????????????????????????????????????????????????????????????????????????????????????????????????????????????????????

Marmosa 20010110012000112002021112221110100311010011010112220201100201011111111111110111011111100100111011110011010221111110111111121201112000100012002121100112101110????00

Didelphis 20010110012000112002021111221110100311010011010112220201100201011111111111110111011111101100111011110011010221111110111111121201112000100012002121100112101010100100

Dasyurids 20010111110000112012021022221110100301011111000112220211110201011111111111110111A10111101100111100110011010221111110112111121201112000101012022221100112101101????00

Dromiciops 2021011011000011201201002222010010041101100100011212020111021?01111111101111110101111110010011100111001001022121111011211313?21?1020000000?2022221100012101?00100000

Prokennalestes 0012000??0110211100010011201000011130001000000011101110010020?0???0000?021001???011???????0?0?????????0????11???1????????101010010B200?00100??A100000?0010??????????

Bobolestes 0012000???????11110111002201000011230001000101111111110010020?0????001?021001???????????????????????????????????????????????????????????????????????????????????????

Asioryctes 1012001010021211400101002201000011230101000000021111110010020?0???00010021111101011010101011001010011100??023120110101120103?2111022110101000112?100001A10012?????00

Kennalestes 1012001?1002121140011100220100001123011101000002111111001002000??00001?021111?01011010?0100100101001110???02312?110?011201?3021110221101??000112?100001A100???????00

Zalambdalestes 1012001120021211402201002111010001230101110100221221010010120?0???000A010111110101001001000100A0110111000?023120110B01B20113021110221101010001111100001A10012?????00

Aspanlestes 0012000??01102114000110021110100112311110??1?112121101001012000???00000021111?0??1????????0??????????????????????????????10201011???00????0????10110??100???????????

Leptictids 1012001121021211402201002111010011240111010101221211110010120000001001112111010101111101101100101011110011122120111201110113021110220001010100121000111110112?????00

Herpetotherium 20010110?1200011201200112222111010A31101001100011212021110020?????1111101?1111?1?111?1?0?0001??????????????22?2??????1??1103?2?011120???0??2???22110??121???201001?0

'Mimo_Peradectes' 2011011??1200011101200112222010110A311010011100111A201A110000????????????????????110??100000???????????10??22?11????????1112120111120???0??20??12110??121?????1001??
