# Supplementary material for: A large carnivorous mammal from the Late Cretaceous and the North American origin of marsupials
Source: Nat Commun. 2016 Dec 8;7:13734. doi: 10.1038/ncomms13734 (PMC5155139; doi:10.1038/ncomms13734)
Supplement: Supplementary Data 2 — Metatherian synapomorphies [file ncomms13734-s3.docx]

**Supplementary Data 2**

**Metatherian synapomorphies**—Below we append the TNT output of synapomorphies common among the 18 EPTs from the parsimony analysis of our data matrix. Note that TNT (and the list below) uses a character numbering scheme that starts with 0 instead of 1, but in the Supplementary Note 2 our character numbering scheme starts with 1, the typical convention and that used by Rougier et al. (2015).

Peramus :

Char. 9: 0 --> 1

Char. 76: 0 --> 1

Vincelestes :

Char. 0: 12 --> 3

Char. 2: 1 --> 0

Char. 4: 0 --> 1

Char. 6: 1 --> 2

Char. 8: 1 --> 0

Char. 23: 0 --> 1

Char. 26: 0 --> 2

Char. 40: 0 --> 1

Char. 84: 1 --> 0

Kielantherium :

Char. 14: 0 --> 1

Char. 20: 0 --> 1

Char. 25: 0 --> 2

Char. 28: 0 --> 1

Char. 31: 0 --> 1

Potamotelses :

Char. 17: 0 --> 1

Char. 29: 0 --> 1

Holoclemensia :

Char. 17: 0 --> 1

Char. 19: 0 --> 1

Char. 22: 0 --> 1

Char. 24: 2 --> 1

Char. 58: 0 --> 1

Pappotherium :

Char. 28: 0 --> 1

Sulestes :

Char. 144: 2 --> 1

Nanocuris :

Char. 5: 2 --> 1

Char. 49: 0 --> 1

Lotheridium :

Char. 16: 0 --> 1

Char. 32: 1 --> 0

Char. 34: 1 --> 0

Char. 96: 1 --> 0

Gurlin_Tsav_Skull :

Char. 21: 0 --> 2

Char. 24: 2 --> 1

Char. 97: 0 --> 1

Char. 104: 0 --> 1

Char. 121: 1 --> 3

Char. 129: 0 --> 1

Char. 162: 0 --> 1

Char. 163: 0 --> 1

Pariadens :

Char. 14: 1 --> 0

Char. 22: 0 --> 1

Char. 34: 2 --> 1

Char. 36: 1 --> 0

Char. 56: 1 --> 0

Kokopellia :

Char. 26: 2 --> 1

Char. 51: 2 --> 1

Char. 52: 0 --> 1

Char. 56: 1 --> 0

Char. 59: 2 --> 1

Anchistodelphys :

Char. 27: 1 --> 2

Char. 52: 0 --> 1

Char. 55: 1 --> 0

Char. 56: 1 --> 0

Char. 57: 0 --> 1

Iugomortiferum :

Char. 4: 0 --> 1

Char. 14: 1 --> 0

Aenigmadelphys :

Char. 16: 1 --> 2

Char. 18: 1 --> 2

Char. 26: 2 --> 1

Char. 28: 0 --> 1

Char. 49: 2 --> 1

Char. 52: 0 --> 1

Char. 55: 1 --> 0

Char. 59: 2 --> 0

Didelphodon :

Char. 11: 0 --> 1

Char. 13: 0 --> 1

Char. 35: 3 --> 4

Char. 50: 1 --> 2

Char. 118: 2 --> 0

Char. 143: 1 --> 0

Eodelphis :

Char. 23: 1 --> 0

Pediomys :

Char. 16: 1 --> 3

Char. 18: 1 --> 0

Albertatherium :

Char. 28: 0 --> 1

Char. 58: 0 --> 1

Alphadon :

Char. 22: 0 --> 1

Char. 28: 0 --> 1

Char. 44: 1 --> 0

Char. 68: 1 --> 0

Turgidodon :

Char. 1: 0 --> 1

Char. 4: 0 --> 1

Char. 22: 0 --> 1

Char. 152: 0 --> 1

Glasbius :

Char. 4: 0 --> 1

Char. 5: 1 --> 0

Char. 38: 0 --> 1

Char. 40: 0 --> 1

Char. 45: 0 --> 1

Char. 58: 0 --> 1

Char. 60: 0 --> 1

Char. 62: 0 --> 1

Asiatherium :

Char. 2: 0 --> 2

Char. 10: 0 --> 1

Char. 18: 1 --> 0

Char. 19: 0 --> 1

Char. 23: 1 --> 2

Char. 38: 0 --> 1

Char. 44: 1 --> 0

Char. 70: 1 --> 0

Char. 71: 0 --> 1

Char. 143: 1 --> 0

Mayulestes :

Char. 19: 0 --> 1

Char. 59: 2 --> 1

Char. 98: 1 --> 0

Char. 142: 2 --> 1

Char. 161: 0 --> 1

Borhyaenids :

Char. 23: 1 --> 0

Char. 35: 3 --> 2

Char. 59: 2 --> 0

Char. 87: 0 --> 1

Char. 88: 0 --> 1

Char. 93: 1 --> 0

Char. 95: 0 --> 1

Char. 100: 0 --> 1

Char. 134: 0 --> 1

Char. 154: 0 --> 1

Pucadelphys :

Char. 19: 0 --> 1

Char. 25: 2 --> 1

Char. 35: 3 --> 4

Char. 53: 1 --> 2

Char. 83: 1 --> 0

Char. 158: 0 --> 1

Andinodelphys :

Char. 103: 0 --> 1

Jaskhadelphys :

Char. 27: 2 --> 1

Char. 34: 2 --> 1

Didelphis :

Char. 25: 2 --> 1

Char. 155: 1 --> 0

Dasyurids :

Char. 7: 0 --> 1

Char. 23: 1 --> 0

Char. 36: 1 --> 0

Char. 40: 0 --> 1

Char. 41: 0 --> 1

Char. 57: 0 --> 1

Char. 82: 1 --> 0

Char. 95: 0 --> 1

Char. 136: 0 --> 1

Char. 157: 0 --> 1

Dromiciops :

Char. 2: 0 --> 2

Char. 21: 0 --> 1

Char. 23: 1 --> 0

Char. 35: 3 --> 4

Char. 40: 0 --> 1

Char. 54: 1 --> 0

Char. 57: 0 --> 1

Char. 60: 0 --> 1

Char. 78: 1 --> 0

Char. 123: 2 --> 3

Char. 126: 0 --> 1

Char. 149: 1 --> 0

Prokennalestes :

Char. 21: 1 --> 0

Char. 23: 0 --> 1

Char. 24: 2 --> 1

Char. 34: 2 --> 1

Char. 43: 1 --> 0

Char. 50: 1 --> 0

Bobolestes :

Char. 17: 0 --> 1

Char. 19: 0 --> 1

Asioryctes :

Char. 7: 1 --> 0

Char. 86: 0 --> 1

Char. 90: 0 --> 1

Kennalestes :

Char. 20: 0 --> 1

Char. 38: 0 --> 1

Zalambdalestes :

Char. 32: 1 --> 0

Char. 40: 0 --> 1

Char. 50: 1 --> 2

Char. 52: 1 --> 0

Char. 72: 2 --> 0

Char. 82: 1 --> 0

Char. 88: 1 --> 0

Char. 97: 0 --> 1

Char. 143: 2 --> 1

Aspanlestes :

Char. 25: 2 --> 1

Char. 26: 0 --> 1

Char. 29: 0 --> 1

Char. 36: 0 --> 1

Char. 38: 0 --> 1

Char. 49: 1 --> 2

Char. 58: 0 --> 1

Char. 146: 0 --> 1

Leptictids :

Char. 9: 0 --> 1

Char. 35: 3 --> 4

Char. 38: 0 --> 1

Char. 45: 0 --> 1

Char. 66: 0 --> 1

Char. 70: 0 --> 1

Char. 76: 1 --> 0

Char. 83: 0 --> 1

Char. 85: 0 --> 1

Char. 90: 0 --> 1

Char. 98: 0 --> 1

Char. 104: 0 --> 1

Char. 106: 0 --> 1

Char. 108: 3 --> 2

Char. 114: 0 --> 1

Char. 119: 2 --> 1

Char. 132: 1 --> 0

Char. 133: 1 --> 0

Char. 139: 0 --> 1

Char. 141: 1 --> 0

Char. 145: 1 --> 0

Char. 148: 0 --> 1

Char. 149: 0 --> 1

Char. 154: 0 --> 1

Herpetotherium :

Char. 122: 1 --> 0

Char. 123: 2 --> 3

Char. 127: 1 --> 0

'Mimo_Peradectes' :

Char. 2: 0 --> 1

Char. 16: 2 --> 1

Char. 31: 0 --> 1

Char. 44: 0 --> 1

Char. 49: 2 --> 1

Char. 53: 2 --> 1

Char. 59: 2 --> 0

Char. 83: 1 --> 0

Node 50 :

Char. 6: 0 --> 1

Char. 59: 0 --> 1

Char. 74: 0 --> 1

Node 51 :

Char. 0: 0 --> 1

Char. 35: 0 --> 1

Char. 39: 0 --> 1

Char. 60: 0 --> 1

Node 52 :

Char. 25: 0 --> 1

Char. 35: 1 --> 2

Char. 49: 0 --> 1

Char. 53: 0 --> 1

Node 53 :

Char. 14: 0 --> 1

Char. 15: 0 --> 1

Char. 19: 2 --> 0

Char. 34: 0 --> 1

Char. 47: 0 --> 2

Char. 59: 1 --> 2

Node 54 : Metatheria

Char. 23: 0 --> 1

Char. 29: 0 --> 1

Node 55 : Theria

Char. 25: 1 --> 2

Char. 32: 0 --> 1

Char. 35: 2 --> 3

Char. 50: 0 --> 1

Char. 56: 0 --> 1

Node 56 : Deltatheroida

Char. 35: 3 --> 2

Char. 50: 1 --> 0

Char. 56: 1 --> 0

Node 57 :

Char. 2: 1 --> 0

Char. 3: 2 --> 1

Char. 47: 2 --> 1

Node 58 :

Char. 62: 0 --> 1

Node 59 :

Char. 47: 1 --> 0

Node 60 :

Char. 49: 1 --> 0

Node 61 :

Char. 17: 0 --> 1

Char. 19: 0 --> 2

Char. 23: 1 --> 0

Char. 29: 1 --> 0

Char. 31: 0 --> 1

Char. 40: 0 --> 1

Node 62 :

Char. 36: 0 --> 1

Char. 53: 1 --> 0

Node 63 : (‘Gurlin Tsav skull’+ SA Marsupialiformes)

Char. 10: 0 --> 2

Char. 27: 1 --> 2

Char. 31: 0 --> 1

Char. 140: 0 --> 1

Char. 147: 0 --> 1

Node 64 : Marsupialiformes

Char. 34: 1 --> 2

Char. 51: 1 --> 2

Char. 54: 0 --> 1

Char. 93: 0 --> 1

Char. 108: 1 --> 2

Node 65 : Stagodontidae

Char. 5: 1 --> 2

Char. 16: 1 --> 2

Char. 31: 0 --> 1

Char. 58: 0 --> 1

Char. 59: 2 --> 0

Node 66 : (NA Marsupialiformes, Marsupialia)

Char. 36: 0 --> 1

Char. 37: 0 --> 1

Char. 67: 0 --> 1

Char. 68: 0 --> 1

Char. 83: 0 --> 1

Node 67 : (Asiatherium + NA Marsupialiformes, Marsupialia)

Char. 33: 0 --> 1

Char. 49: 1 --> 2

Char. 53: 1 --> 2

Char. 86: 0 --> 1

Char. 94: 0 --> 1

Node 68 :

Char. 15: 1 --> 0

Char. 19: 0 --> 2

Char. 23: 1 --> 0

Char. 55: 1 --> 0

Node 69 :

Char. 21: 0 --> 2

Char. 27: 1 --> 2

Char. 33: 1 --> 0

Char. 47: 1 --> 2

Node 70 :

Char. 23: 1 --> 2

Char. 35: 3 --> 4

Char. 50: 1 --> 2

Node 71 :

Char. 7: 1 --> 0

Char. 8: 1 --> 0

Char. 50: 1 --> 2

Char. 94: 0 --> 1

Char. 108: 2 --> 1

Char. 116: 0 --> 1

Char. 138: 0 --> 1

Node 72 : SA Marsupialiformes

Char. 36: 0 --> 1

Char. 83: 0 --> 1

Char. 110: 2 --> 1

Node 73 :

Char. 18: 1 --> 0

Char. 31: 1 --> 0

Node 74 :

Char. 21: 0 --> 2

Char. 22: 0 --> 1

Char. 28: 0 --> 1

Char. 30: 0 --> 1

Char. 37: 0 --> 1

Node 75 :

Char. 8: 1 --> 0

Char. 18: 1 --> 0

Char. 24: 2 --> 1

Char. 45: 0 --> 1

Char. 54: 1 --> 0

Char. 118: 2 --> 1

Char. 141: 2 --> 0

Char. 156: 0 --> 1

Node 76 :

Char. 21: 0 --> 2

Char. 50: 1 --> 2

Char. 71: 0 --> 1

Char. 76: 1 --> 0

Node 77 :

Char. 22: 0 --> 1

Char. 103: 0 --> 1

Char. 129: 0 --> 1

Char. 161: 0 --> 1

Node 78 : Marsupialia

Char. 7: 1 --> 0

Char. 16: 1 --> 2

Char. 19: 0 --> 2

Char. 27: 1 --> 2

Char. 33: 1 --> 0

Char. 34: 2 --> 0

Char. 44: 1 --> 0

Char. 116: 0 --> 1

Char. 130: 0 --> 2

Char. 131: 1 --> 0

Char. 137: 1 --> 0

Char. 152: 0 --> 1

Char. 158: 0 --> 1

Node 79 :

Char. 47: 2 --> 1

Char. 74: 1 --> 0

Char. 75: 1 --> 0

Node 80 :

Char. 0: 1 --> 0

Char. 6: 1 --> 0

Char. 10: 0 --> 1

Char. 20: 0 --> 1

Char. 125: 2 --> 1

Node 81 :

Char. 11: 0 --> 1

Char. 13: 0 --> 2

Char. 21: 0 --> 1

Char. 33: 0 --> 1

Char. 34: 1 --> 2

Char. 130: 0 --> 2

Char. 131: 0 --> 2

Node 82 :

Char. 11: 1 --> 2

Char. 12: 0 --> 1

Char. 19: 0 --> 1

Char. 108: 1 --> 3

Char. 123: 2 --> 3

Char. 126: 0 --> 1

Char. 132: 0 --> 1

Char. 133: 0 --> 1

Char. 135: 0 --> 1

Char. 143: 1 --> 2

Node 83 :

Char. 8: 1 --> 2

Char. 18: 0 --> 2

Char. 19: 1 --> 2

Char. 25: 2 --> 1

Char. 26: 0 --> 1

Char. 29: 0 --> 1

Char. 46: 0 --> 2

Char. 49: 1 --> 2

Char. 58: 0 --> 1

Char. 71: 0 --> 1

Char. 87: 0 --> 1

Char. 122: 0 --> 1

Node 84 :

Char. 89: 1 --> 0

Char. 130: 2 --> 1

Char. 131: 0 --> 2
